# Supplementary material for: Discovery of type II polyketide synthase-like enzymes for the biosynthesis of cispentacin
Source: Nat Commun. 2023 Dec 6;14:8065. doi: 10.1038/s41467-023-43731-z (PMC10698177; doi:10.1038/s41467-023-43731-z)
Supplement: Supplementary file 1 — Supplementary Information [file 41467_2023_43731_MOESM1_ESM.pdf]

Supplementary Information

Discovery of Type II Polyketide Synthase-like Enzymes for the Biosynthesis of Cispentacin

Genki Hibi,[a] Taro Shiraishi,[a,b] Tatsuki Umemura,[a] Kenji Nemoto,[a] Yusuke Ogura,[a] Makoto Nishiyama,[a,b] Tomohisa Kuzuyama*[a,b]

[a]Graduate School of Agricultural and Life Sciences, The University of Tokyo, 1-1-1 Yayoi, Bunkyo-ku, Tokyo 113-8657, Japan; [b]Collaborative Research Institute for Innovative Microbiology, The University of Tokyo, 1-1-1 Yayoi, Bunkyo-ku, Tokyo 113-8657, Japan.

Table of Contents

Supplementary Tables 1

Supplementary Table 1. Deduced function of the *amc* genes 1

Supplementary Table 2. List of primers used in this study. 2

Supplementary Table 3. Strains used in this study. 3

Supplementary Table 4. Vectors used in this study. 3

Supplementary Table 5. The characterization of chemical and biomolecular materials. 4

Supplementary Figures 5

Supplementary Figure 1. Comparison of type II PKS subfamilies. 5

Supplementary Figure 2. Biosynthetic pathway of CPC-ACP postulated in previous studies. ...6

Supplementary Figure 3. Schematic representation of the plasmid constructs. 6

Supplementary Figure 4. X-LC and MS analysis of DNFB-derivatized cispentacin. 8

Supplementary Figure 5. Production of cispentacin by *S. albus* G153/pSEamcB-H. 9

Supplementary Figure 6. SDS–PAGE analysis of purified recombinant proteins. 10

Supplementary Figure 7. Gel filtration of AmcF-AmcG. 11

Supplementary Figure 8. Detection of AmcB bound intermediate derived from malonyl-CoA. 12

Supplementary Figure 9. Substrate specificity of AmcH. 13

Supplementary Figure 10. HPLC analysis of AmcH activity. 14

Supplementary Figure 11. LC–ESI–HRMS analysis of 2-AmcB digested with thermolysin. ..15

Supplementary Figure 12. MS/MS spectrum of the PPant-eliminated ion of 2-AmcB. 16

Supplementary Figure 13. Phylogenetic analysis of KSs in the cispentacin cluster and various types of known KSs. 17

Supplementary Figure 14. Sequence analysis of AmcF and AmcG. 18

Supplementary Figure 15. AmcF–AmcG heterodimer fold prediction. 19

Supplementary Figure 16. Assessment of the dimer structure. 20

Supplementary Figure 17. Comparative analysis of CLF and cyclization factors (CYFs). 22

Supplementary Figure 18. Sequence analysis of AmcE. 24

Supplementary Figure 19. UV–vis spectra assay for AmcC. 25

Supplementary Figure 20. LC–MS analysis of cispentacin in the reconstituted reaction mixture. 26

Supplementary Figure 21. Amine donor specificity of AmcC. 27

Supplementary Figure 22. NADH-dependent reduction of 3-AmcB. 29
Supplementary Figure 23. Validation of the enzyme that catalyzes the reduction of 3-AmcB. 32
Supplementary Figure 24. LC–MS analysis of FDLA-derivatized cispentacins. 33
Supplementary Figure 25. Organization of *amcG* homolog-containing BGC. 35
Supplementary Figure 26. Representatives of the *amcG* homolog-containing BGCs for unknown natural products. 35
Supplementary Figure 27. FabI-catalyzed reduction. 36
Supplementary Figure 28A. ^{1}H NMR spectrum of 2,3-di(carbo-*tert*-butoxy)-2-cyclopentenone in $CDCl_3$ at 600 Hz. 37
Supplementary Figure 28B. ^{13}C NMR spectrum of 2,3-di(carbo-*tert*-butoxy)-2-cyclopentenone in $CDCl_3$ at 150 Hz. 38
Supplementary Figure 28C. HSQC spectrum of 2,3-di(carbo-*tert*-butoxy)-2-cyclopentenone in $CDCl_3$. 39
Supplementary Figure 28D. HMBC spectrum of 2,3-di(carbo-*tert*-butoxy)-2-cyclopentenone in $CDCl_3$. 40
Supplementary Figure 29A. ^{1}H NMR spectrum of 3-oxocyclopent-1-ene-1,2-dicarboxylic acid in DMSO-d_6 at 600 Hz. 41
Supplementary Figure 29B. ^{13}C NMR spectrum of 3-oxocyclopent-1-ene-1,2-dicarboxylic acid in DMSO-d_6 at 150 Hz. 42
Supplementary Figure 30. HRMS spectra of 3-oxocyclopent-1-ene-1,2-dicarboxylic acid. 43
Supplementary References 44

Supplementary Tables

Supplementary Table 1. Deduced function of the *amc* genes

ORFs	Size (aa)	Proposed function*	E-value	Homologous protein [origin]
AmcB	88	Acy carrier protein (ACP) for PKS	0.001	Acyl carrier protein [*Listeria welshimeri* serovar 6b str. SLCC5334]
AmcC	471	Aminotransferase	1.00E-34	Putrescine aminotransferase [*Cronobacter sakazakii* ATCC BAA-894]
AmcD	155	Thioesterase	8.00E-12	1,4-dihydroxy-2-naphthoyl-CoA hydrolase [*Parasynechococcus marenigrum* WH 8102]
AmcE	167	Dehydratase	3.00E-74	Coronafacic acid dehydratase [*Pseudomonas savastano*i pv. glycinea]
AmcF	397	Ketosynthase (KS) for PKS	2.00E-38	3-oxoacyl-[acyl-carrier-protein] synthase 2 [*Bacillus subtilis* subsp. subtilis str. 168]
AmcG	184	Hypothetical protein	-	**No significant similarity found**
AmcH	460	Adenylate-forming enzyme	5.00E-12	Putative acyl-CoA synthetase YngI [*Bacillus subtilis* subsp. subtilis str. 168]

The nucleotide sequence of the amc cluster is deposited in the DDBJ/EMBL/GenBank nucleotide sequence database under the accession number LC389220.*The predicted function of ORFs. E-Value and Homologous protein [origin] represent the results obtained by a BLATP search using the UniProtKB/Swiss-Prot (swissprot) database, with scoring parameters set to default (BLOSUM62, Existence: 11 Extension: 1, Conditional compositional score matrix adjustment).

Supplementary Table 2. List of primers used in this study.

Primer	Oligonucleotide sequence	Restriction site (underlined)
	E. coli	
AmcB-CHisfw	5'-GGAATTCCATATGAACCAGACGGAAGCCGCCAT-3'	*Nde*I
AmcB-CHisrv	5'-CCCAAGCTTGGCCGTCTCGAGTCGGGA-3'	*Hin*dIII
AmcC-NHisfw	5'-CGGGATCCACGTCCGCACCGGACTTCC-3'	*Bam*HI
AmcC-NHisrv	5'-CCCAAGCTTTCATGCGTGCGTCTCGAG-3'	*Hin*dIII
AmcD-NHisfw	5'-CGGGATCCACCGCCGCGCAGCCGGTACC-3'	*Bam*HI
AmcD-NHisrv	5'-CCCAAGCTTTCAACGCTGTGCACGGAC-3'	*Hin*dIII
AmcE-NHisfw	5'-CGGGATCCCACAGCGTTGACCCCGAC-3'	*Bam*HI
AmcE-NHisrv	5'-CCCAAGCTTTCACCACAGCTGCTCCCCGA-3'	*Hin*dIII
AmcH-NHisfw	5'-CGCGGATCCAAAGCAGCCCTCCTCCCCGA-3'	*Bam*HI
AmcH-NHisrv	5'-CCCAAGCTTTCACTTGTACCAGTCGATGGACCG-3'	*Hin*dII
sFabI-CHisfw	5'-GGCATATGAGTGGACTTCTCGCAGGCAAG-3'	*Nde*I
sFabI-CHisrv	5'-GGGAATTCCCGGCGCCGATGGCGTG-3'	*Eco*RI
eFabI-CHisfw	5'-GGCATATGGGTTTTCTTTCCGGTAAGCG-3'	*Nde*I
eFabI-CHisrv	5'-GGGAATTCCCTTTCAGTTCGAGTTCGTTC-3'	*Eco*RI
	S. albus	
pSEamcB-H-N	5’-GGGAAGCTTGAAGGGAATGCACGATGAACC-3'	*Hin*dII
pSEamcH-NHisfw	5’-GGGAAGCTTAGCAACGGAGGTACGGACATGCACCACCACCACCACCACCACCACAAAGCAGCCCTCCTCCC-3'	*Hin*dII
pSEamcH-Rv	5'-GGGTCTAGAGTCACTTGTACCAGTCGATGG-3'	*Xba*I
pSEAmcF-NHisfw	5'-CCCAAGCTTAGCAACGGAGGTACGGACATGCACCACCACCACCACCACCACCACGTGACGCCCGGCCCGCG-3'	*Hin*dII
pSEAmcG-rv	5'-CCCGCTCTAGAGCTCACCTCCGGCCGGCCG-3'	*Xba*I

Supplementary Table 3. Strains used in this study.

Strains	Characteristic(s)	Source/Reference
DH5α	Host for general cloning	Takara
BL21 (DE3)	Host for protein expression	Takara
BL21 (DE3)/pET26(+)-amcB/pACYCDuet-pptA2	expressing C-terminal His-tagged AmcB with pptA2	This study
BL21 (DE3)/pHis8-amcC	expressing N-terminal His-tagged AmcC	This study
BL21 (DE3)/pHis8-amcD	expressing N-terminal His-tagged AmcD	This study
BL21 (DE3)/pHis8-amcE	expressing N-terminal His-tagged AmcE	This study
BL21 (DE3)/pET26(+)-sFabI	expressing C-terminal His-tagged sFabI	This study
BL21 (DE3)/pET26(+)-eFabI	expressing C-terminal His-tagged eFabI	This study
Streptomyces sp. SN-C1	Cispentacin producer	Gift from Kumiai Chemical Industry Co., Ltd.
S. albus G153	Host for heterologous expression	Gift from Prof. Bradley S. Moore
S. albus G153/pSE101	Negative control of heterologous expression	This study
S. albus G153/pSEamcB-H	Recombinant strain/Cispentacin producer	This study
S. albus G153/pSEamcF-G	expressing N-terminal His-tagged AmcF and AmcG	This study
S. albus G153/pSEamcH	expressing N-terminal His-tagged AmcH	This study

Supplementary Table 4. Vectors used in this study.

Vectors/Plasmids	Characteristic(s)	Source/Reference
pSE101	Amp^r and Tsr^r, gene expression in *S.albus* G153	(1)
pSEamcB-H	Using for heterologous production of cispentacin	This study
pSEamcF-G	pSE101 derived plasmid for expressing *N*-terminal His-tagged AmcF and AmcG	This study
pSEamcH	pSE101 derived plasmid for expressing *N*-terminal His-tagged AmcH	This study
pKU503_ampr	Using as gene template	(2)
pET26(+)	Kan^r, protein expression vector	Novagen
pET26(+)-amcB	pET26(+) derived plasmid for expressing *C*-terminal His-tagged AmcB	This study
pET26(+)-sFabI	pET26(+) derived plasmid for expressing *C*-terminal His-tagged sFabI	This study
pET26(+)-eFabI	pET26(+) derived plasmid for expressing *C*-terminal His-tagged eFabI	This study
pHis8	Kan^r, protein expression vector in *E. coli* BL21(DE3)	(3)
pHis8-amcC	pHis8 derived plasmid for expressing *N*-terminal His-tagged AmcC	This study
pHis8-amcD	pHis8 derived plasmid for expressing *N*-terminal His-tagged AmcD	This study
pHis8-amcE	pHis8 derived plasmid for expressing N-terminal His-tagged AmcE	This study

Supplementary Table 5. The characterization of chemical and biomolecular materials.

Compound	Source	Purity	Identity
(1R,2S)-(−)-2-Amino-1-cyclopentanecarboxylic acid hydrochloride hemihydrate (Cispentacin)	Sigma‑Aldrich	98.0-102.0% (calc. on dry substance, AT)	712205
**Detection and quantification of cispentacin**			
1-fluoro-2,4-dinitrobenzene (DNFB)	NACALAI TESQUE, INC.	≧99.0%(GC)	70-34-8
**Synthesis of 3-oxocyclopent-1-ene-1,2-dicarboxylic acid**			
tert-butyl 2-(triphenylphosphoranylidene)acetate	Sigma‑Aldrich	98%	369799
3-chloropropionyl chloride	Tokyo Chemical Industry Co., Ltd. (TCI)	>98.0%(T)	625-36-5
OXONE®, monopersulfate compound	Sigma‑Aldrich	n/a	228036
2,3-di(carbo-tert-butoxy)-2-cyclopentenone	In this study	>90.0% (by 1H-NMR)	
3-oxocyclopent-1-ene-1,2-dicarboxylic acid	In this study	>90.0% (by 1H-NMR)	
**Marfey's Method**			
N α -(5-Fluoro-2,4-dinitrophenyl)-L-leucinamide (L-FDLA)	Tokyo Chemical Industry Co., Ltd. (TCI)	>98.0%(HPLC)	178065-29-7
N α -(5-Fluoro-2,4-dinitrophenyl)-D-leucinamide (D-FDLA)	Tokyo Chemical Industry Co., Ltd. (TCI)	>98.0%(HPLC)	178065-30-0

Supplementary Figures

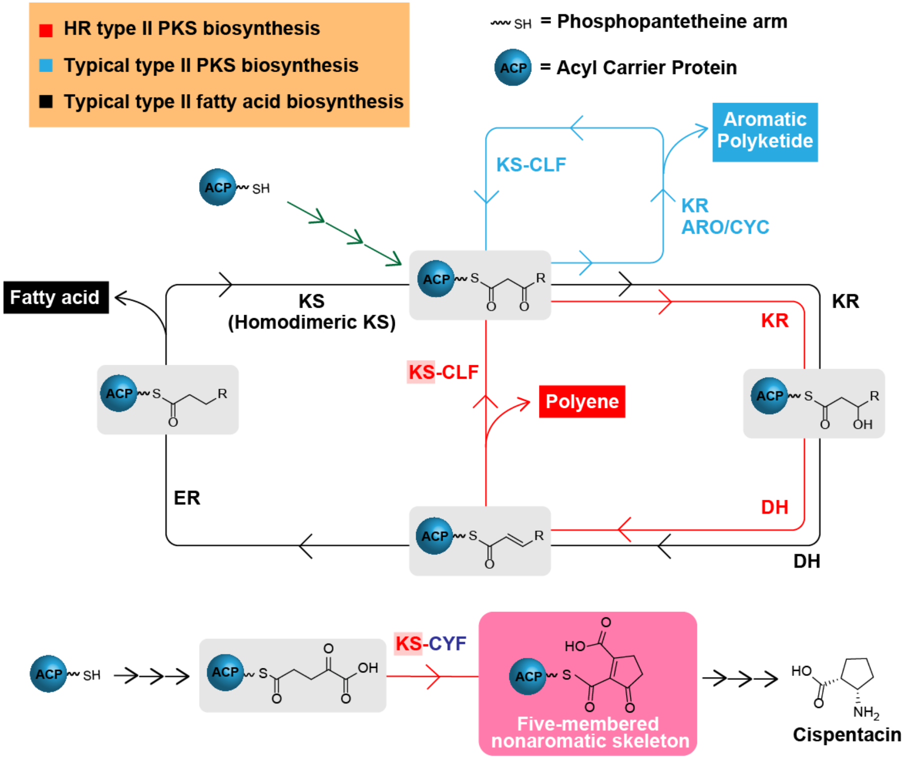

Supplementary Figure 1. Comparison of type II PKS subfamilies. Biosynthetic pathways involving highly reducing (HR) type II PKS (red arrows), typical type II PKS (blue arrows), type II fatty acid (black arrows), and the phylogenetically new subfamily of type II PKS (bottom) identified in this study. Abbreviations: ARO/CYC, aromatase/cyclase; CLF, chain length factor; DH, dehydratase; ER, enoyl-acyl carrier protein reductase; KR, ketoreductase; KS, ketosynthase; CYF, cyclization factor.

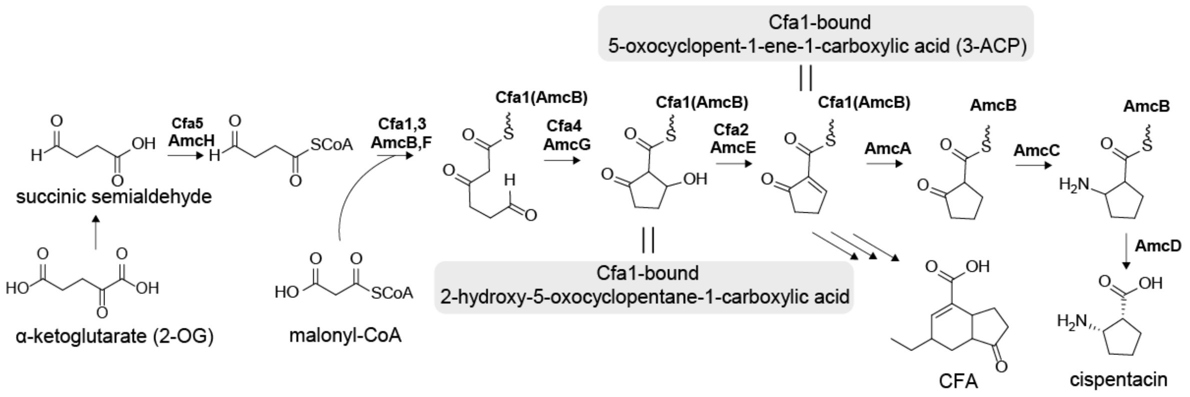

Supplementary Figure 2. Biosynthetic pathway of CPC-ACP postulated in previous studies. The postulated pathway begins with the conversion of succinic semialdehyde derived from 2-oxoglutarate into its CoA ester by Cfa5. Cfa1 (ACP), Cfa3 (KS), and Cfa4 are postulated to produce Cfa1-bound 2-hydroxy-5-oxocyclopentane-1-carboxylic acid, which is subsequently dehydrated by Cfa2 (DH) to give Cfa1-bound 5-oxocyclopent-1-ene-1-carboxylic acid. AmcB, E, F, G, and H show high similarity to those of Cfa1, 2, 3, 4, and 5, respectively, which suggests that the biosynthesis of CFA and cispentacin share a common route leading to 5-oxocyclopent-1-ene-1-carboxylic acid bound to either Cfa1 or AmcB.

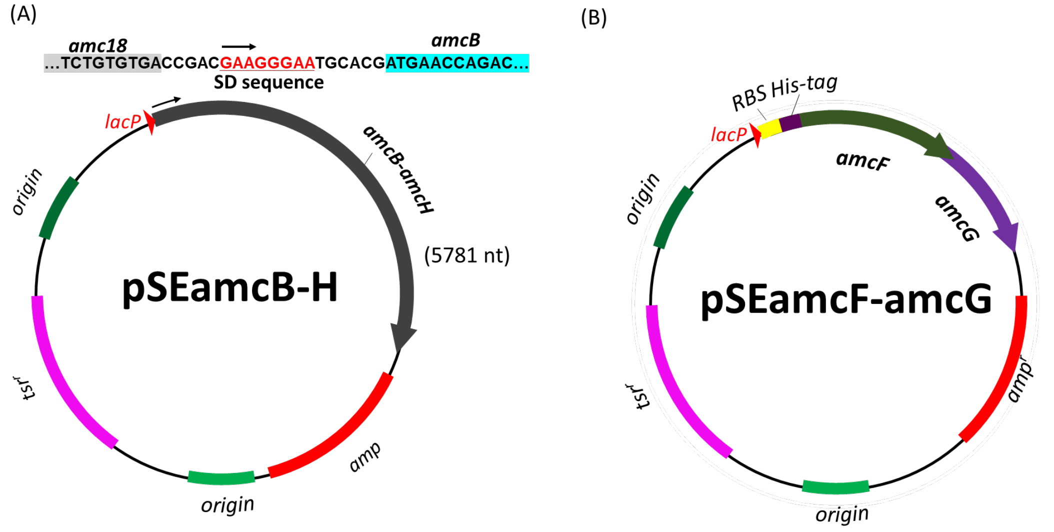

Supplementary Figure 3. Schematic representation of the plasmid constructs. (A) pSEamcB-H expresses the cispentacin biosynthetic operon (*amcB* to *amcH*) containing the SD sequence for translation initiation upstream of *amcB*. The operon is inserted downstream of the *lac* promoter (*lacP*). We have reported previously that genes inserted downstream of *lacP* demonstrate robust expression in *S. albus* (4). (B) pSEamcF-amcG coexpresses the operon of *amcF* and *amcG* (KS-CYF) in *S. albus* G153. The operon is inserted downstream of the *lac* promoter (*lacP*).

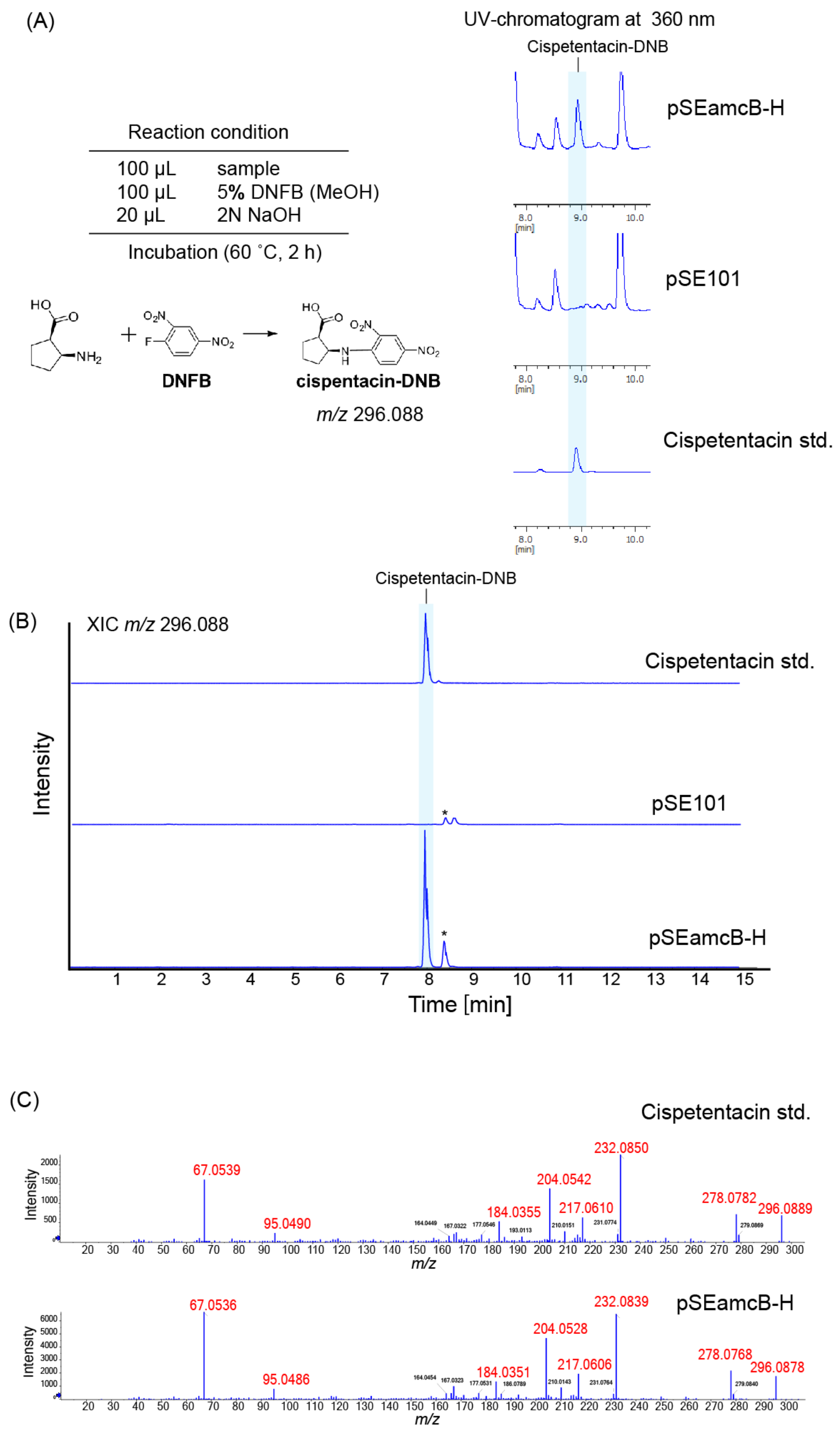
(A)
UV-chromatogram at 360 nm
Cispetentacin-DNB
pSEamcB-H
pSE101
Cispetentacin std.
Reaction condition
100 µL sample
100 µL 5% DNFB (MeOH)
20 µL 2N NaOH
Incubation (60 ˚C, 2 h)
DNFB
cispentacin-DNB
m/z 296.088
(B)
XIC m/z 296.088
Cispetentacin-DNB
Cispetentacin std.
pSE101
pSEamcB-H
Intensity
Time [min]
(C)
Cispetentacin std.
67.0539
95.0490
184.0355
204.0542
217.0610
232.0850
278.0782
296.0889
pSEamcB-H
67.0536
95.0486
184.0351
204.0528
217.0606
232.0839
278.0768
296.0878
Intensity
m/z

Supplementary Figure 4. X-LC and MS analysis of DNFB-derivatized cispentacin. (A) Reaction conditions for DNFB derivatization and chromatograms at 360 nm of the DNFB-derivatized culture broths from *S. albus* G153/pSE101 and *S. albus* G153/pSEamcB-H. (B) Extracted ion count (XIC) chromatograms at *m/z* 296.088 corresponding to the DNFB-derivatized cispentacin in the broths from *S. albus* G153/pSE101 and *S. albus* G153/pSEamcB-H. (C) MS/MS spectrum of the DNFB-derivatized cispentacin. The spectrum from the culture broth of *S. albus* G153/pSEamcB-H matches well with that of the authentic DNFB-derivatized cispentacin.

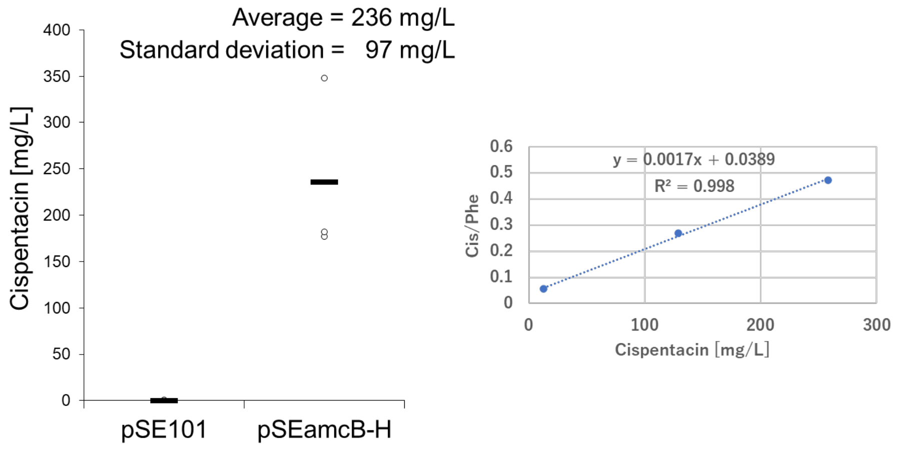

Supplementary Figure 5. Production of cispentacin by *S. albus* G153/pSEamcB-H. *S. albus* G153/pSE101 was used as a negative control transformant. Dots correspond to cispentacin production in individual cultures; the bars correspond to average values. Average and standard deviation values were calculated with *S. albus* G153 samples (n = 3, independent culture samples). Source data are provided as a Source Data file.

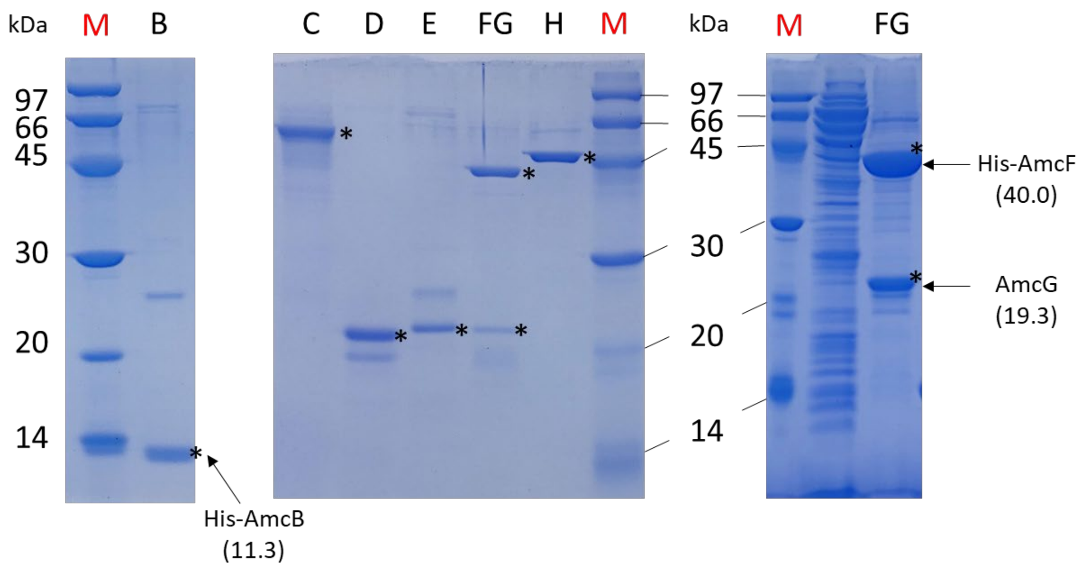

Supplementary Figure 6. SDS–PAGE analysis of purified recombinant proteins. SDS–PAGE (12%) analysis of recombinant proteins. Lane B, AmcB, calculated MW 11.3 kDa; Lane C, AmcC, calculated MW 51.1 kDa; Lane D, AmcD, calculated MW 16.7 kDa; Lane E, AmcE, calculated MW 18.8 kDa; Lane FG, AmcF–AmcG, calculated MWs 40.0 kDa for AmcF and MWs 19.3 kDa for AmcG; Lane H, AmcH, calculated MW 49.6 kDa. All these proteins except AmcG were produced as His-tagged proteins (marked with an asterisk) and purified by affinity chromatography using a Ni-NTA Superflow resin. Lane M is a molecular weight marker. In Lane B, a small amount of phosphopantetheinyl transferase (25 kDa), which is coexpressed in *E. coli* cells for phosphopantetheinyl transfer of AmcB, was also detected. In Lanes C to H, each target protein marked with an asterisk is major, although unknown trace proteins were detected. Source data are provided as a Source Data file.

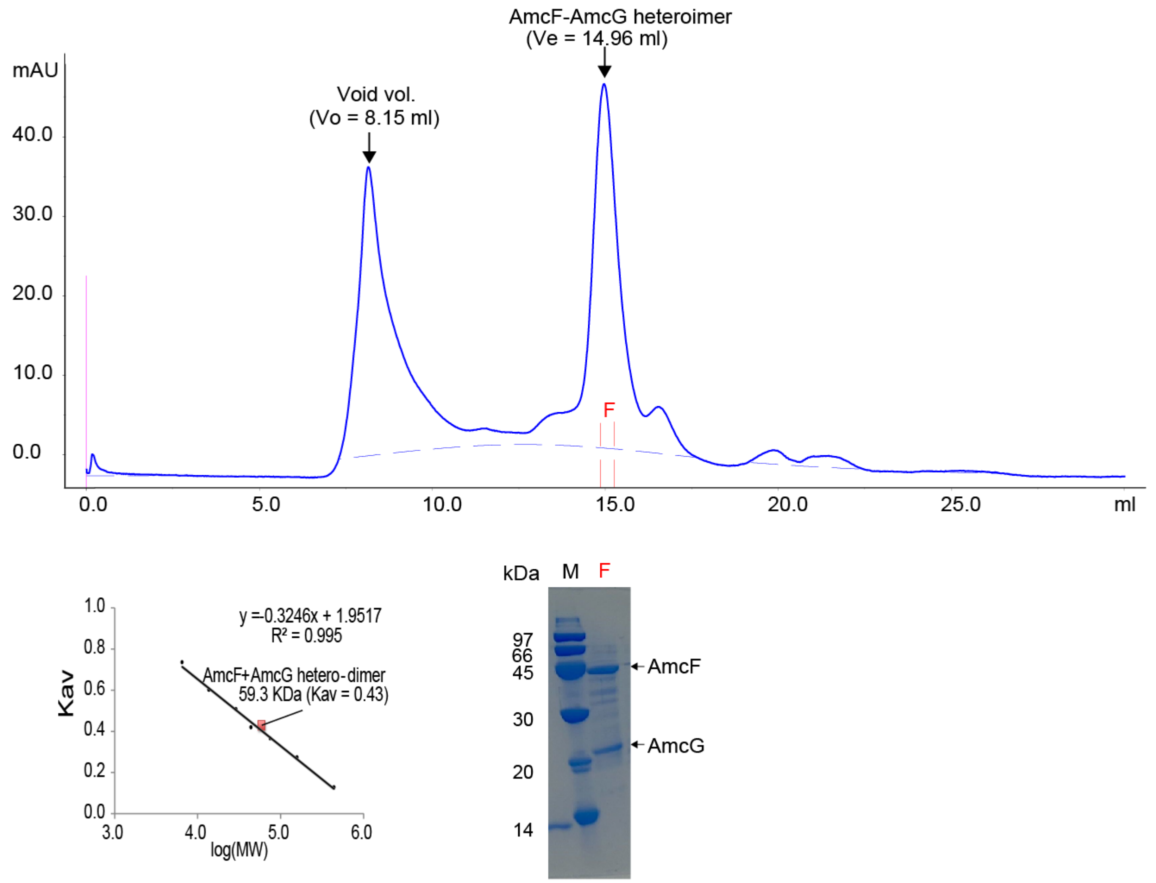

Supplementary Figure 7. Gel filtration of AmcF-AmcG. Gel filtration on a Superdex 200 Increase 10/300 GL column. AmcF-AmcG (1.5 mg) was subjected to the column. A single peak corresponding to the mass of the AmcF-AmcG heterodimer was detected. Lane F, SDS–PAGE (12%) analysis of the purified AmcF-AmcG heterodimer fraction. **K*av = (*V*e − *V*o)/(*V*c − *V*o), *V*c (geometric column volume) = 24 (mL), *V*o (column void volume). Source data are provided as a Source Data file.

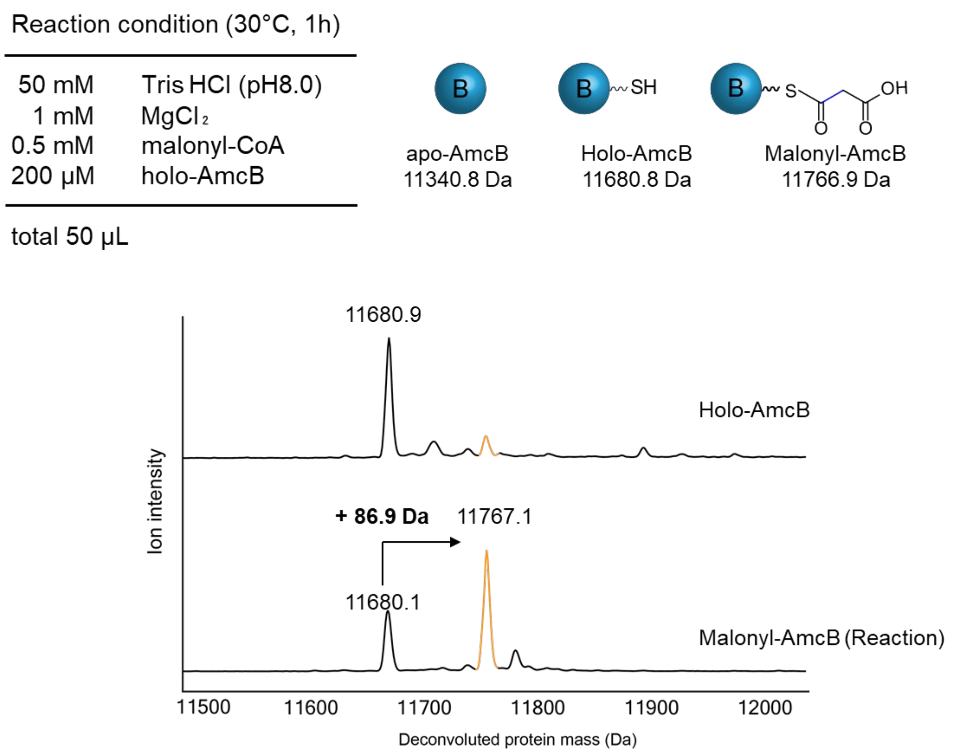

Supplementary Figure 8. Detection of AmcB bound intermediate derived from malonyl-CoA. Deconvoluted protein mass for *holo*-AmcB was detected at 11680.9 Da. The deconvoluted protein mass for malonyl-AmcB (orange line) was detected at 11767.1 Da under the reaction conditions shown. A small amount of malonyl-AmcB that formed during AmcB production in *E. coli* was detected in the purified sample of *holo*-AmcB.

Reaction condition (30°C, 1h)	
1 mM	Substrate
5 mM	ATP
50 mM	Tris HCl (pH8)
25 mM	$MgCl_2$
200 μM	AmcB
2 μM	AmcH
total 50 μL	

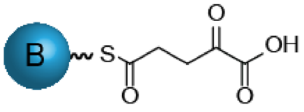

1-AmcB
11808.8 Da

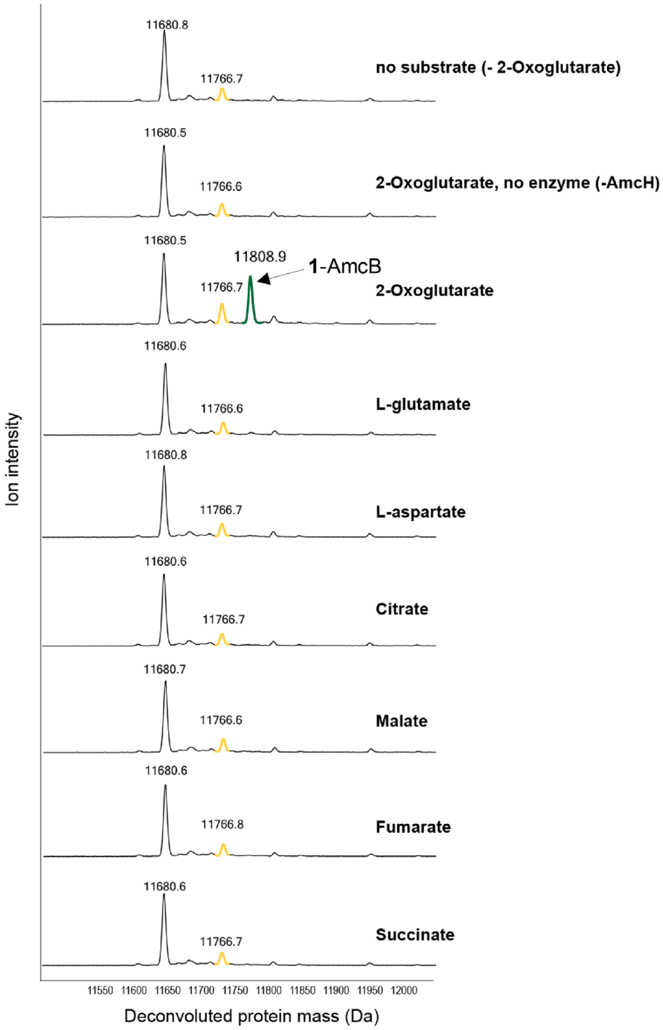

Supplementary Figure 9. Substrate specificity of AmcH. Spectra for deconvoluted protein mass of **1**-AmcB, which was detected at 11808.9 Da. Substrate-tethered AmcB was formed only when 2-oxoglutarate was used as a substrate for AmcH.

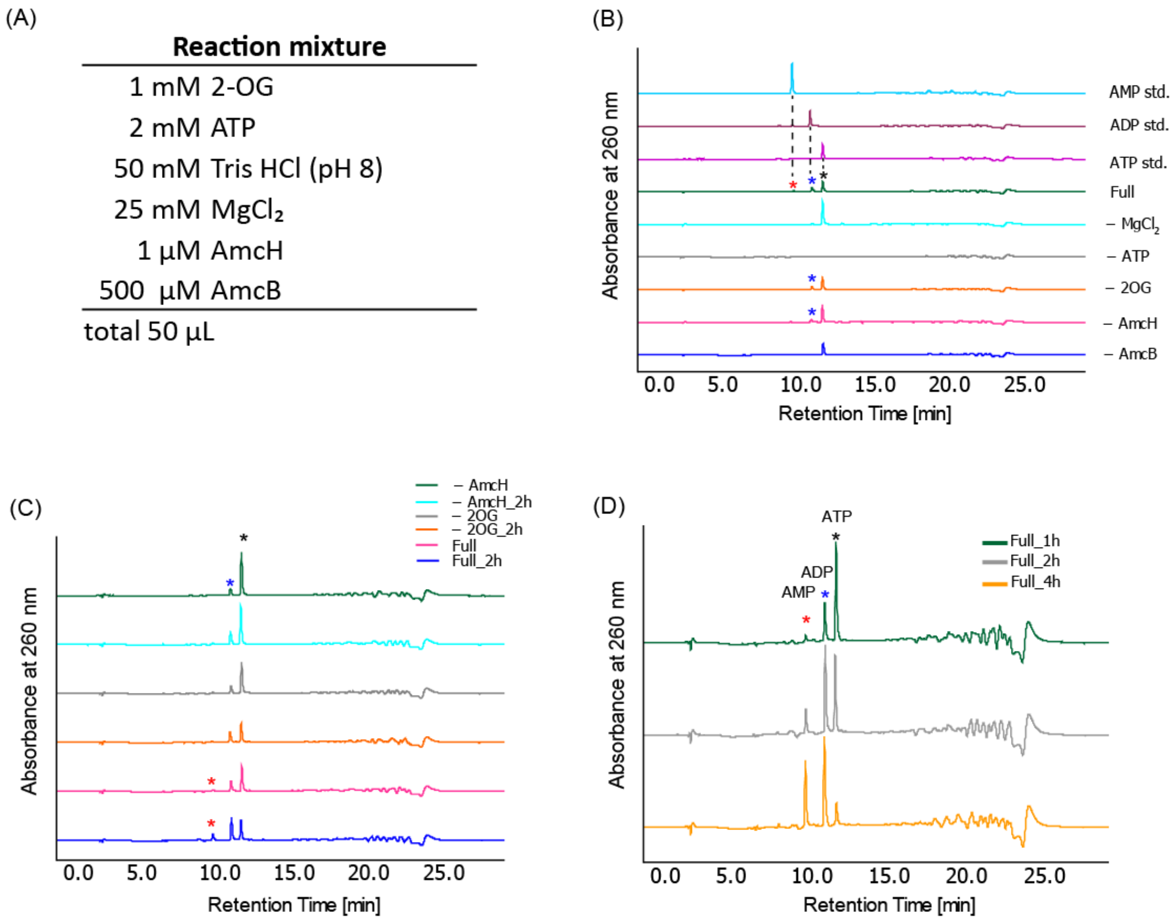

Supplementary Figure 10. HPLC analysis of AmcH activity. The reaction conditions are described in the Experimental Section. (A) Composition of the reaction mixture. (B) Each AmcH reaction was conducted for 1 h. Conversion of ATP to ADP and AMP was detected in the sample of Full. (C) Conversion of ATP to ADP was detected in the sample including AmcB. (D) The AmcH reactions were conducted for 1, 2, and 4 h. Almost all of the ATP was converted to AMP or ADP during the 4 h reaction. ADP probably formed due to spontaneous hydrolysis or hydrolysis by residual phosphatase from *E. coli*.

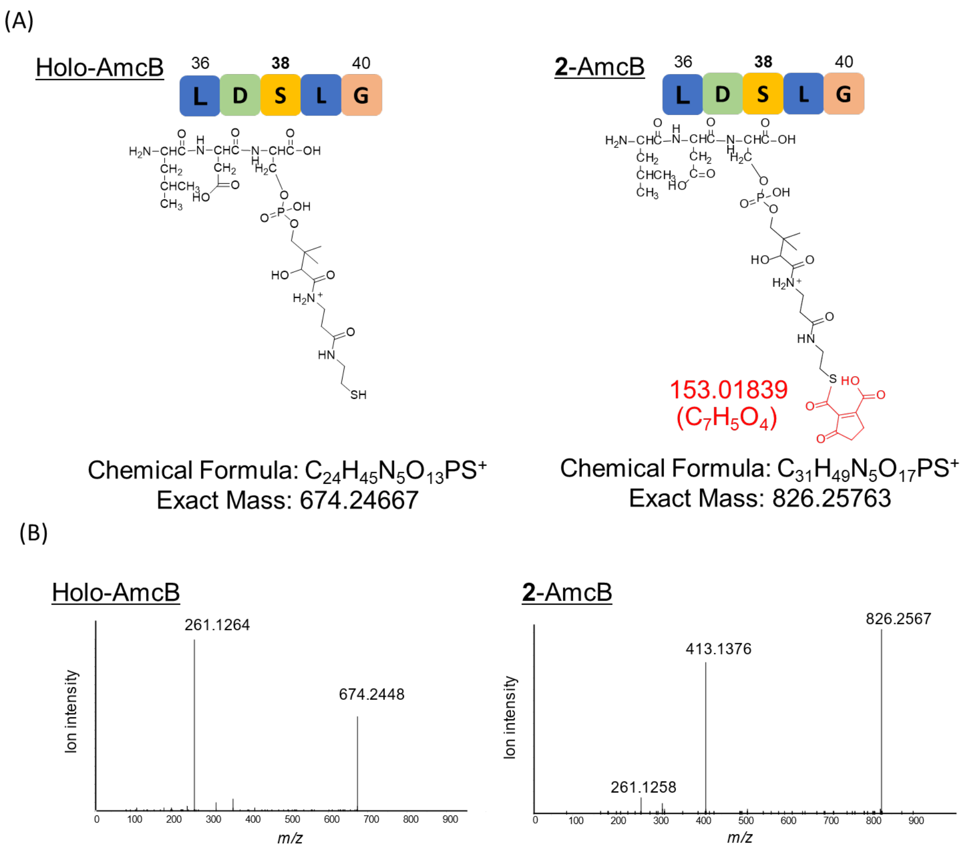

Supplementary Figure 11. LC–ESI–HRMS analysis of 2-AmcB digested with thermolysin. (A) Schematic representation of thermolysin-digested peptide fragments of *holo*-AmcB and **2**-AmcB. (B) Extracted ion chromatograms are shown for the digested peptide fragments of *holo*-AmcB (*m/z* 674.2448 $[M+H]^+$) and **2**-AmcB (*m/z* 826.2567 $[M+H]^+$).

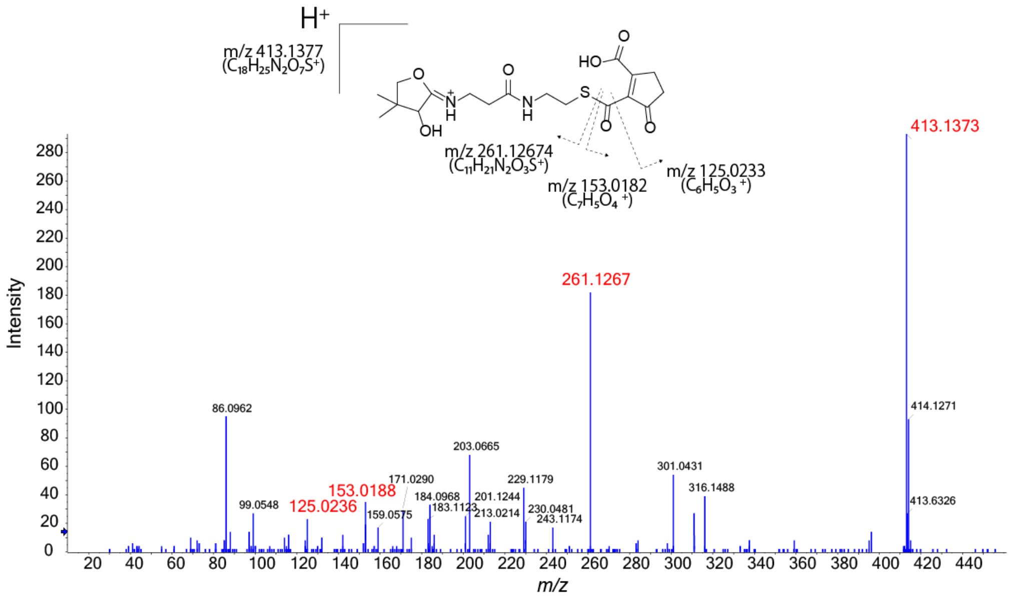

Supplementary Figure 12. MS/MS spectrum of the PPant-eliminated ion of 2-AmcB.

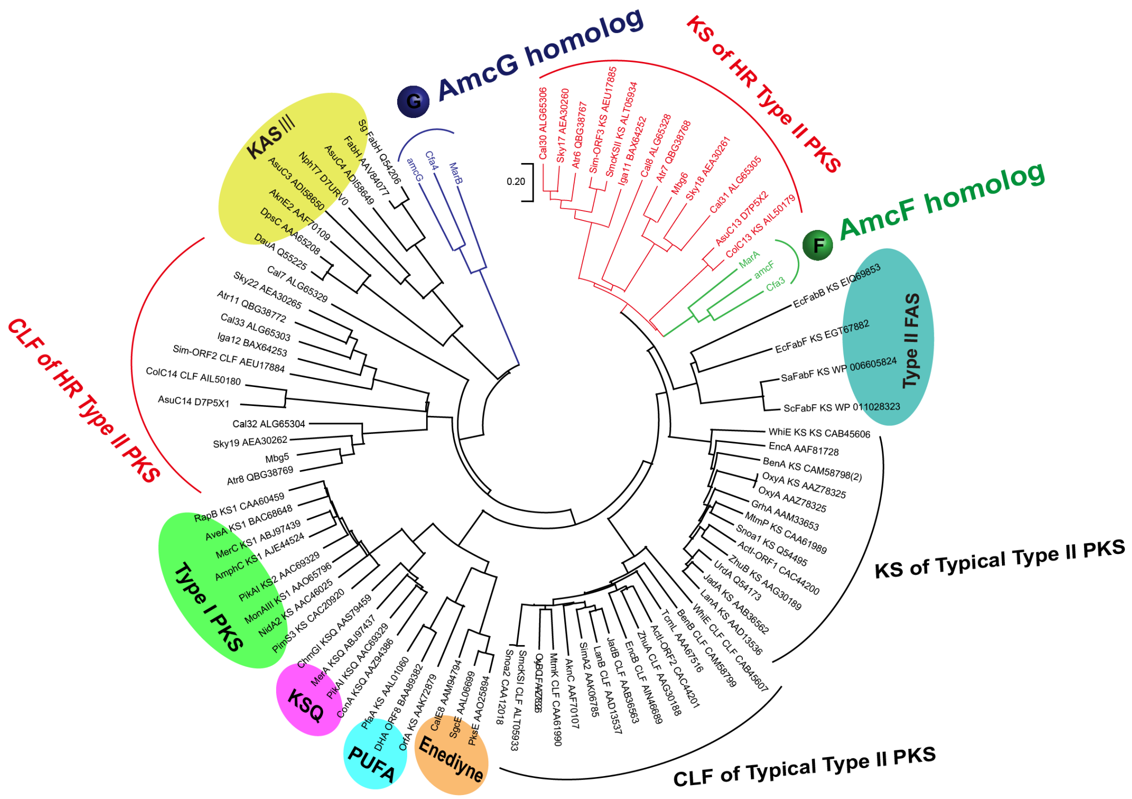

Supplementary Figure 13. Phylogenetic analysis of KSs in the cispentacin cluster and various types of known KSs. KSs in the cispentacin cluster, CFA cluster, and MAR cluster (AmcF homologs colored in green) are classified into the same clade of highly reduced (HR) type II PKSs [5]. The complex partners Cfa4, MarB, and AmcG (AmcG homologs colored in navy blue) that form a heterodimer with Cfa3, MarA, and AmcF, respectively, are also classified into the same clade, but they do not belong to any other KS clade and seem to form a new clade. The accession numbers of each protein are shown in square brackets. The phylogenetic tree was generated using the neighbor-joining method in MEGA X [6].

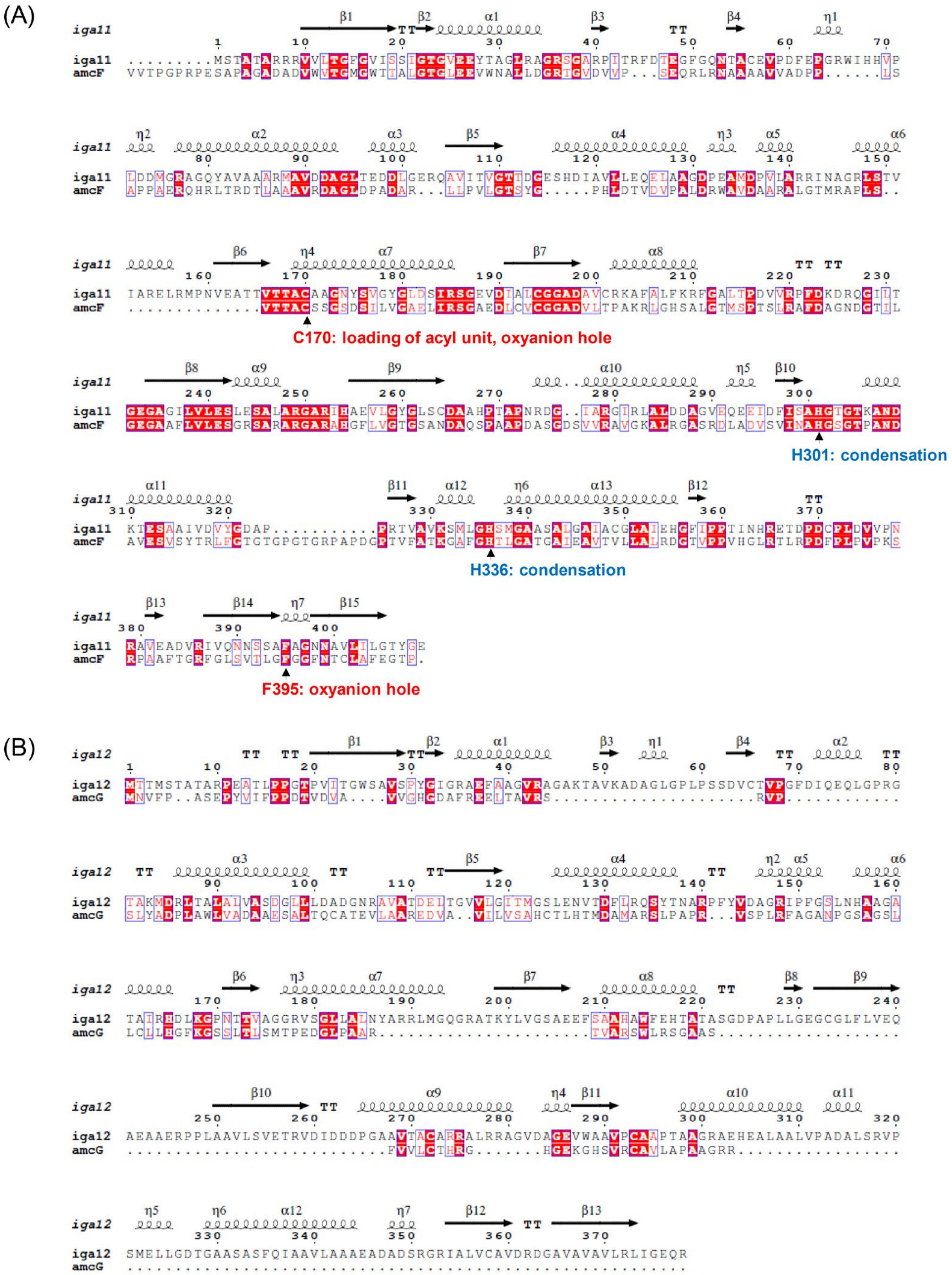

Supplementary Figure 14. Sequence analysis of AmcF and AmcG. (A) Alignment of AmcF with HR type II PKS Iga11 (KS). The secondary structure is derived from the Iga11 structure (6KXD_chainA) and indicated on the corresponding Iga11 sequence. Four catalytic residues (C170 and P395 for oxyanion hole, H301 and H336 for condensation) are highly conserved. (B) Alignment of AmcG with HR type II PKS Iga12 (CLF). The secondary structure is derived from the Iga11 structure (6KXD_chainB) and indicated on the corresponding Iga12 sequence. AmcG shows almost no sequence similarity to Iga12. The AmcG sequence is much shorter than the Iga12 sequence.

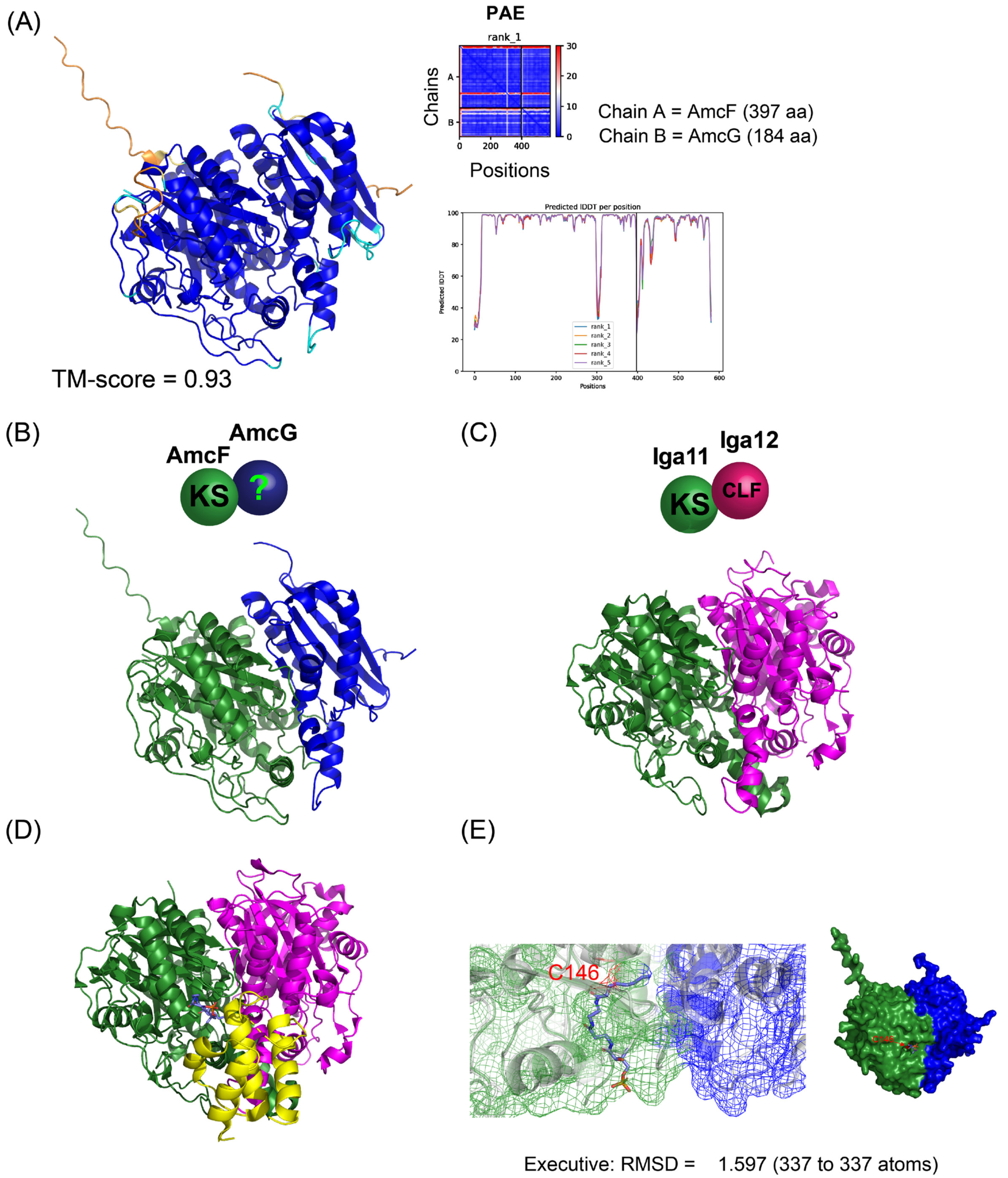

Supplementary Figure 15. AmcF–AmcG heterodimer fold prediction. (A) The predicted structure of the heterodimer AmcF–AmcG obtained by ColabFold. b=pLDDT (blue, b < 100), (cyan, b < 90), (yellow–orange, b < 70), (color tv_orange, b < 50). Predicted aligned error (PAE) and pLDDT (Rank_1 corresponds to the structure of the description) are shown as confidence measures. (B) Color-coded predicted structure of AmcF–AmcG (green, AmcF), (blue, AmcG). (C) The structure of the KS–CLF heterodimer (6KXD) (green, Iga11 (KS)), (pink, Iga12 (CLF)). (D) The structure of ACP=KS–CLF (6KXF) used to predict the ligand binding site of AmcF–AmcG

(yellow, Iga10 (ACP)). (E) Ligand binding cavity and predicted catalytic residue (green, AmcF), (blue, AmcG), (white, Iga11–12), (yellow, the substrate of Iga11–12). The RMSD value between AmcF–AmcG and Iga11–Iga12 is also shown.

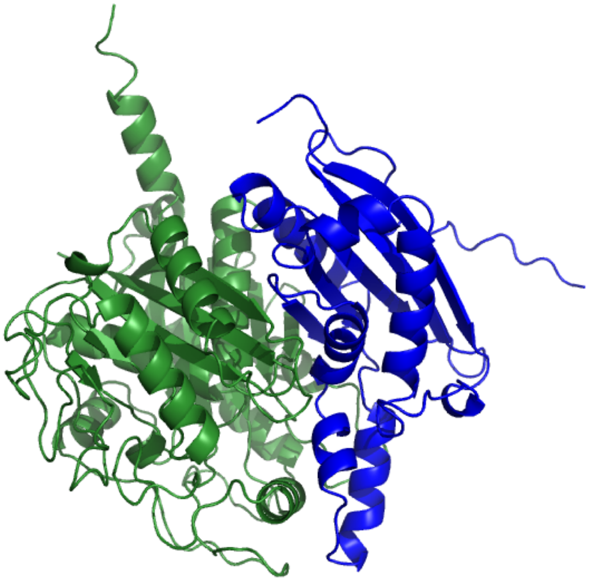

pDockQ = 0.71

num_contacts	avg_if_plddt	pdockq
179	93.11050324	0.706735165

Supplementary Figure 16. Assessment of the dimer structure. The predicted structure of the heterodimer AmcF–AmcG and the score of the predicted DockQ score (pDockQ) were obtained by SpeedPPI.

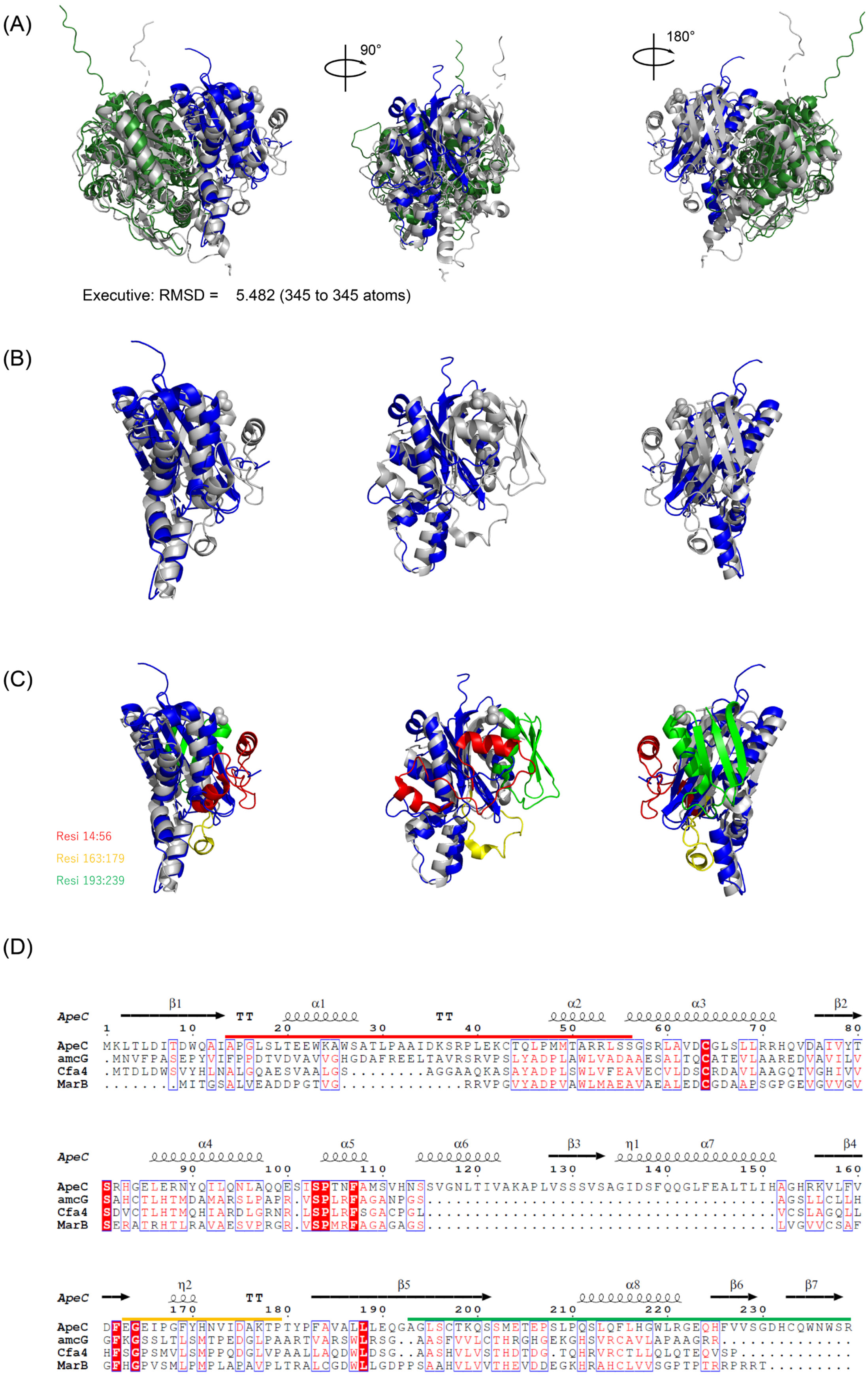
(A)
90°
180°
Executive: RMSD = 5.482 (345 to 345 atoms)
(B)
(C)
Resi 14:56
Resi 163:179
Resi 193:239
(D)
ApeC β1 TT α1 TT α2 α3 β2
1 10 20 30 40 50 60 70 80
ApeC MKLTLDITDWQAIAPGLSLTEEWKAWSATLPAAIDKSRPLEKCTQLPMMTARRLSSGSRLAVDCGLSLLRRHQVDAIVYT
amcG .MNVFPASEPYVIFPPDTVDVAVVGHGDAFREELTAVRSRVPSLYADPLAWLVADAAESALTQCATEVLAAREDVAVILV
Cfa4 .MTDLDWSVYHLNALGQAESVAALGS.........AGGAAQKASAYADPLSWLVFEAVECVLDSCRDAVLAAGQTVGHIVV
MarB ........MITGSALVEADDPGTVG.............RRVPGVYADPVAWLMAEAVAEALEDCGDAAPSGPGEVGVVGV
ApeC α4 α5 α6 β3 η1 α7 β4
90 100 110 120 130 140 150 160
ApeC SRHGELERNYQILQNLAQQESISPTNFAMSVHNSSVGNLTIVAKAPLVSSSVSAGIDSFQQGLFEALTLIHAGHRKVLFV
amcG SAHCTLHTMDAMARSLPAPR.VSPLRFAGANPGS.............................AGSLLCLLH
Cfa4 SDVCTLHTMQHIARDLGRNR.LSPLRFSGACPGL.............................VCSLAGQLL
MarB SERATRHTLRAVAESVPRGR.VSPMRFAGAGAGS.............................LVGVVCSAF
ApeC η2 TT β5 α8 β6 β7
170 180 190 200 210 220 230
ApeC DFEGEIPGFYHNVIDAKTPTYPFAVALLLEQGAGLSCTKQSSMETEPSLPQSLQFLHGWLRGEQHFVVSGDHCQWNWSR
amcG GFKGSSLTLSMTPEDGLPAARTVARSWLRSG..AASFVVLCTHRGHGEKGHSVRCAVLAPAAGRR..............
Cfa4 HFSGPSMVLSMPPQDGLVPAALLAQDWLDSG..AASHVLVSTHDTDG.TQHRVRCTLLQLQTEQVSP............
MarB GFHGPVSMLPMPLAPAVPLTRALCGDWLLGDPPSAAHVLVVTHEVDDEGKHRAHCLVVSGPTPTRRPRRT.........

Supplementary Figure 17. Comparative analysis of CLF and cyclization factors (CYFs).
(A) Predicted structure of the heterodimer AmcF–AmcG (green, AmcF) and (blue, AmcG) compared with the structure of the KS–CLF heterodimer (6qsp) (gray, ApeO (KS) and ApeC (CLF)) from three angles. ApeC is the smallest reported structure in CLF, and the structure of AmcG (CYF) further lacks several secondary structures existing in ApeC (CLF). The RMSD value between AmcF–AmcG and ApeO–ApeC is also shown. (B) AmcG structures extracted from the heterodimeric structure compared with the ApeC structure from three angles. (C) Secondary structures of ApeC not present in AmcG (ApeC residues from 14 to 56, red) (ApeC residues from 163 to 179, orange) (ApeC residue from 193 to 239, green). (D) Alignment of AmcG homologs (Cfa4 and MarB) with HR type II PKS ApeC (CLF). The secondary structure is derived from the ApeC structure (6QSP_chainB) and indicated on the corresponding ApeC sequence. The three colored lines (red, orange, and green) correspond to the secondary structures of ApeC in (C). AmcG homologs show almost no sequence similarity to ApeC. This alignment shows no correlation between similar regions in structure and similar regions in alignment.

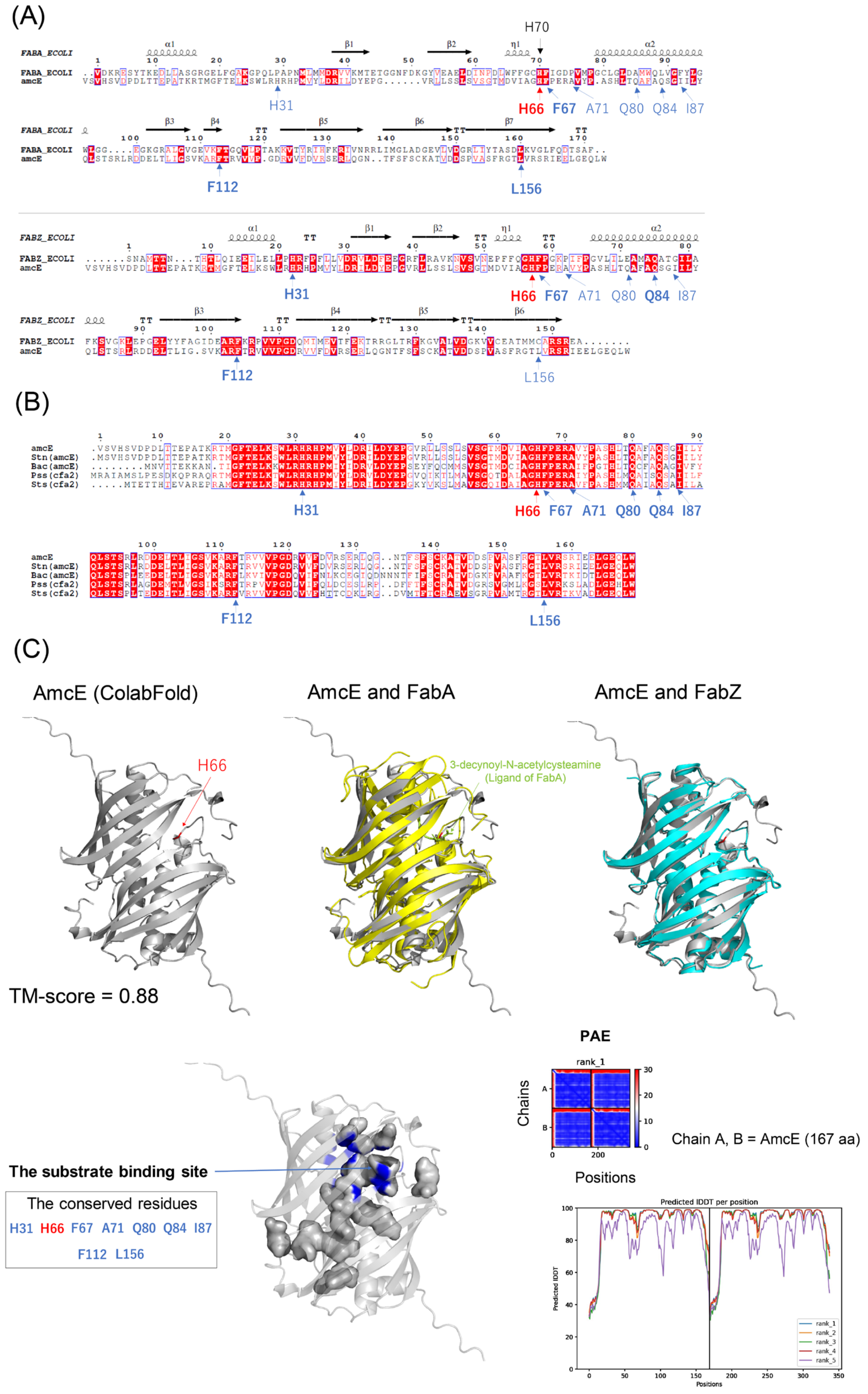

(A)
H70
H31
H66 F67 A71 Q80 Q84 I87
F112
L156
H31
H66 F67 A71 Q80 Q84 I87
F112
L156
(B)
H31
H66 F67 A71 Q80 Q84 I87
F112
L156
(C)
AmcE (ColabFold)
AmcE and FabA
AmcE and FabZ
H66
3-decynoyl-N-acetylcysteamine
(Ligand of FabA)
TM-score = 0.88
PAE
Chains
Positions
Chain A, B = AmcE (167 aa)
Predicted lDDT per position
The substrate binding site
The conserved residues
H31 H66 F67 A71 Q80 Q84 I87
F112 L156

Supplementary Figure 18. Sequence analysis of AmcE. (A) Sequence alignments of AmcE with FabA (P0A6Q3) and AmcE with FabZ (P0A6Q6). The predicted catalytic residue histidine 66 (H66) is conserved. (B) Alignment of AmcE homologs in the cispentacin and CFA gene cluster. Stn, AmcE homolog of *Streptomyces novoguineensis* QHW08541.1; Bac, AmcE homolog of *Bacillus cereus* WP_098276130.1; Pss, Cfa2 homolog of *Pseudomonas syringae* WP_248842617.1; Sts, Cfa2 homolog of *Streptomyces scabies* WP_013005371.1. The residues of the active site (blue and red) are highly conserved in the AmcE homologs in contrast to FabZ. (C) The predicted structure of the homodimer of AmcE obtained by ColabFold (gray). The overall structure is similar to the crystal structure of FabA (1mka) and FabZ (6n3p) (yellow, FabA), (cyan, FabZ). The substrate binding sites and colored conserved residues are shown in the predicted structure of AmcE. Predicted aligned error (PAE) and pLDDT (Rank_1 corresponds to the structure of the description) are shown as confidence measures.

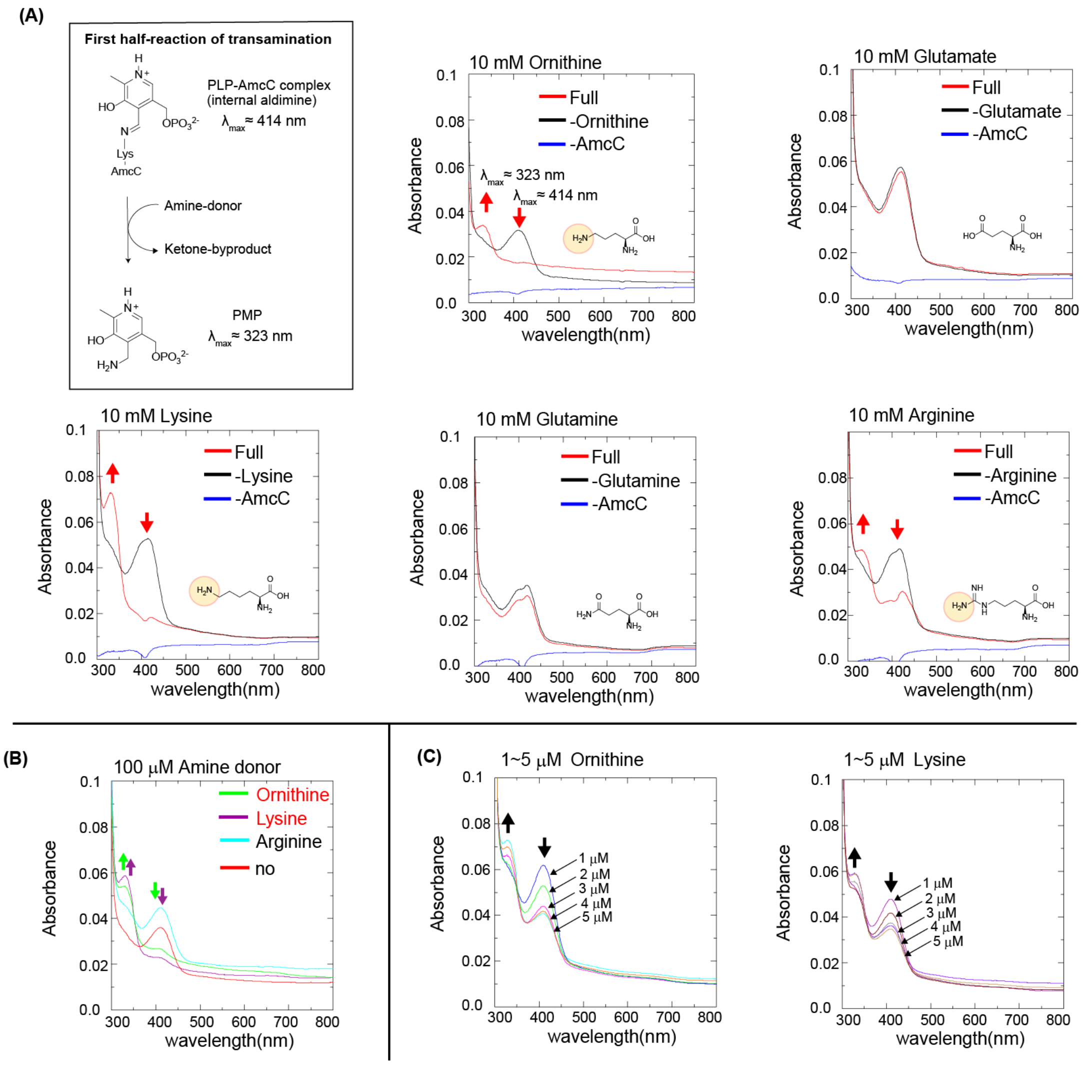

Supplementary Figure 19. UV–vis spectra assay for AmcC. (A) Analysis of the AmcC-catalyzed reaction with UV–vis spectra assay using 10 mM amine donor. The first half-reaction of transamination can be detected by monitoring the change in absorbance from 414 nm (PLP–AmcC complex) to 323 nm (PMP). A shift to 323 nm was detected when ornithine, lysine or arginine was used. (B) Analysis of the AmcC-catalyzed reaction with UV–vis spectra assay using 100 µM amine donor. A shift to 323 nm was detected when ornithine or lysine was used. (C) Analysis of the AmcC-catalyzed reaction with UV–vis spectra assay using 1–5 µM ornithine or lysine. A shift to 323 nm was detected in both cases.

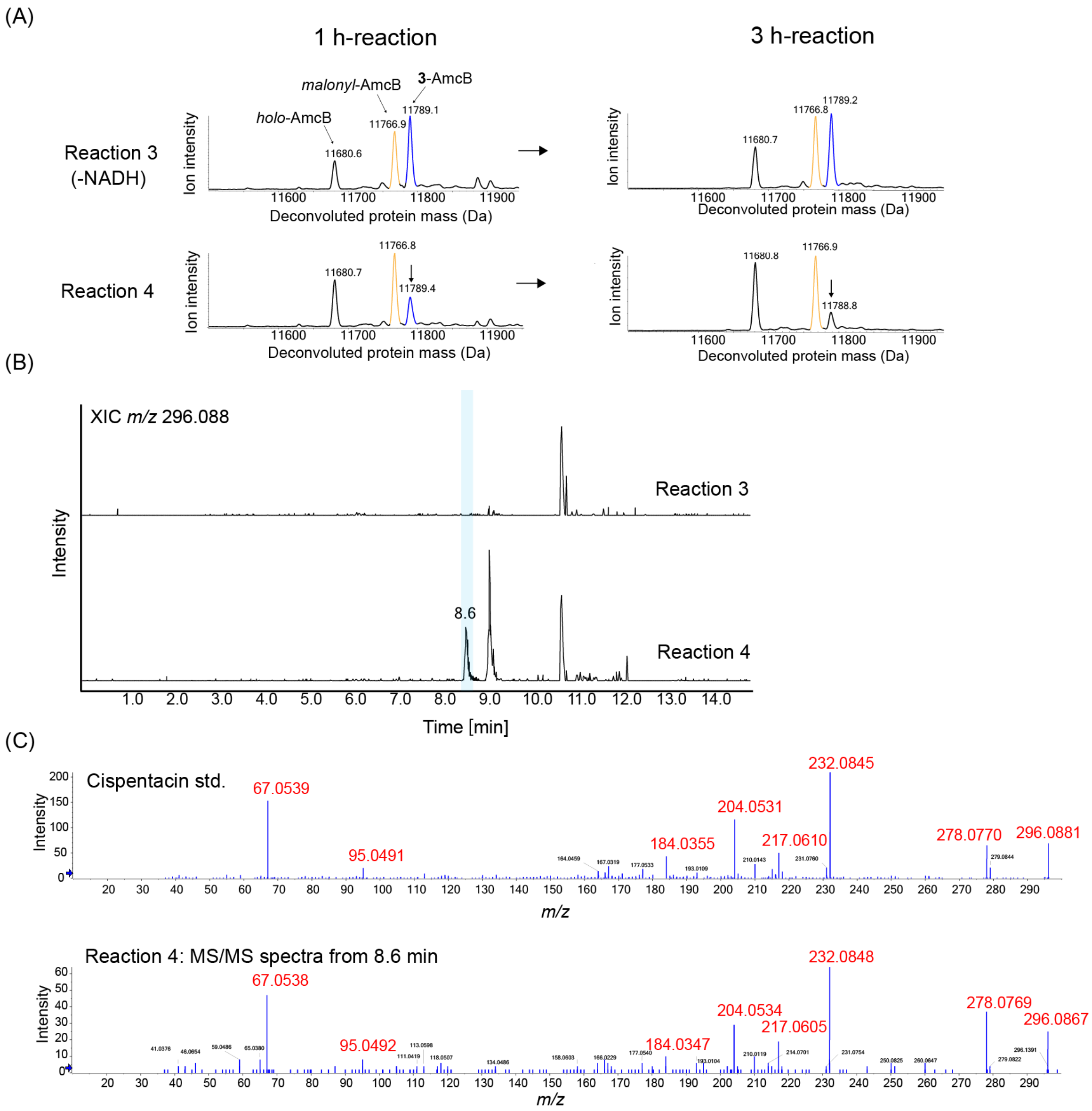

Supplementary Figure 20. LC–MS analysis of cispentacin in the reconstituted reaction mixture. (A) Deconvoluted protein mass spectra from Reactions 3 and 4. The deconvoluted protein mass signal for **3**-AmcB (11789 Da, blue) was significantly decreased in Reaction 4. (B) LC–MS analysis of DNFB-derivatized reaction mixtures. XIC chromatograms at *m/z* 296.088 in the derivatized Reactions 3 and 4. (C) MS/MS spectra of the DNFB-derivatized cispentacin. The spectrum from reaction mixture 4 well matches that of the DNFB-derivatized cispentacin.

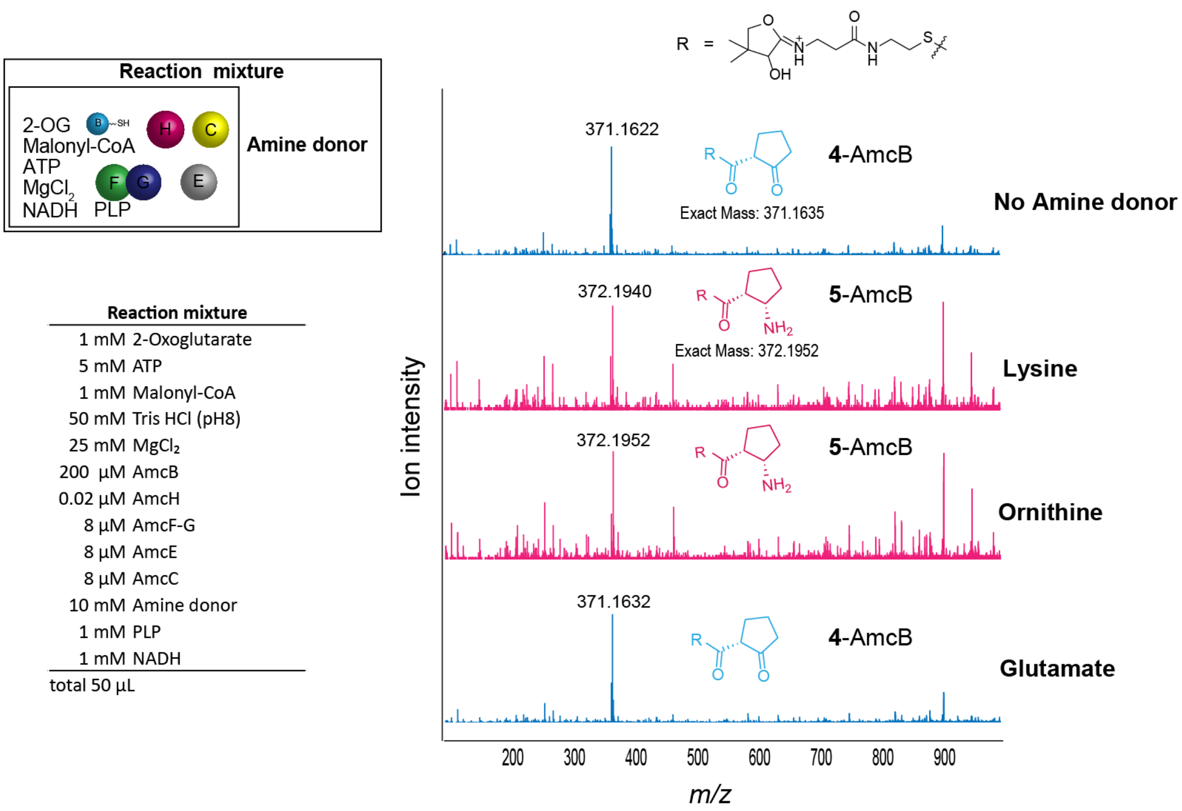

Supplementary Figure 21. Amine donor specificity of AmcC. PPant ejection assay was used for the amine donor specificity of AmcC. MS/MS fragments of the 13+ charge state of **4**-AmcB and **5**-AmcB were analyzed. The PPant-eliminated ion of **5**-AmcB (*m/z* 372.1952) was detected when lysine or ornithine was added as an amine donor.

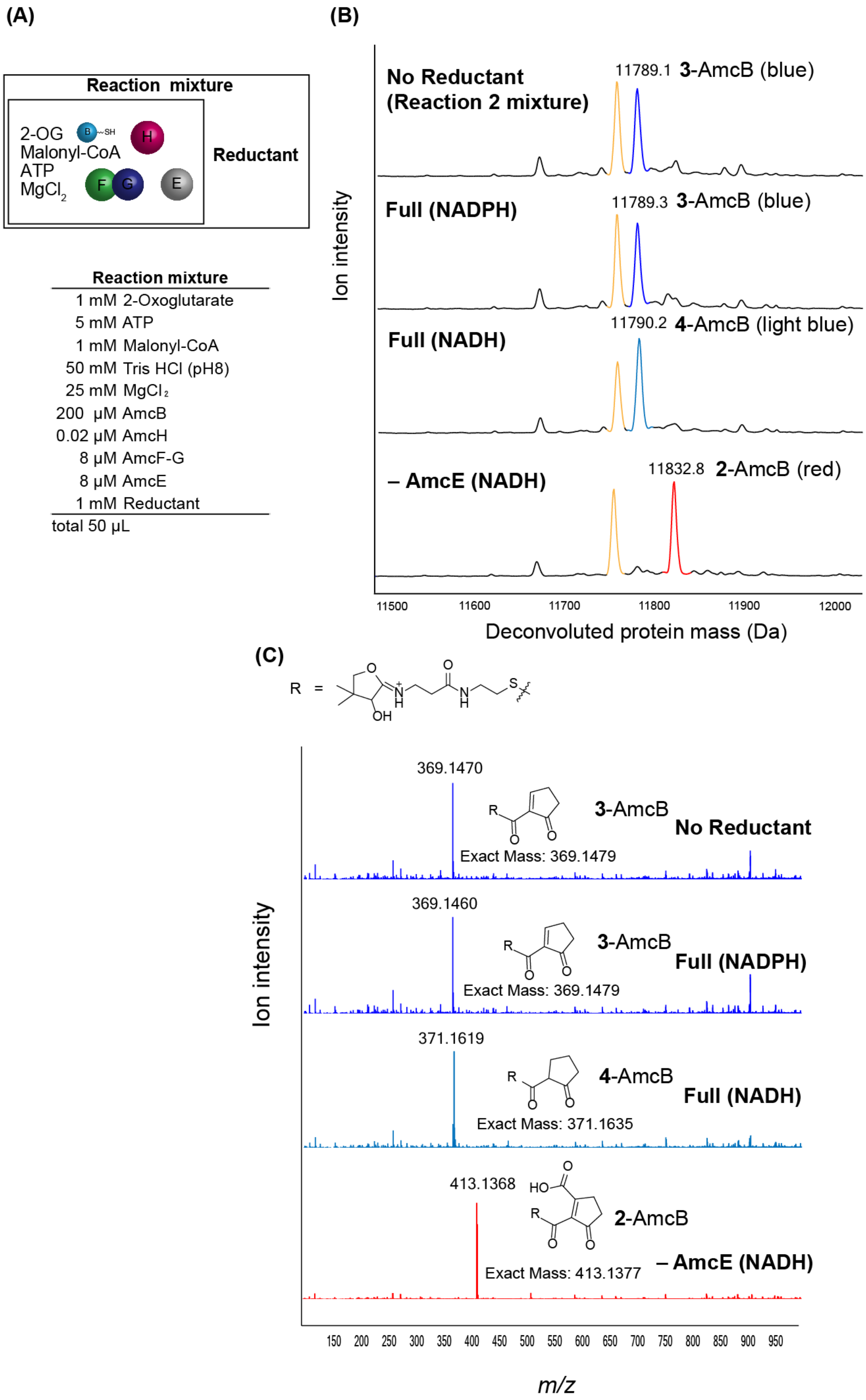

(A)
Reaction mixture
2-OG
Malonyl-CoA
ATP
$MgCl_2$
B ~SH
H
F G
E
Reductant
Reaction mixture
1 mM 2-Oxoglutarate
5 mM ATP
1 mM Malonyl-CoA
50 mM Tris HCl (pH8)
25 mM $MgCl_2$
200 μM AmcB
0.02 μM AmcH
8 μM AmcF-G
8 μM AmcE
1 mM Reductant
total 50 μL
(B)
Ion intensity
No Reductant (Reaction 2 mixture)
11789.1 3-AmcB (blue)
Full (NADPH)
11789.3 3-AmcB (blue)
Full (NADH)
11790.2 4-AmcB (light blue)
– AmcE (NADH)
11832.8 2-AmcB (red)
11500
11600
11700
11800
11900
12000
Deconvoluted protein mass (Da)
(C)
R =
O
N H
OH
S
Ion intensity
369.1470
3-AmcB
No Reductant
Exact Mass: 369.1479
369.1460
3-AmcB
Full (NADPH)
Exact Mass: 369.1479
371.1619
4-AmcB
Full (NADH)
Exact Mass: 371.1635
413.1368
HO
2-AmcB
– AmcE (NADH)
Exact Mass: 413.1377
150 200 250 300 350 400 450 500 550 600 650 700 750 800 850 900 950
m/z

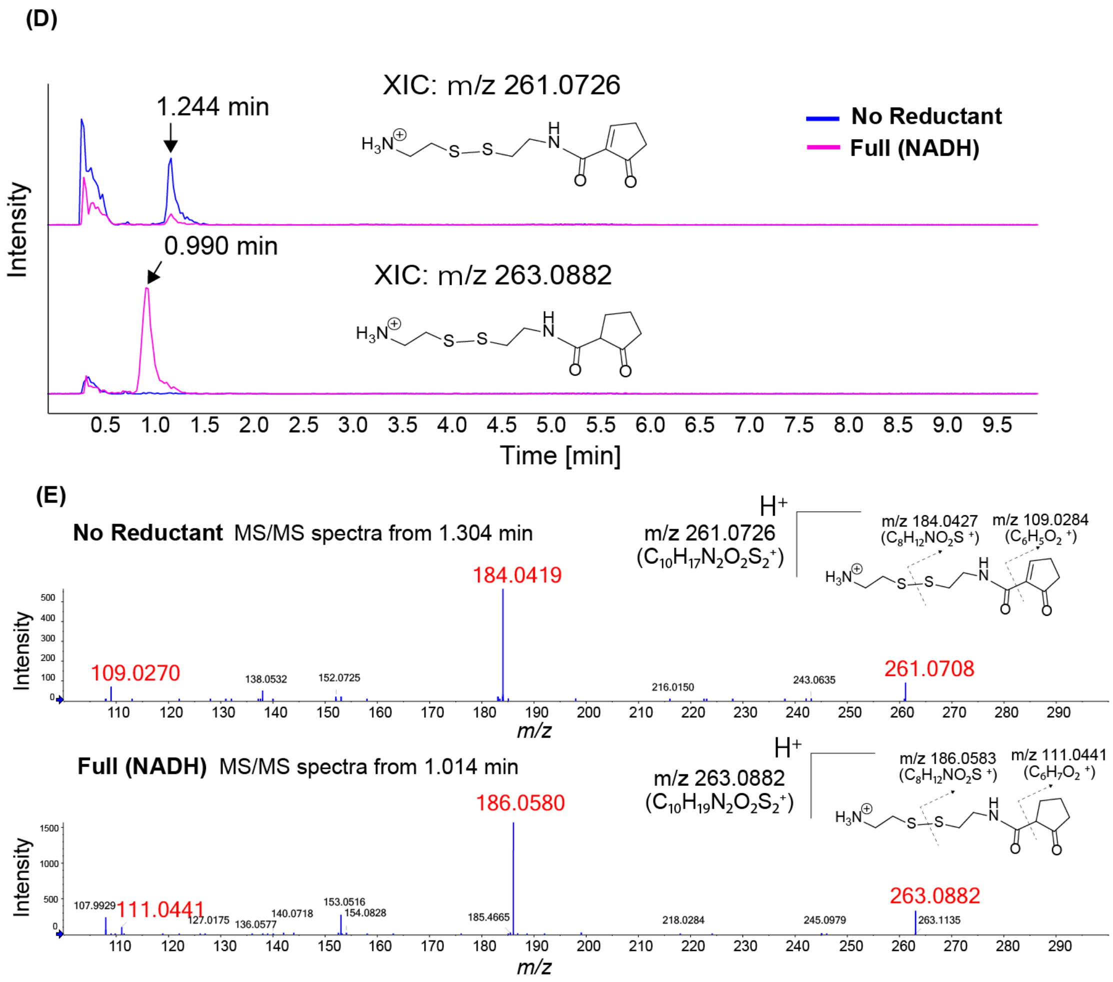

Supplementary Figure 22. NADH-dependent reduction of 3-AmcB. (A) The reaction conditions are based on Reaction 2. (B) Deconvoluted protein mass spectra for the reaction product under each condition. MS/MS fragments of the 13+ charge state of intermediate-AmcB were analyzed. The MS/MS fragment of **4**-AmcB was detected only in the reaction mixture (Full (NADH)). (C) The PPANT ejection assay for the reaction product under each condition. The PPant-eliminated ion of **4**-AmcB was detected only in the reaction mixture (Full (NADH)). (D) Cysteamine-promoted cleavage assay. Extracted ion count (XIC) chromatograms at *m/z* 261.0726 corresponding to the cysteamine adduct of **3** and *m/z* 263.0882 corresponding to the cysteamine adduct of **4** in the reaction mixture (No reductant) and (Full (NADH)), respectively. The formation of **4**-AmcB was confirmed in the reaction mixture (Full (NADH)). (E) MS spectra for the cysteamine adduct of **3** and the cysteamine adduct of **4**.

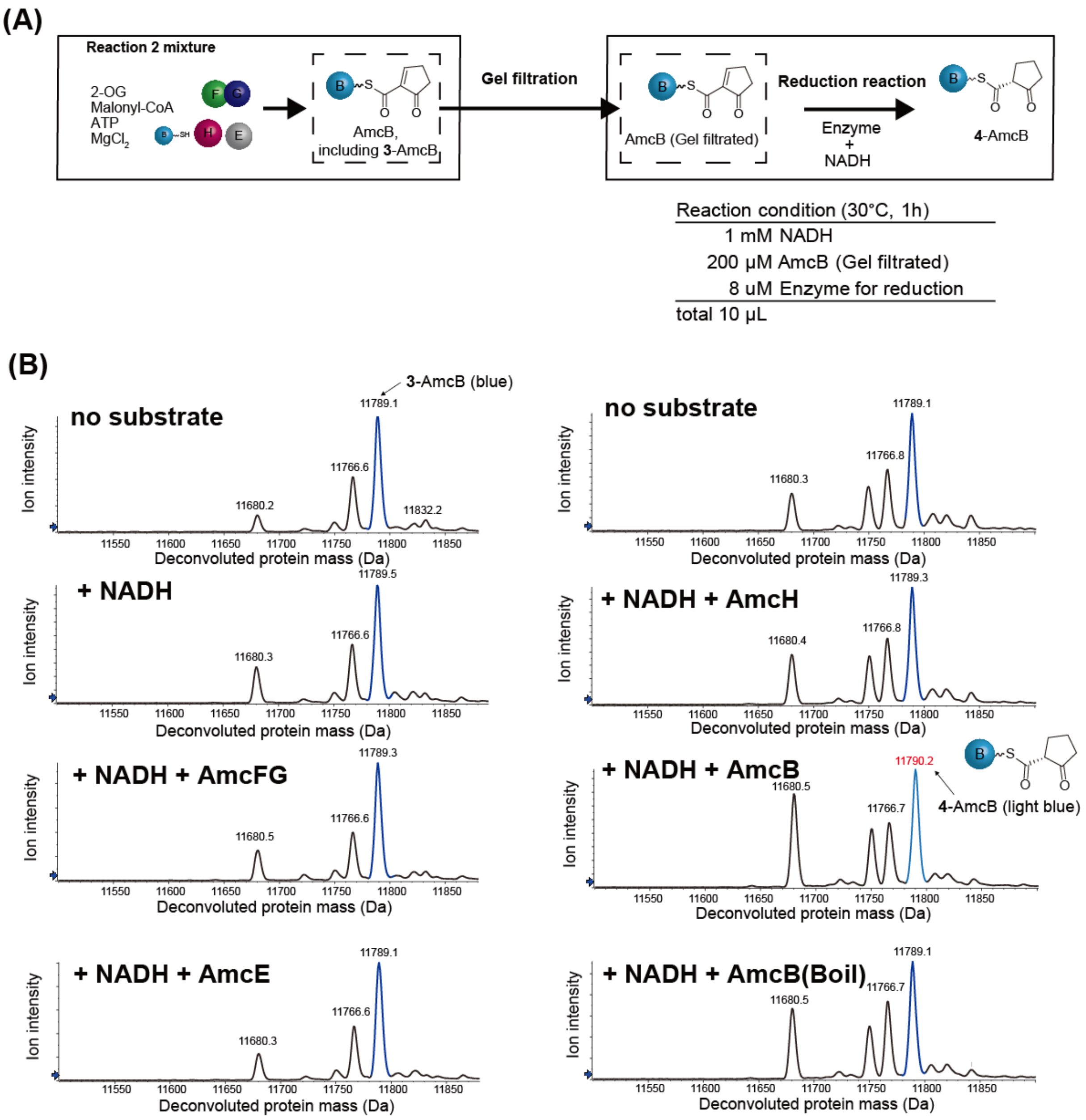
(A)
Reaction 2 mixture
2-OG
Malonyl-CoA
ATP
MgCl2
AmcB,
including 3-AmcB
Gel filtration
AmcB (Gel filtrated)
Reduction reaction
Enzyme
+
NADH
4-AmcB
Reaction condition (30°C, 1h)
1 mM NADH
200 μM AmcB (Gel filtrated)
8 uM Enzyme for reduction
total 10 μL
(B)
no substrate
3-AmcB (blue)
+ NADH
+ NADH + AmcFG
+ NADH + AmcE
+ NADH + AmcH
+ NADH + AmcB
4-AmcB (light blue)
+ NADH + AmcB(Boil)
Ion intensity
Deconvoluted protein mass (Da)

(C)

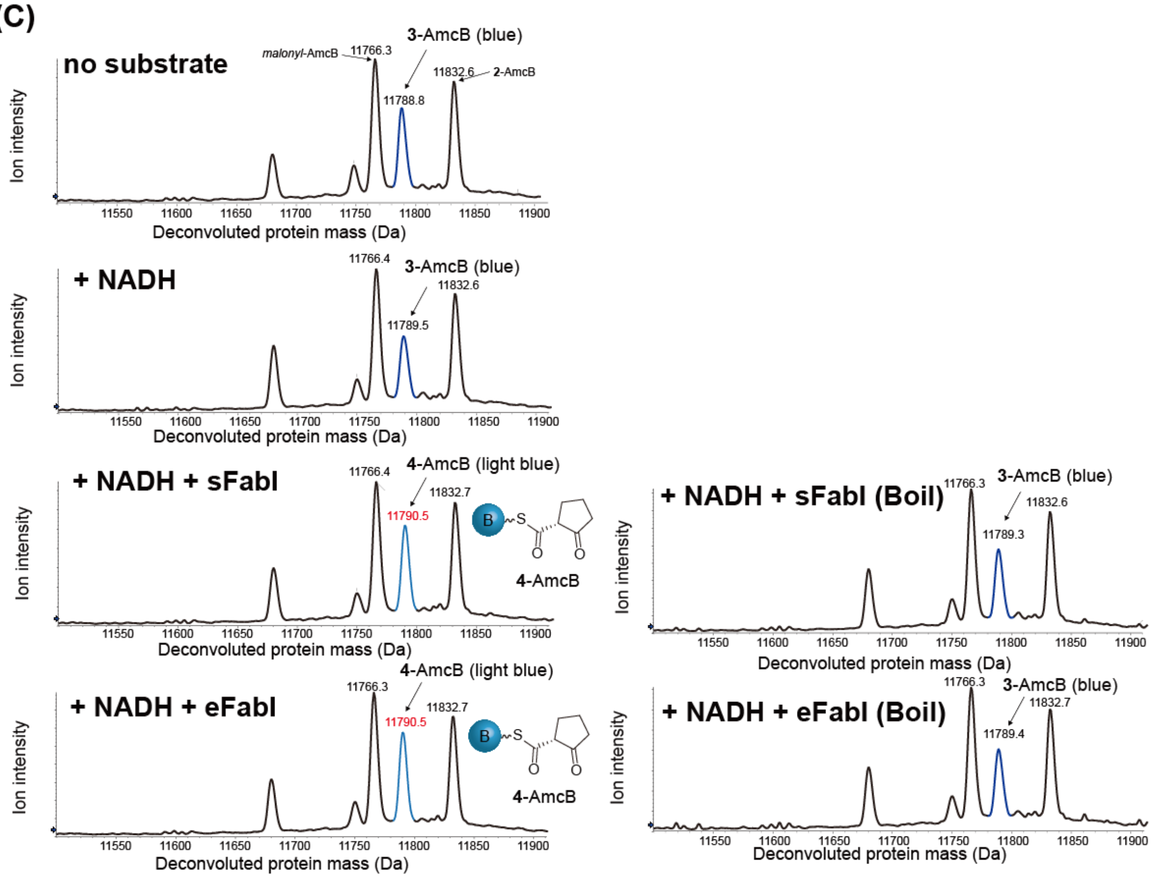

no substrate
malonyl-AmcB
11766.3
3-AmcB (blue)
11832.6
2-AmcB
11788.8
Ion intensity
11550 11600 11650 11700 11750 11800 11850 11900
Deconvoluted protein mass (Da)
+ NADH
11766.4
3-AmcB (blue)
11832.6
11789.5
+ NADH + sFabI
4-AmcB (light blue)
11766.4
11832.7
11790.5
B
S
O
4-AmcB
+ NADH + sFabI (Boil)
11766.3
3-AmcB (blue)
11832.6
11789.3
+ NADH + eFabI
4-AmcB (light blue)
11766.3
11832.7
11790.5
+ NADH + eFabI (Boil)
11766.3
3-AmcB (blue)
11832.7
11789.4

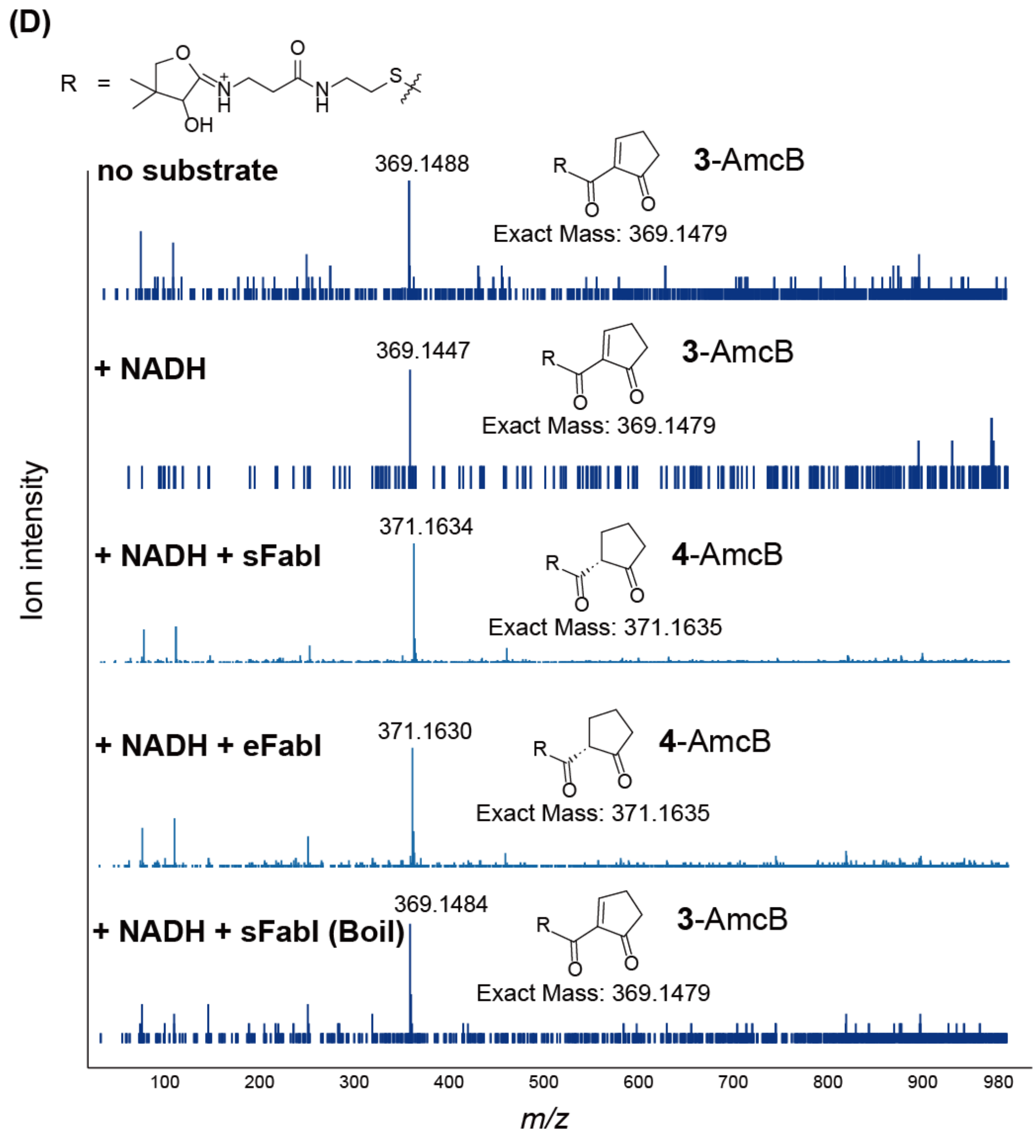

Supplementary Figure 23. Validation of the enzyme that catalyzes the reduction of 3-AmcB. (A) Intermediate-AmcB, including **3**-AmcB used for the reduction, was obtained by the Reaction 2 and then purified by gel filtration. Deconvoluted protein mass spectra were obtained for the reaction product under each condition. MS/MS fragments of the 13+ charge state of intermediate-AmcB were analyzed. The MS/MS fragment of **4**-AmcB was detected only under the condition (+ NADH + AmcB). **4**-AmcB was not detected under boiling conditions, indicating that the reaction is enzyme dependent. (B) Analysis of the reaction solution reacted with sFabI and eFabI. Deconvoluted protein mass spectra for the reaction product under each condition. MS/MS fragments of the 13+ charge state of intermediate-AmcB were analyzed. (C) The MS/MS fragment of **4**-AmcB was detected under the conditions (+ NADH + sFabI, + NADH + eFabI). **4**-AmcB was not detected when boiled FabI was used, indicating that the reaction is FabI dependent.

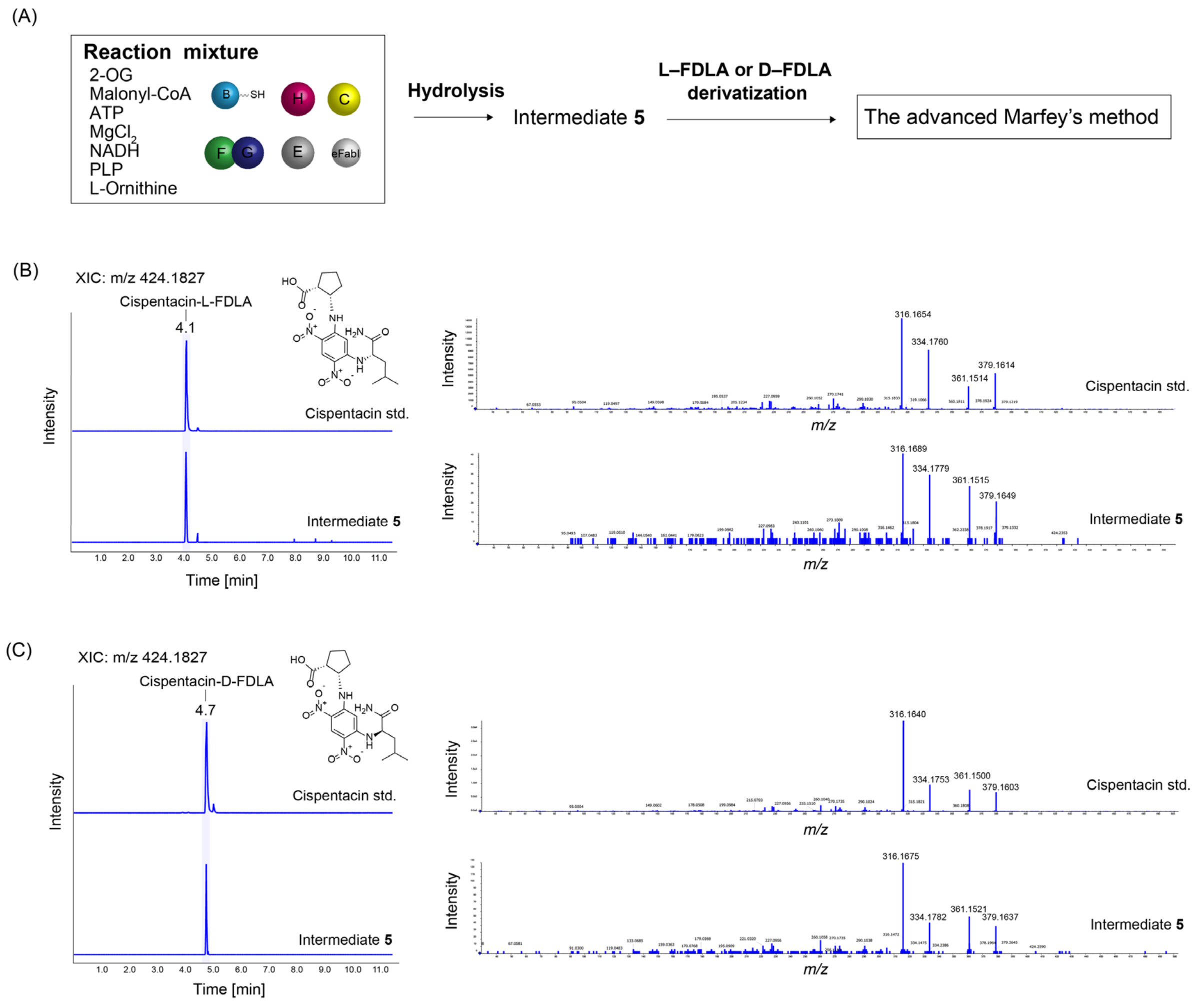

Supplementary Figure 24. LC–MS analysis of FDLA-derivatized cispentacins. (A) **5**-AmcB was prepared in a Reaction 5 mixture and then digested from AmcB via alkaline hydrolysis. The resulting **5**-AmcB hydrolysates were analyzed by the advanced Marfey's method [7, 8, 9]. (B) Extracted ion count (XIC) chromatograms at *m/z* 424.1827 corresponding to the L-FDLA-derivatized intermediate **5** and the L-FDLA-derivatized cispentacin standard. The retention time in LC–MS and the MS/MS spectrum of the derivatized intermediate **5** match well with those of the derivatized standard. (C) XIC chromatograms at *m/z* 424.1827 corresponding to the D-FDLA-derivatized intermediate **5** and the D-FDLA-derivatized cispentacin standard. The retention time in LC–MS and the MS/MS spectrum of the derivatized intermediate **5** match well with those of the derivatized standard.

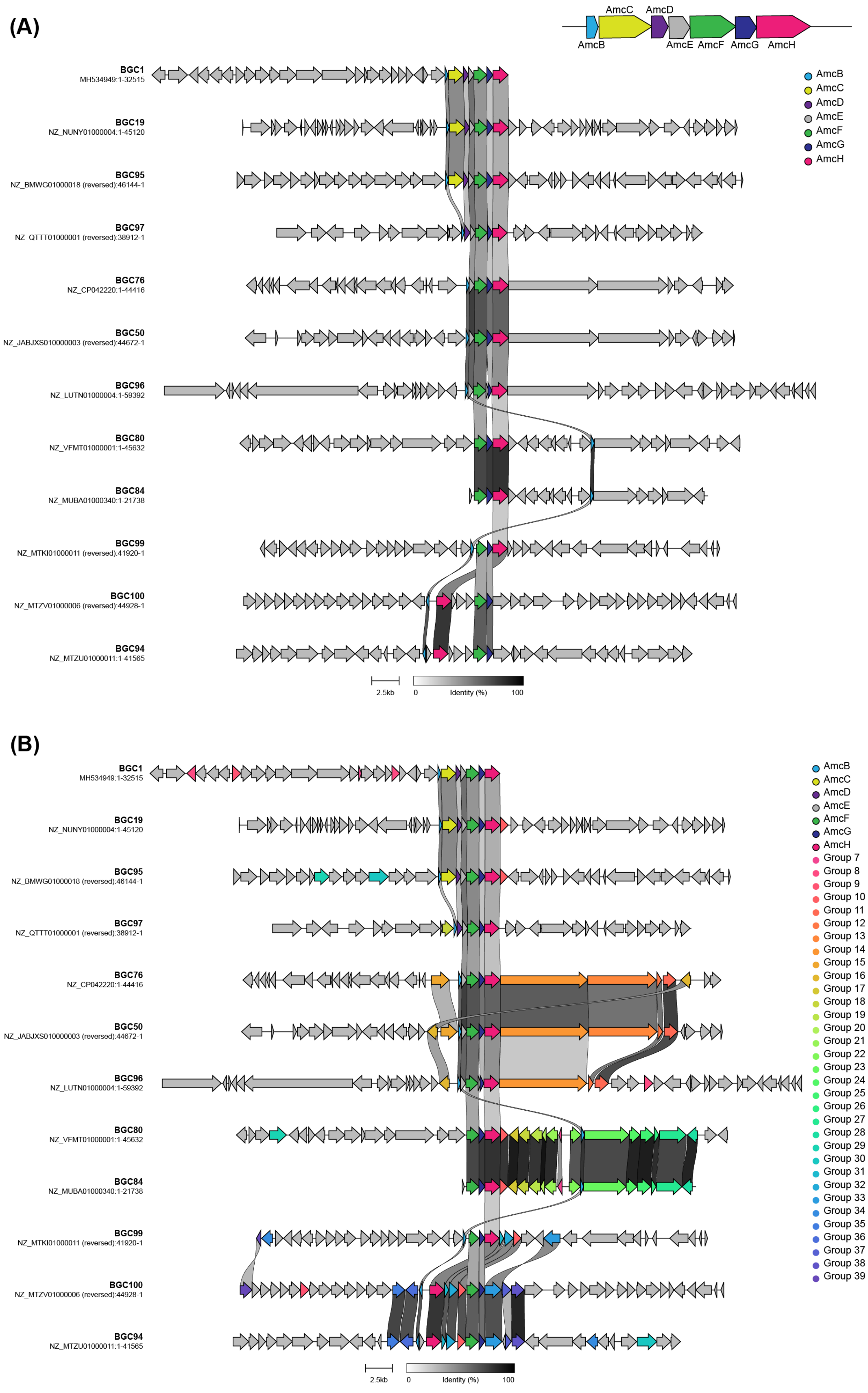
(A)
AmcC
AmcD
AmcB
AmcE
AmcF
AmcG
AmcH
BGC1
MH534949:1-32515
BGC19
NZ_NUNY01000004:1-45120
BGC95
NZ_BMWG01000018 (reversed):46144-1
BGC97
NZ_QTTT01000001 (reversed):38912-1
BGC76
NZ_CP042220:1-44416
BGC50
NZ_JABJXS010000003 (reversed):44672-1
BGC96
NZ_LUTN01000004:1-59392
BGC80
NZ_VFMT01000001:1-45632
BGC84
NZ_MUBA01000340:1-21738
BGC99
NZ_MTKI01000011 (reversed):41920-1
BGC100
NZ_MTZV01000006 (reversed):44928-1
BGC94
NZ_MTZU01000011:1-41565
AmcB
AmcC
AmcD
AmcE
AmcF
AmcG
AmcH
2.5kb
0
Identity (%)
100
(B)
BGC1
MH534949:1-32515
BGC19
NZ_NUNY01000004:1-45120
BGC95
NZ_BMWG01000018 (reversed):46144-1
BGC97
NZ_QTTT01000001 (reversed):38912-1
BGC76
NZ_CP042220:1-44416
BGC50
NZ_JABJXS010000003 (reversed):44672-1
BGC96
NZ_LUTN01000004:1-59392
BGC80
NZ_VFMT01000001:1-45632
BGC84
NZ_MUBA01000340:1-21738
BGC99
NZ_MTKI01000011 (reversed):41920-1
BGC100
NZ_MTZV01000006 (reversed):44928-1
BGC94
NZ_MTZU01000011:1-41565
AmcB
AmcC
AmcD
AmcE
AmcF
AmcG
AmcH
Group 7
Group 8
Group 9
Group 10
Group 11
Group 12
Group 13
Group 14
Group 15
Group 16
Group 17
Group 18
Group 19
Group 20
Group 21
Group 22
Group 23
Group 24
Group 25
Group 26
Group 27
Group 28
Group 29
Group 30
Group 31
Group 32
Group 33
Group 34
Group 35
Group 36
Group 37
Group 38
Group 39
2.5kb
0
Identity (%)
100

Supplementary Figure 25. Organization of *amcG* homolog-containing BGC. The figure was generated using a clinker tool [10]. The numbers for each BGC are based on the Supplementary Data 2. (A) The cispentacin homolog genes are colored. BGC1, 19, and 95 represent the cispentacin type. BGC76, 50, and 96 represent the CFA type. BGC80, 84, 99, 100, and 94 represent the minimal type. BGC97 lacking AmcC does not belong to any type. (B) In addition to the cispentacin homologous genes, the other homologous genes (identity threshold: 0.3) are also colored.

(A)

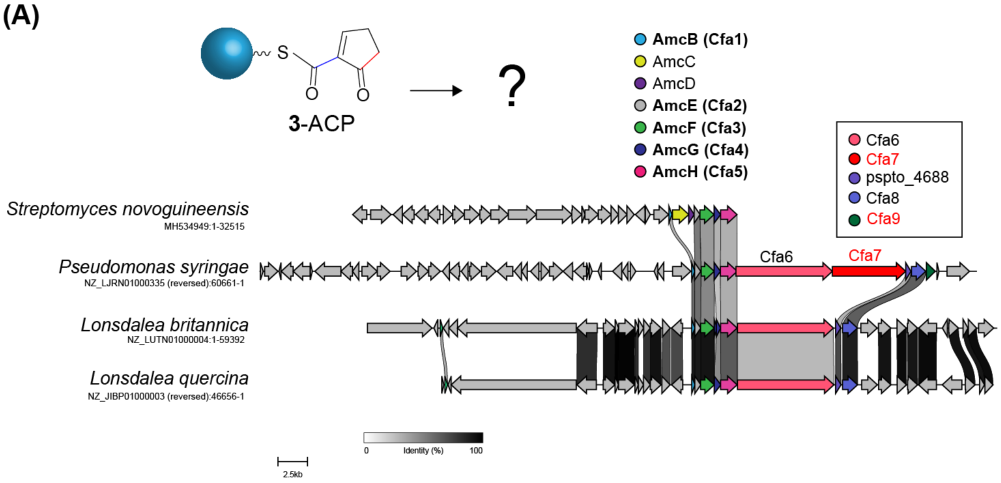

(B)

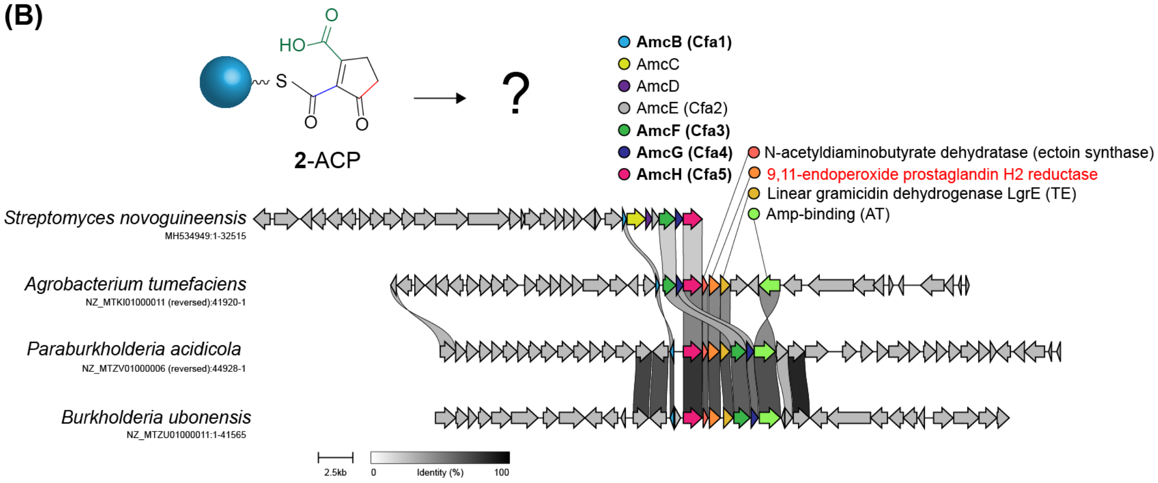

Supplementary Figure 26. Representatives of the *amcG* homolog-containing BGCs for unknown natural products. (A) CFA-type BGCs of *Lonsdalea britannica* and *Lonsdalea quercina cfa7* (type I PKS) and *cfa9*, both of which are CFA biosynthesis genes of *Pseudomonas syringae*, are absent in these BGCs. (B) Minimal-type BGCs of *Agrobacterium tumefaciens*, *Paraburkholderia acidicola*, and *Burkholeria ubonensis*. 9,11-Endoperoxide prostaglandin H2 reductase and several genes are conserved in these BGCs.

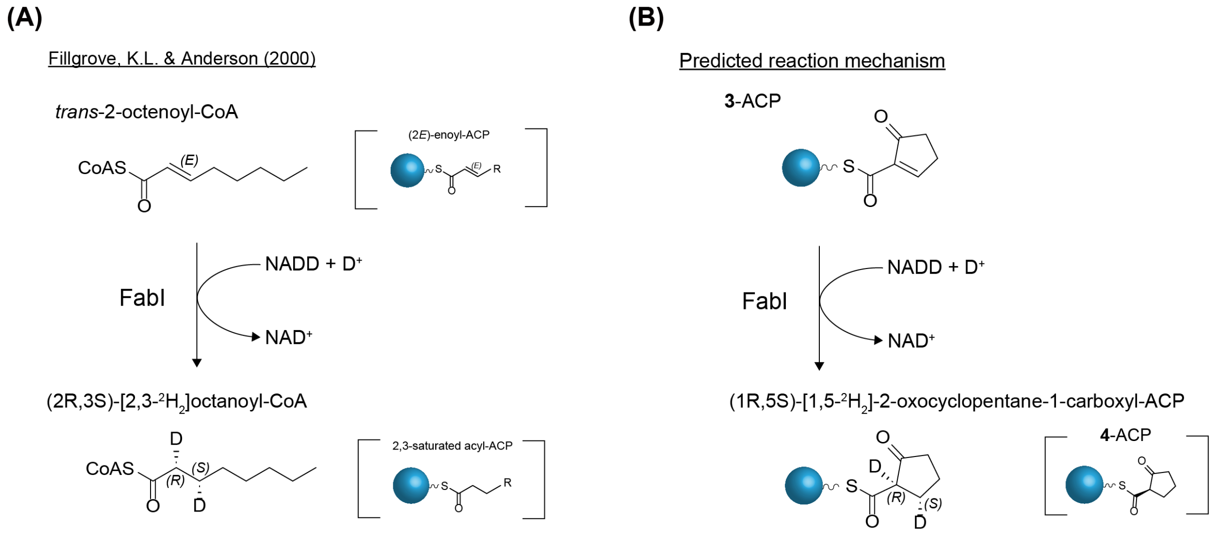

Supplementary Figure 27. FabI-catalyzed reduction. (A) FabI catalyzes the *syn* addition of 2H to the C2–C3 double bond of fatty acid intermediates. Labeling experiments with 4-^{2}H-labeled β-nicotinamide adenine dinucleotide phosphate (NADD) in D_2O revealed that FabI catalyzes the stereoselective reduction of the C2–C3 double bond of fatty acid intermediates to yield (*R*)-C2 and (*S*)-C3 [11]. (B) By analogy with fatty acid biosynthesis, FabI should catalyze the *syn* addition of 2H to the double bond of **3**-ACP to yield (1*R*)-2-oxocyclopentane-1-carboxyl-ACP (**4**-ACP). The stereochemistry of C1 in **4**-ACP is identical to that of C1 in cispentacin.

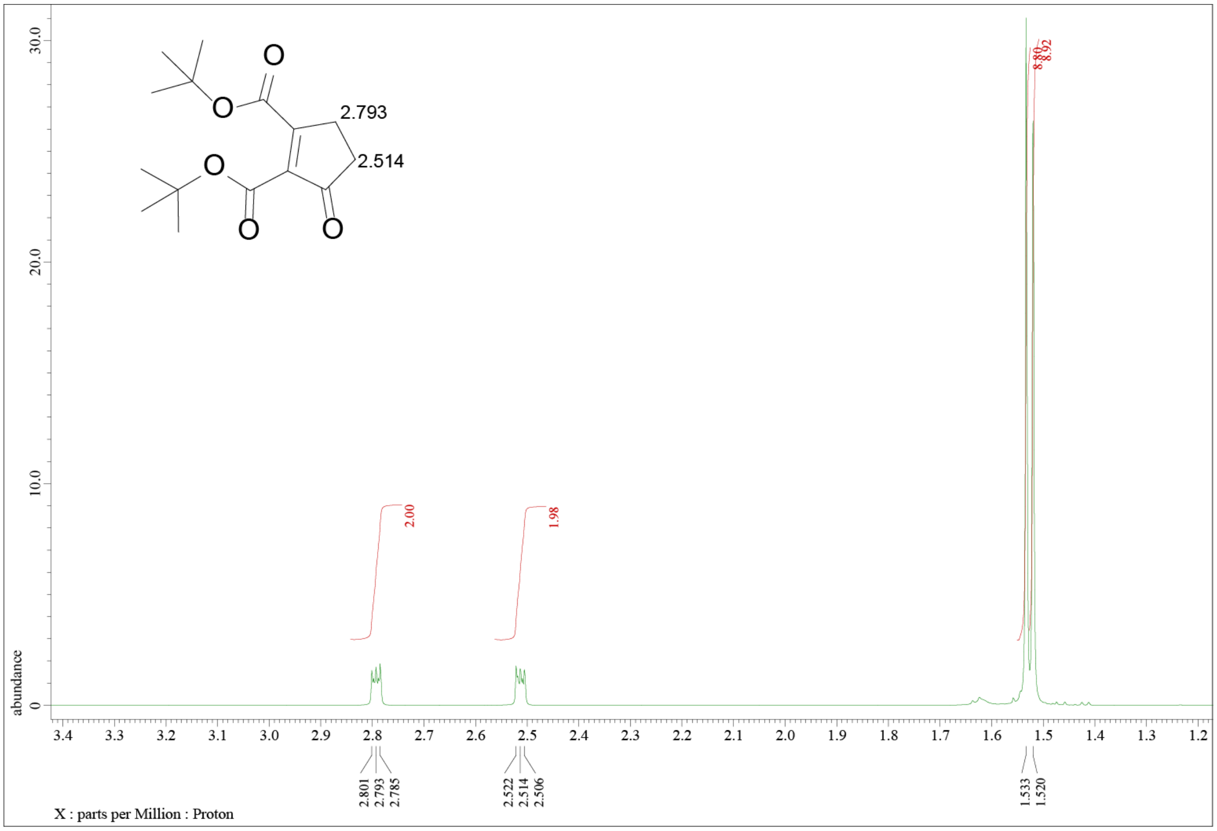

Supplementary Figure 28A. ^{1}H NMR spectrum of 2,3-di(carbo-*tert*-butoxy)-2-cyclopentenone in $CDCl_3$ at 600 Hz.

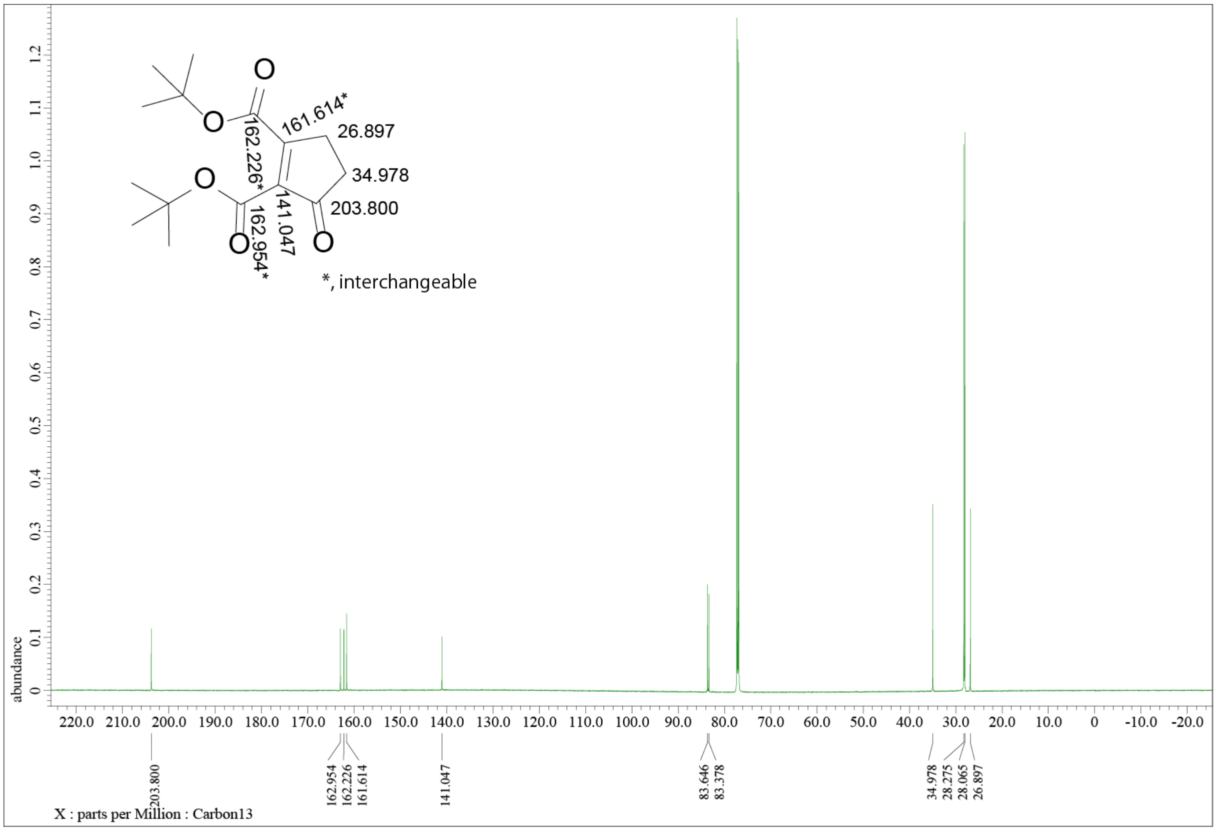

Supplementary Figure 28B. ^{13}C NMR spectrum of 2,3-di(carbo-*tert*-butoxy)-2-cyclopentenone in $CDCl_3$ at 150 Hz.

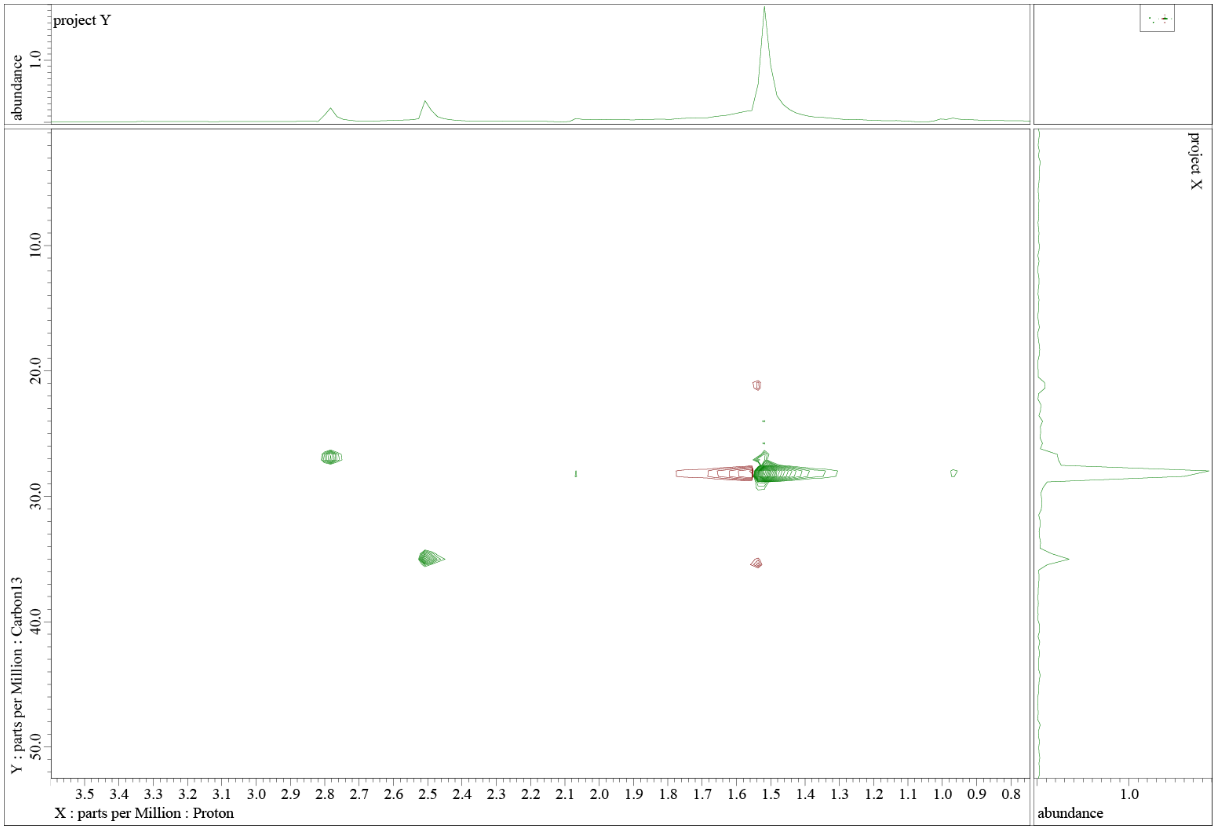

Supplementary Figure 28C. HSQC spectrum of 2,3-di(carbo-*tert*-butoxy)-2-cyclopentenone in $CDCl_3$.

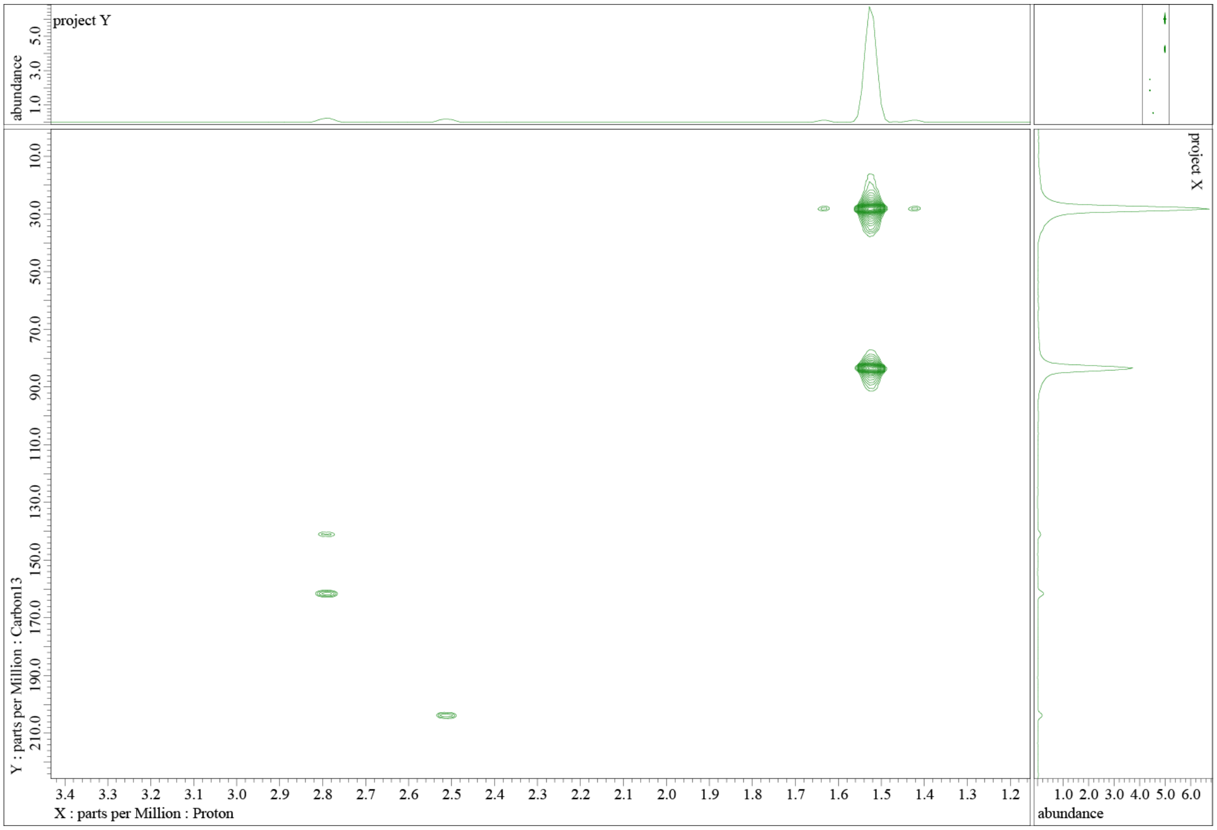

Supplementary Figure 28D. HMBC spectrum of 2,3-di(carbo-*tert*-butoxy)-2-cyclopentenone in $CDCl_3$.

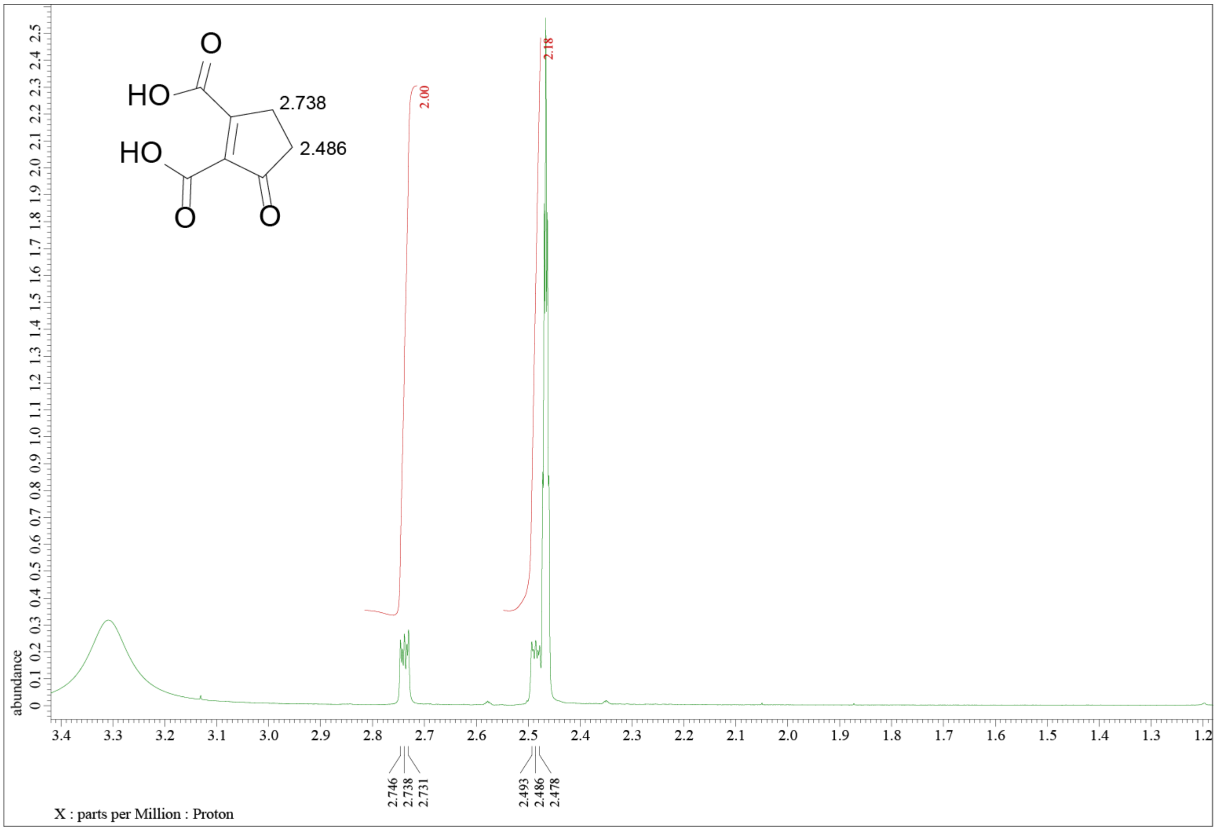

Supplementary Figure 29A. ^{1}H NMR spectrum of 3-oxocyclopent-1-ene-1,2-dicarboxylic acid in DMSO-d_6 at 600 Hz.

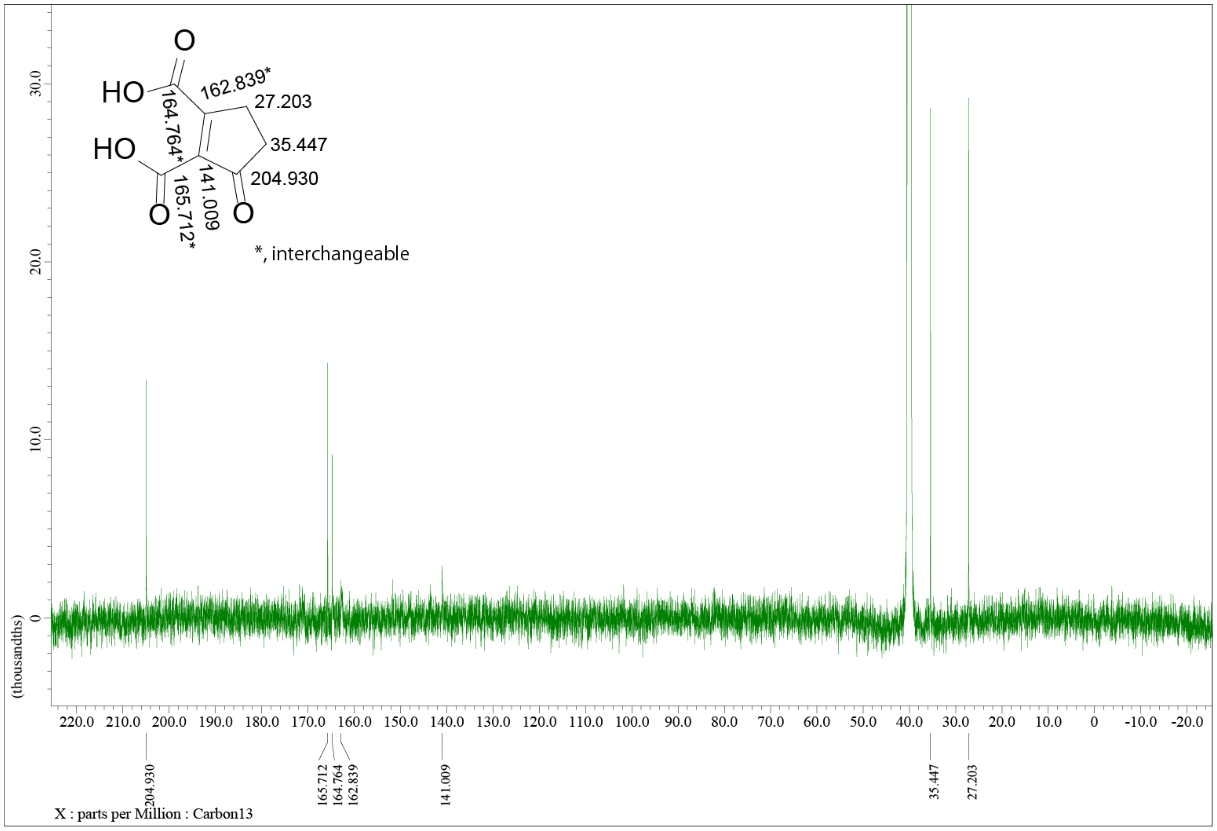

Supplementary Figure 29B. ^{13}**C NMR spectrum of 3-oxocyclopent-1-ene-1,2-dicarboxylic acid in DMSO-*d*$_6$ at 150 Hz.**

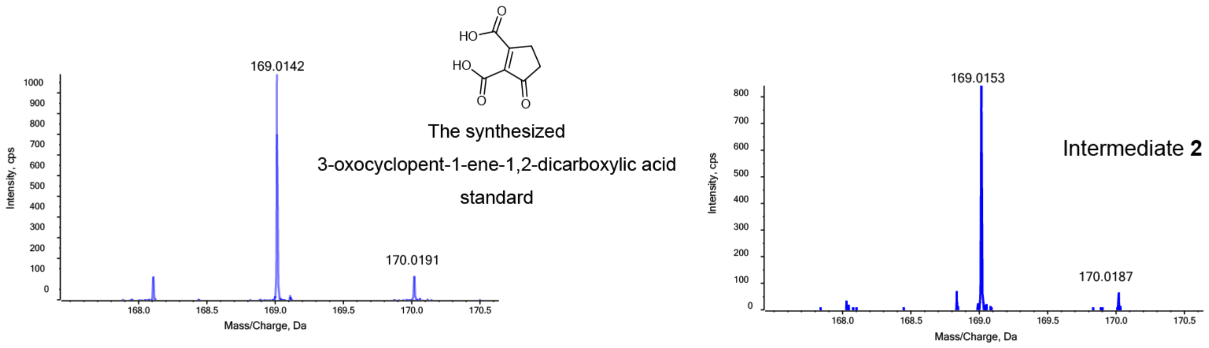

Supplementary Figure 30. HRMS spectra of 3-oxocyclopent-1-ene-1,2-dicarboxylic acid.

Supplementary References

(1) Dairi, T., Aisaka, K., Katsumata, R. & Hasegawa, M. A self-defense gene homologous to tetracycline effluxing gene essential for antibiotic production in Streptomyces aureofaciens. *Biosci. Biotechnol. Biochem.* **59**, 1835-1841 (1995).

(2) Romo, A.J. et al. The amipurimycin and miharamycin biosynthetic gene clusters: unraveling the origins of 2-aminopurinyl peptidyl nucleoside antibiotics. *J. Am. Chem. Soc.* **141**, 14152-14159 (2019).

(3) Jez, J.M., Ferrer, J.-L., Bowman, M.E., Dixon, R.A. & Noel, J.P. Dissection of malonyl-coenzyme A decarboxylation from polyketide formation in the reaction mechanism of a plant polyketide synthase. *Biochemistry* **39**, 890-902 (2000).

(4) Meguro, A. et al. An unusual terpene cyclization mechanism involving a carbon‑carbon bond rearrangement. *Angew. Chem. Int. Ed.* **54**, 4353-4356 (2015).

(5) Du, D., Katsuyama, Y., Shin ‑ ya, K. & Ohnishi, Y. Reconstitution of a type II polyketide synthase that catalyzes polyene formation. *Angew. Chem.* **130**, 1972-1975 (2018).

(6) Saitou, N. & Nei, M. The neighbor-joining method: a new method for reconstructing phylogenetic trees. *Mol. Biol. Evol.* **4**, 406-425 (1987).

(7) Marfey, P. Determination of D-amino acids. II. Use of a bifunctional reagent, 1, 5-difluoro-2, 4-dinitrobenzene. *Carlsberg Res. Commun.* **49**, 591-596 (1984).

(8) Fujii, K., Ikai, Y., Oka, H., Suzuki, M. & Harada, K.-i. A nonempirical method using LC/MS for determination of the absolute configuration of constituent amino acids in a peptide: combination of Marfey's method with mass spectrometry and its practical application. *Anal. Chem.* **69**, 5146-5151 (1997).

(9) Fujii, K. et al. A nonempirical method using LC/MS for determination of the absolute configuration of constituent amino acids in a peptide: Elucidation of limitations of Marfey's method and of its separation mechanism. *Anal. Chem.* **69**, 3346-3352 (1997).

(10) Gilchrist, C.L. & Chooi, Y.-H. Clinker & clustermap. js: Automatic generation of gene cluster comparison figures. *Bioinformatics* **37**, 2473-2475 (2021).

(11) Fillgrove, K.L. & Anderson, V.E. Orientation of Coenzyme A Substrates, Nicotinamide and Active Site Functional Groups in (Di) enoyl− coenzyme A Reductases. *Biochemistry* **39**, 7001-7011 (2000).
