# Supplementary material for: Discovery of type II polyketide synthase-like enzymes for the biosynthesis of cispentacin
Source: Nat Commun. 2023 Dec 6;14:8065. doi: 10.1038/s41467-023-43731-z (PMC10698177; doi:10.1038/s41467-023-43731-z)
Supplement: Supplementary file 3 — Description of Additional Supplementary Files [file 41467_2023_43731_MOESM3_ESM.pdf]

Description of Additional Supplementary Files

Supplementary Data 1: Results of the hmmer search

Supplementary Data 2: Output file of a BLASTP search
