# Supplementary material for: Discovery of type II polyketide synthase-like enzymes for the biosynthesis of cispentacin
Source: Nat Commun. 2023 Dec 6;14:8065. doi: 10.1038/s41467-023-43731-z (PMC10698177; doi:10.1038/s41467-023-43731-z)
Supplement: Supplementary file 4 — Supplementary Data 1 [file 41467_2023_43731_MOESM4_ESM.pdf]

Results of the hmmer search

Phylum	Class	
Proteobacteria	Gammaproteobacteria	63
	Deltaproteobacteria	20
	Betaproteobacteria	7
	Alphaproteobacteria	4
Actinobacteria	Actinomycetia	66
Firmicutes	Bacilli	8
Ignavibacteriae	Ignavibacteriae bacterium	1
	total	169

	Species with AmcG homologs	E-value
1	Streptomyces novoguineensis	1.1E-122
2	Streptomyces sp.	1.1E-122
3	Streptomyces sp. GMR22	2.3E-102
4	Streptomyces violaceusniger	4.1E-101
5	Streptomyces sp. AgN23	4.1E-101
6	Streptomyces antimycoticus	1.3E-99
7	Streptomyces antimycoticus	6.9E-99
8	Streptomyces haliclonae	2.7E-96
9	Desertiactinospora gelatinilytica	1.2E-61
10	Sphaerisporangium sp. LHW63015	5E-61
11	Kibdelosporangium sp. MJ126-NF4	1.1E-47
12	Streptomyces sp. GMR22	4.2E-45
13	Streptomyces sp. NRRL WC-3618	1.1E-42
14	Kribbella italica	1.4E-42
15	Streptomyces sp. LBUM 1475	1.5E-41
16	Streptomyces scabiei (strain 87.22)	1.5E-41
17	Streptomyces sp. LBUM 1480	1.5E-41
18	Streptomyces sp. LBUM 1482	1.5E-41
19	Streptomyces griseoruber	4.1E-41
20	Streptomyces scabiei	3.9E-41

21	Streptomyces sp. NRRL F-4489	2.4E-40
22	Bacillus anthracis	2.9E-40
23	Streptomyces misionensis	8.8E-40
24	Actinosynnema sp. ALI-1.44	8.7E-40
25	Streptomyces sp. F-1	1.2E-39
26	Nonomuraea sp. KC401	1.6E-39
27	Streptomyces cadmiisoli	2.2E-39
28	Streptomyces sp. JV178	2.8E-39
29	Bacillus fungorum	3E-39
30	Nonomuraea pusilla	3.6E-39
31	Bacillus anthracis	4.9E-39
32	Bacillus cereus	6.3E-39
33	Bacillus cereus	6.6E-39
34	Bacillus thuringiensis serovar pingluonsis	6.6E-39
35	Bacillus thuringiensis	6.6E-39
36	Streptomyces sp. NEAU-sy36	1.1E-38
37	Bacillus cereus Rock3-42	3.4E-38
38	Actinoalloteichus hymeniacidonis	6.5E-38
39	Xanthomonas campestris	8.6E-38
40	Streptomyces morookaense	2.6E-37
41	Pseudomonas coronafaciens pv. coronafaciens	3E-37
42	Xanthomonas arboricola	3.9E-37
43	Pseudomonas syringae pv. actinidiae	5.4E-37
44	Pseudomonas cannabina	5.4E-37
45	Pseudomonas amygdali pv. morsprunorum	5.4E-37
46	Pseudomonas amygdali pv. ulmi	5.4E-37
47	Pseudomonas syringae pv. tomato	5.4E-37
48	Pseudomonas syringae	5.4E-37
49	Pseudomonas syringae pv. spinaceae	5.4E-37
50	Pseudomonas savastanoi pv. glycinea	5.4E-37
51	Pseudomonas syringae pv. maculicola	5.4E-37
52	Streptomyces griseocarneus	6.2E-37
53	Pseudonocardia sp. HH130630-07	9.2E-37
54	Pseudomonas coronafaciens pv. zizaniae	1.2E-36

55	Pseudomonas cannabina	1.2E-36
56	Pseudomonas cannabina pv. alisalensis (strain ATCC BAA-566 / CFBP 6866 / ICMP 15200 / BS91)	1.2E-36
57	Pseudomonas syringae pv. tomato	3.3E-36
58	Pseudomonas coronafaciens pv. zizaniae	3.3E-36
59	Pseudomonas syringae pv. tomato (strain ATCC BAA-871 / DC3000)	3.3E-36
60	Lonsdalea iberica	3.4E-36
61	Pseudomonas coronafaciens pv. oryzae	3.7E-36
62	Pseudomonas cannabina pv. alisalensis	3.7E-36
63	Pseudomonas amygdali pv. aesculi	1.1E-35
64	Pseudomonas coronafaciens pv. atropurpurea	7.7E-36
65	Actinobacteria bacterium	1.5E-35
66	Lonsdalea britannica	1.8E-35
67	Kribbella sandramycini	3E-35
68	Streptomyces kasugaensis	1.6E-34
69	Streptomyces sp. 1331.2	4.4E-34
70	Streptomyces sp. SID7805	4E-34
71	Streptomyces sp. CB02959	4.1E-34
72	Brenneria sp. CFCC 11842	4.9E-34
73	Streptomyces sp. 1114.5	6.1E-34
74	Micromonospora sp. KC721	1E-33
75	Streptomyces albulus	1.6E-33
76	Streptomyces sp. SID8354	2.4E-33
77	Zymobacter palmae	3.4E-33
78	Streptomyces albulus	3.7E-33
79	Nonomuraea solani	8.3E-33
80	Streptomyces sp. 769	1.1E-32
81	Lentzea xinjiangensis	1.8E-32
82	Streptomyces sp. NRRL F-4489	4.7E-32
83	Streptomyces sp. SID2955	5.6E-32
84	Streptomyces sp. SID161	6.9E-32
85	Streptomyces sp. SID486	6.9E-32
86	Azospirillum sp. B510	7.6E-32
87	Pectobacterium carotovorum	9.4E-32
88	Pectobacterium brasiliense	9.4E-32

89	Pectobacterium peruviense	9.4E-32
90	Actinophytocola oryzae	9.8E-32
91	Pectobacterium betavasculorum	1.4E-31
92	Actinomadura sp. CNU-125	1.6E-31
93	Pectobacterium actinidiae	2.9E-30
94	Brenneria alni	3.9E-30
95	Streptomyces noursei	3.6E-30
96	Pectobacterium atrosepticum (strain SCRI 1043 / ATCC BAA-672)	5.9E-30
97	Pectobacterium atrosepticum	5.9E-30
98	Brenneria salicis ATCC 15712 = DSM 30166	3E-29
99	Streptomyces sp. SLBN-115	6.1E-29
100	Actinobacteria bacterium OV320	6.3E-29
101	Streptomyces sp. Root1310	8.7E-29
102	Streptomyces sp. CB03238	1.8E-28
103	Streptomyces sp. B9173	2.2E-28
104	Brenneria sp. EniD312	2.2E-28
105	Brenneria nigrifluens DSM 30175 = ATCC 13028	2.2E-28
106	Dickeya poaceiphila	5.3E-28
107	Streptomyces cacaoi subsp. asoensis	5.4E-28
108	Allokutzneria albata	1.1E-27
109	Streptomyces galilaeus	5E-27
110	Streptomyces sp. Z022	9.6E-27
111	Paraburkholderia acidophila	4.3E-23
112	Nguyenibacter vanlangensis	9.1E-22
113	Pseudomonas acidophila	1.9E-21
114	Rhizobium radiobacter	2.2E-21
115	Thermomonospora umbrina	2.5E-21
116	Pseudomonas syringae pv. maculicola	3.2E-19
117	Minicystis rosea	6.5E-19
118		2E-18
119	Stigmatella aurantiaca (strain DW4/3-1)	1.2E-17
120	Vitiosangium sp. GDMCC 1.1324	1.6E-17
121	Myxococcus fulvus	2.4E-17
122	Myxococcus stipitatus (strain DSM 14675 / JCM 12634 / Mx s8)	1.3E-16

123	Kitasatospora niigatensis	2E-16
124	Myxococcus hansupus	4.6E-16
125	Archangium gephyra	6.1E-16
126	Pseudomonas sp. GV085	1.2E-15
127	Pseudomonas sp. OV397	1.2E-15
128	Pseudomonas sp. CMR5c	1.7E-15
129	Pseudomonas aestus	1.7E-15
130	Archangium sp. Cb G35	1.6E-15
131	Archangium violaceum Cb vi76	1.6E-15
132	Pseudomonas piscis	2.4E-15
133	Pseudomonas sp. FW507-12TSA	2.4E-15
134	Pseudomonas batumici	2.1E-15
135	Myxococcus sp. AM010	4.9E-15
136	Pyxidicoccus fallax	1.6E-14
137	Corallococcus sp. CA047B	2.7E-14
138	Corallococcus llansteffanensis	3.3E-14
139	Corallococcus sp. CA053C	3.6E-14
140	Corallococcus terminator	8E-14
141	Corallococcus sicarius	2.7E-13
142	Myxococcaceae bacterium	9.9E-13
143	Deltaproteobacteria bacterium RIFCSPLOWO2_02_FULL_53_8	8.6E-13
144	Pseudomonas sp. 22 E 5	1.8E-12
145	Pseudomonas extremorientalis	1.8E-12
146	Corallococcus sp. H22C18031201	1.8E-12
147	Burkholderia oklahomensis C6786	2.2E-12
148	Burkholderia sp. BDU8	4.5E-12
149	Burkholderia sp. ABCPW 14	5.7E-12
150	Pseudomonas canadensis	9E-12
151	Paraburkholderia phenazinium	1.1E-11
152	Pseudomonas sp. C 49-2	1E-11
153	Thiomonas sp. X19	2E-11
154	Paraburkholderia phenazinium	2.6E-11
155	Ignavibacteriae bacterium	2.4E-11
156	Pseudomonas sp. FSL R10-2245	5E-11

157	Pseudomonas sp. SJZ103	5.9E-11
158	Enterobacter kobei	2.5E-10
159	Enterobacter sp. AM17-18	3.3E-10
160	Enterobacter kobei	4.2E-10
161	Lelliottia nimipressuralis	4.7E-10
162	Enterobacter sp. WCHEn090032	5.6E-10
163	Enterobacter cloacae complex sp	6.4E-10
164	Enterobacter chengduensis	7.2E-10
165	Enterobacter cloacae	8.5E-10
166	Enterobacter kobei	8.5E-10
167	Enterobacter agglomerans	1.4E-09
168	Streptomyces phaeoluteigriseus	0.000000002
169	Mesorhizobium sp.	5.3E-09
