# Supplementary material for: Discovery of type II polyketide synthase-like enzymes for the biosynthesis of cispentacin
Source: Nat Commun. 2023 Dec 6;14:8065. doi: 10.1038/s41467-023-43731-z (PMC10698177; doi:10.1038/s41467-023-43731-z)
Supplement: Supplementary file 5 — Supplementary Data 2 [file 41467_2023_43731_MOESM5_ESM.pdf]

List of each cluster (numbering, Accession of organisms represented)
100 amcG-containing BGCs, ordered from BGCs containing AmcG with the highest compatibility (score).

blastp
Iteration: 0
Query: amcG
RID: 9K81Z774016
Database: nr_cluster_seq
Fields:

the number of BGC	# 100 hits found	subject acc.ver	% identity	alignment length	mismatches	gap opens	q. start	q. end	s. start	s. end	evalue	bit score	% positives
1	amcG	QCX41961.1	100	184	0	0	1	184	1	184	3.38E-128	367	100
2	amcG	WP_189561794.1	89.011	182	20	0	1	182	1	182	4.37E-112	327	93.41
3	amcG	WP_182468098.1	84.615	182	28	0	1	182	1	182	5.26E-105	308	90.66
4	amcG	MBB4673076.1	82.222	180	32	0	1	180	1	180	2.59E-98	292	87.78
5	amcG	WP_162795002.1	61.006	159	62	0	20	178	6	164	1.74E-59	192	74.21
6	amcG	WP_111167667.1	61.392	158	61	0	20	177	16	173	1.02E-52	176	74.05
7	amcG	WP_113985346.1	60.366	164	65	0	20	183	12	175	1.33E-52	175	71.95
8	amcG	WP_209647138.1	51.572	159	75	2	19	177	13	169	1.11E-46	160	69.81
9	amcG	WP_052479233.1	50.92	163	77	2	15	177	6	165	4.99E-44	153	65.03
10	amcG	WP_105967719.1	48.276	174	85	3	15	184	7	179	5.59E-40	143	60.34
11	amcG	WP_184795263.1	49.057	159	77	2	19	177	8	162	3.38E-39	140	64.78
12	amcG	MBV9022849.1	48.765	162	81	2	17	177	9	169	5.18E-39	140	62.96
13	amcG	WP_182471800.1	51.825	137	66	0	41	177	37	173	6.67E-39	140	66.42
14	amcG	WP_024802760.1	47.826	161	83	1	20	179	16	176	1.21E-38	140	60.87
15	amcG	WP_055634613.1	47.619	168	82	3	15	177	7	173	1.42E-37	137	60.12
16	amcG	WP_169393912.1	48.611	144	74	0	37	180	31	174	2.96E-37	136	64.58
17	amcG	WP_086756671.1	46.842	190	83	6	1	182	1	180	4.65E-37	135	57.37
18	amcG	WP_059080167.1	46.914	162	84	2	17	177	9	169	3.83E-36	133	63.58
19	amcG	WP_098276132.1	41.667	144	84	0	27	170	26	169	4.52E-36	133	59.03
20	amcG	WP_242226323.1	44.882	127	70	0	33	159	1	127	4.08E-35	130	62.2
21	amcG	WP_076986735.1	47.771	157	75	2	19	175	4	153	2.67E-34	128	62.42
22	amcG	MCE7004706.1	47.887	142	73	1	39	180	40	180	8.12E-34	127	65.49
23	amcG	WP_138200933.1	47.518	141	73	1	40	180	41	180	2.02E-33	127	65.25
24	amcG	WP_053761438.1	44.311	167	91	2	15	180	16	181	2.12E-33	127	57.49
25	amcG	WP_052422984.1	46.575	146	77	1	39	184	40	184	2.50E-33	126	65.75
26	amcG	WP_058767951.1	51.408	142	68	1	42	183	34	174	5.85E-33	125	61.97
27	amcG	WP_069846947.1	45.89	146	78	1	39	184	40	184	1.38E-32	124	64.38
28	amcG	WP_184622999.1	50.704	142	69	1	42	183	34	174	3.72E-32	123	61.97
29	amcG	WP_164386746.1	48.322	149	62	2	39	180	40	180	5.23E-32	123	59.73
30	amcG	WP_165956521.1	48.201	139	64	2	39	177	19	149	1.64E-31	120	64.75
31	amcG	RLV10181.1	47.917	144	74	1	41	184	29	171	4.09E-31	120	58.33
32	amcG	WP_171079069.1	47.159	176	84	5	1	176	1	167	7.80E-31	119	55.68
33	amcG	KPB94666.1	51.471	136	65	1	42	177	15	149	1.18E-30	119	62.5
34	amcG	WP_070024088.1	47.879	165	79	4	19	177	9	172	1.33E-30	120	61.82
35	amcG	WP_171675246.1	48.485	132	61	2	33	164	1	125	1.85E-30	117	63.64
36	amcG	WP_054990124.1	51.471	136	65	1	42	177	34	168	2.56E-30	118	62.5
37	amcG	WP_177227589.1	49.375	160	77	3	21	177	393	551	4.24E-30	125	62.5
38	amcG	WP_094109901.1	41.875	160	91	2	19	177	11	169	5.73E-30	117	59.38
39	amcG	WP_191140370.1	34.375	160	105	0	19	178	18	177	8.67E-30	117	58.13

40	amcG	WP_051616280.1	42.308	156	88	2	23	177	15	169	1.25E-29	117	58.97
41	amcG	WP_085653152.1	42.593	162	91	2	17	177	9	169	1.94E-29	116	57.41
42	amcG	WP_066982330.1	40.805	174	102	1	7	180	2	174	2.85E-29	116	58.62
43	amcG	TMM37893.1	47.794	136	70	1	41	176	31	165	4.45E-29	115	60.29
44	amcG	KPW22679.1	52.206	136	64	1	42	177	458	592	1.71E-28	121	63.24
45	amcG	WP_136167060.1	40.244	164	96	2	16	178	8	170	2.70E-28	113	58.54
46	amcG	WP_228321346.1	46.296	162	85	2	20	180	11	171	4.47E-28	112	55.56
47	amcG	WP_132261363.1	44.898	147	80	1	37	183	25	170	4.76E-28	112	53.06
48	amcG	WP_131124666.1	45.139	144	79	0	41	184	29	172	5.22E-28	112	55.56
49	amcG	WP_068798146.1	50.725	138	67	1	41	178	22	158	1.52E-27	111	61.59
50	amcG	WP_172289556.1	45.455	121	66	0	42	162	35	155	3.68E-27	110	62.81
51	amcG	WP_053666612.1	44.937	158	86	1	20	177	15	171	5.49E-27	110	61.39
52	amcG	WP_100602528.1	40.936	171	98	1	14	184	5	172	1.03E-26	109	50.88
53	amcG	WP_038524297.1	46.207	145	76	2	41	184	29	172	1.21E-26	108	57.24
54	amcG	WP_018537882.1	40.462	173	100	1	12	184	3	172	1.21E-26	108	50.87
55	amcG	WP_211763030.1	47.445	137	66	2	41	177	18	148	1.98E-26	108	54.74
56	amcG	WP_027704559.1	40.964	166	94	3	13	177	7	169	2.09E-26	108	58.43
57	amcG	WP_039313139.1	37.195	164	101	2	22	184	14	176	2.34E-26	108	56.71
58	amcG	WP_052293700.1	40	165	93	3	20	182	12	172	2.79E-26	108	53.33
59	amcG	WP_214636464.1	41.722	151	87	1	32	182	20	169	6.16E-26	108	54.97
60	amcG	WP_161293594.1	45	140	76	1	41	180	25	163	9.33E-26	107	59.29
61	amcG	WP_121174718.1	48.905	137	67	2	41	176	30	164	1.02E-25	107	60.58
62	amcG	WP_103963945.1	38.125	160	98	1	18	177	3	161	1.04E-25	106	55
63	amcG	WP_225447214.1	41.975	162	93	1	22	182	404	565	1.51E-25	112	56.17
64	amcG	BAI73721.1	42.361	144	79	2	39	182	6	145	4.63E-25	104	54.86
65	amcG	WP_066984015.1	45.517	145	78	1	41	184	29	173	5.04E-25	105	53.79
66	amcG	WP_063351990.1	44.526	137	76	0	41	177	29	165	7.96E-25	104	55.47
67	amcG	WP_240630143.1	35.976	164	103	2	22	184	15	177	1.87E-24	103	55.49
68	amcG	WP_189205086.1	45.342	161	85	3	22	182	9	166	2.07E-24	103	58.39
69	amcG	WP_089956931.1	44.203	138	77	0	41	178	23	160	2.68E-24	103	55.8
70	amcG	WP_208298038.1	45.695	151	76	4	31	176	16	165	3.42E-24	103	58.28
71	amcG	WP_233580373.1	40.373	161	95	1	20	180	403	562	3.46E-24	108	51.55
72	amcG	WP_148057932.1	41.463	123	69	1	41	163	17	136	1.08E-23	101	60.98
73	amcG	WP_102922765.1	43.478	138	78	0	22	159	10	147	2.03E-23	100	55.8
74	amcG	WP_223094296.1	44.242	165	75	4	28	177	5	167	3.06E-23	100	51.52
75	amcG	WP_032645024.1	52.252	111	52	1	67	177	1	110	1.94E-22	96.7	63.96
76	amcG	WP_042868955.1	35.976	164	103	2	22	184	14	176	7.44E-22	97.1	53.66
77	amcG	WP_009114554.1	34.337	166	107	2	20	184	12	176	8.20E-22	96.7	54.22
78	amcG	WP_190135286.1	41.722	151	86	2	27	177	20	168	1.12E-21	97.4	50.99
79	amcG	WP_084903002.1	38.776	147	89	1	31	177	23	168	2.27E-21	96.7	51.7
80	amcG	WP_142264107.1	38.621	145	87	1	36	178	27	171	5.36E-21	94.7	53.79
81	amcG	WP_131740835.1	39.716	141	85	0	22	162	12	152	2.82E-20	92.8	51.77
82	amcG	WP_233865706.1	42.857	147	84	0	20	166	403	549	3.42E-20	97.8	55.1
83	amcG	MBI3688123.1	43.046	151	82	2	30	177	18	167	4.11E-20	93.2	56.29
84	amcG	WP_086771546.1	34.591	159	102	1	22	178	13	171	6.81E-20	92	51.57
85	amcG	WP_219528372.1	40.397	151	89	1	32	182	20	169	2.18E-19	92	53.64
86	amcG	WP_052408053.1	37.41	139	83	2	39	177	15	149	2.50E-19	89.7	53.96
87	amcG	WP_218064164.1	32.624	141	91	2	37	177	15	151	5.28E-19	89.4	53.9
88	amcG	OLT10694.1	43.529	170	93	2	14	181	446	614	2.15E-18	92.4	55.29

89	amcG	WP_201843267.1	40.132	152	88	2	36	184	27	178	3.59E-18	87.4	54.61
90	amcG	WP_052209145.1	34.4	125	79	1	38	162	13	134	1.30E-17	85.9	53.6
91	amcG	NVN10070.1	31.111	135	90	1	43	177	1	132	1.71E-17	85.1	52.59
92	amcG	WP_189938222.1	37.931	145	88	1	36	178	27	171	2.62E-17	85.1	53.79
93	amcG	MCC3772573.1	44.545	110	60	1	75	184	1	109	2.63E-16	80.5	55.45
94	amcG	WP_162296926.1	31.724	145	95	2	34	177	9	150	2.01E-15	80.1	51.03
95	amcG	WP_190125360.1	37.762	143	85	1	39	177	37	179	2.54E-15	80.1	46.85
96	amcG	WP_217809648.1	40.594	101	59	1	77	177	10	109	1.09E-13	73.9	56.44
97	amcG	WP_116025564.1	35.338	133	83	2	42	174	19	148	1.37E-13	74.7	47.37
98	amcG	MBS0266747.1	31.875	160	108	1	24	182	17	176	2.32E-13	75.1	45.62
99	amcG	WP_143238584.1	33.333	147	94	3	33	177	2	146	2.81E-13	74.3	53.74
100	amcG	WP_096724586.1	31.387	137	91	2	41	177	17	150	7.60E-13	73.6	52.55

Detailed information on each BGC (e.g. species, homology to amcG)
ordered from BGCs containing AmcG with the highest compatibility (score)
RID: 90G2HUXE016
Job Title:Protein Sequence
Program: BLASTP
Database: nr_clustered(experimental) clustered nr
Query #1: Query ID: lcl|Query_57269 Length: 184
Clusters producing significant alignments:
Cluster: QCX41961.1 AmcG [Streptomyces novoguineensis]
Num Members: 1
Num Taxa: 1
Scientific Name: Streptomyces novoguineensis
Common Name : high GC Gram+
Taxid: 2586640
Highest Bit Score: 367
Total Bit Score: 367
Percent Coverage: 100%
Evalue: 3e-128
Percent Identity: 100.00%
Accession Length: 184
1 cluster member(s):
Accession Scientific Common Taxid
QCX41961.1 Streptomyces novoguineensis high GC Gram+ 2586640

Cluster: WP_189561794.1 coronafacic acid synthetase [Streptomyces pilosus]
Num Members: 1
Num Taxa: 1
Scientific Name: Streptomyces pilosus
Common Name : high GC Gram+
Taxid: 28893
Highest Bit Score: 327
Total Bit Score: 327
Percent Coverage: 98%
Evalue: 4e-112
Percent Identity: 89.01%
Accession Length: 197
1 cluster member(s):
Accession Scientific Common Taxid
WP_189561794.1 Streptomyces pilosus high GC Gram+ 28893
Cluster: WP_182468098.1 coronafacic acid synthetase [Streptomyces sp. GMR22]
Num Members: 7
Num Taxa: 6
Scientific Name: Streptomyces
Common Name : high G+C Gram-positive bacteria
Taxid: 1883
Highest Bit Score: 308
Total Bit Score: 308
Percent Coverage: 98%

Evalue: 5e-105
Percent Identity: 84.62%
Accession Length: 197
7 cluster member(s):
Accession Scientific Common Taxid
WP_182468098.1 Streptomyces sp. GMR22 high GC Gram+ 2759524
WP_059143060.1 Streptomyces high G+C Gram-positive bact... 1883
WP_161561932.1 Streptomyces antimycoticus high GC Gram+ 68175
WP_162003831.1 Streptomyces antimycoticus high GC Gram+ 68175
WP_191065350.1 Streptomyces sp. 5-10 high GC Gram+ 878925
WP_208652832.1 Streptomyces violaceusniger... high G+C Gram-positive bact... 2839105
WP_216341501.1 Streptomyces sp. 4503 high GC Gram+ 2842201
Cluster: MBB4673076.1 hypothetical protein [Streptomyces haliclonae]
Num Members: 1
Num Taxa: 1
Scientific Name: Streptomyces haliclonae
Common Name : high GC Gram+
Taxid: 582855
Highest Bit Score: 292
Total Bit Score: 292
Percent Coverage: 97%
Evalue: 3e-98
Percent Identity: 82.22%
Accession Length: 202

1 cluster member(s):
Accession Scientific Common Taxid
MBB4673076.1 Streptomyces haliclonae high GC Gram+ 582855
Cluster: WP_162795002.1 coronafacic acid synthetase [Nonomuraea lactucae]
Num Members: 1
Num Taxa: 1
Scientific Name: Nonomuraea lactucae
Common Name : high G+C Gram-positive bacteria
Taxid: 2249762
Highest Bit Score: 192
Total Bit Score: 192
Percent Coverage: 86%
Evalue: 2e-59
Percent Identity: 61.01%
Accession Length: 173
1 cluster member(s):
Accession Scientific Common Taxid
WP_162795002.1 Nonomuraea lactucae high G+C Gram-positive bact... 2249762
Cluster: WP_111167667.1 coronafacic acid synthetase [Spongiactinospora gelatinilytica]
Num Members: 1
Num Taxa: 1
Scientific Name: Spongiactinospora gelatinilytica
Common Name : high G+C Gram-positive bacteria

Taxid: 2666298
Highest Bit Score: 176
Total Bit Score: 176
Percent Coverage: 85%
Evalue: 1e-52
Percent Identity: 61.39%
Accession Length: 179
1 cluster member(s):
Accession Scientific Common Taxid
WP_111167667.1 Spongiactinospora gelatinil... high G+C Gram-positive bact... 2666298
Cluster: WP_113985346.1 coronafacic acid synthetase [Spongiactinospora rosea]
Num Members: 1
Num Taxa: 1
Scientific Name: Spongiactinospora rosea
Common Name : high GC Gram+
Taxid: 2248750
Highest Bit Score: 175
Total Bit Score: 175
Percent Coverage: 89%
Evalue: 1e-52
Percent Identity: 60.37%
Accession Length: 175
1 cluster member(s):
Accession Scientific Common Taxid

WP_113985346.1 Spongiactinospora rosea high GC Gram+ 2248750
Cluster: WP_209647138.1 hypothetical protein [Kibdelosporangium banguiense]
Num Members: 1
Num Taxa: 1
Scientific Name: Kibdelosporangium banguiense
Common Name : high G+C Gram-positive bacteria
Taxid: 1365924
Highest Bit Score: 160
Total Bit Score: 160
Percent Coverage: 86%
Evalue: 1e-46
Percent Identity: 51.57%
Accession Length: 173
1 cluster member(s):
Accession Scientific Common Taxid
WP_209647138.1 Kibdelosporangium banguiense high G+C Gram-positive bact... 1365924
Cluster: WP_052479233.1 hypothetical protein [Kibdelosporangium sp. MJ126-NF4]
Num Members: 1
Num Taxa: 1
Scientific Name: Kibdelosporangium sp. MJ126-NF4
Common Name : high GC Gram+
Taxid: 703222
Highest Bit Score: 153

Total Bit Score: 153
Percent Coverage: 88%
Evalue: 5e-44
Percent Identity: 50.92%
Accession Length: 171
1 cluster member(s):
Accession Scientific Common Taxid
WP_052479233.1 Kibdelosporangium sp. MJ126... high GC Gram+ 703222
Cluster: WP_105967719.1 coronafacic acid synthetase [Streptomyces geranii]
Num Members: 1
Num Taxa: 1
Scientific Name: Streptomyces geranii
Common Name : high GC Gram+
Taxid: 2058923
Highest Bit Score: 143
Total Bit Score: 143
Percent Coverage: 92%
Evalue: 6e-40
Percent Identity: 48.28%
Accession Length: 179
1 cluster member(s):
Accession Scientific Common Taxid
WP_105967719.1 Streptomyces geranii high GC Gram+ 2058923

Cluster: WP_184795263.1 hypothetical protein [Kribbella italica]
Num Members: 1
Num Taxa: 1
Scientific Name: Kribbella italica
Common Name : high G+C Gram-positive bacteria
Taxid: 1540520
Highest Bit Score: 140
Total Bit Score: 140
Percent Coverage: 86%
Evalue: 3e-39
Percent Identity: 49.06%
Accession Length: 166
1 cluster member(s):
Accession Scientific Common Taxid
WP_184795263.1 Kribbella italica high G+C Gram-positive bact... 1540520
Cluster: MBV9022849.1 coronafacic acid synthetase [Streptomycetaceae bacterium]
Num Members: 1
Num Taxa: 1
Scientific Name: Streptomycetaceae bacterium
Common Name : high GC Gram+
Taxid: 2021369
Highest Bit Score: 140
Total Bit Score: 140
Percent Coverage: 87%

Evalue: 5e-39
Percent Identity: 48.77%
Accession Length: 176
1 cluster member(s):
Accession Scientific Common Taxid
MBV9022849.1 Streptomycetaceae bacterium high GC Gram+ 2021369
Cluster: WP_182471800.1 coronafacic acid synthetase [Streptomyces sp. GMR22]
Num Members: 1
Num Taxa: 1
Scientific Name: Streptomyces sp. GMR22
Common Name : high GC Gram+
Taxid: 2759524
Highest Bit Score: 140
Total Bit Score: 140
Percent Coverage: 74%
Evalue: 7e-39
Percent Identity: 51.82%
Accession Length: 188
1 cluster member(s):
Accession Scientific Common Taxid
WP_182471800.1 Streptomyces sp. GMR22 high GC Gram+ 2759524
Cluster: WP_024802760.1 hypothetical protein [Nocardia sp. BMG51109]

Num Members: 1
Num Taxa: 1
Scientific Name: Nocardia sp. BMG51109
Common Name : high GC Gram+
Taxid: 1056816
Highest Bit Score: 140
Total Bit Score: 140
Percent Coverage: 86%
Evalue: 1e-38
Percent Identity: 47.83%
Accession Length: 183
1 cluster member(s):
Accession Scientific Common Taxid
WP_024802760.1 Nocardia sp. BMG51109 high GC Gram+ 1056816
Cluster: WP_055634613.1 hypothetical protein [Streptomyces griseoruber]
Num Members: 1
Num Taxa: 1
Scientific Name: Streptomyces griseoruber
Common Name : high GC Gram+
Taxid: 1943
Highest Bit Score: 137
Total Bit Score: 137
Percent Coverage: 88%
Evalue: 1e-37
Percent Identity: 47.62%

Accession Length: 182
1 cluster member(s):
Accession Scientific Common Taxid
WP_055634613.1 Streptomyces griseoruber high GC Gram+ 1943
Cluster: WP_169393912.1 hypothetical protein [Pseudonocardia xinjiangensis]
Num Members: 1
Num Taxa: 1
Scientific Name: Pseudonocardia xinjiangensis
Common Name : high G+C Gram-positive bacteria
Taxid: 75289
Highest Bit Score: 136
Total Bit Score: 136
Percent Coverage: 78%
Evalue: 3e-37
Percent Identity: 48.61%
Accession Length: 174
1 cluster member(s):
Accession Scientific Common Taxid
WP_169393912.1 Pseudonocardia xinjiangensis high G+C Gram-positive bact... 75289
Cluster: WP_086756671.1 coronafacic acid synthetase [Streptomyces]
Num Members: 1
Num Taxa: 1

Scientific Name: Streptomyces
Common Name : high G+C Gram-positive bacteria
Taxid: 1883
Highest Bit Score: 135
Total Bit Score: 135
Percent Coverage: 98%
Evalue: 5e-37
Percent Identity: 46.84%
Accession Length: 182
1 cluster member(s):
Accession Scientific Common Taxid
WP_086756671.1 Streptomyces high G+C Gram-positive bact... 1883
Cluster: WP_059080167.1 hypothetical protein [Streptomyces scabiei]
Num Members: 7
Num Taxa: 6
Scientific Name: Streptomycetaceae
Common Name : high G+C Gram-positive bacteria
Taxid: 2062
Highest Bit Score: 133
Total Bit Score: 133
Percent Coverage: 87%
Evalue: 4e-36
Percent Identity: 46.91%
Accession Length: 175

7 cluster member(s):
Accession Scientific Common Taxid
WP_059080167.1 Streptomyces scabiei high GC Gram+ 1930
WP_013005373.1 Streptomyces high G+C Gram-positive bact... 1883
WP_035840064.1 Kitasatospora azatica high GC Gram+ 58347
WP_053745358.1 Streptomyces sp. NRRL WC-3618 high GC Gram+ 1519490
WP_055532734.1 Streptomyces graminilatus high GC Gram+ 1464070
WP_060907552.1 Streptomyces high G+C Gram-positive bact... 1883
WP_099965308.1 Streptomyces sp. JV178 high GC Gram+ 858632
Cluster: WP_098276132.1 coronafacic acid synthetase [Bacillus cereus]
Num Members: 5
Num Taxa: 5
Scientific Name: Bacillus
Common Name : Bacillus rRNA group 1
Taxid: 1386
Highest Bit Score: 133
Total Bit Score: 133
Percent Coverage: 78%
Evalue: 5e-36
Percent Identity: 41.67%
Accession Length: 180
5 cluster member(s):
Accession Scientific Common Taxid
WP_098276132.1 Bacillus cereus firmicutes 1396
PGK36905.1 Bacillus anthracis firmicutes 1392

WP_000932275.1	Bacillus cereus group	firmicutes	86661
WP_099684512.1	Bacillus fungorum	firmicutes	2039284
WP_213745700.1	Bacillus toyonensis	firmicutes	155322
Cluster:	WP_242226323.1 coronafacic acid synthetase [Bacillus cereus group sp. BfR-BA-01358]		
Num Members:	3		
Num Taxa:	3		
Scientific Name:	Bacillus cereus group		
Common Name :	firmicutes		
Taxid:	86661		
Highest Bit Score:	130		
Total Bit Score:	130		
Percent Coverage:	69%		
Evalue:	4e-35		
Percent Identity:	44.88%		
Accession Length:	149		
3 cluster member(s):			
Accession	Scientific	Common	Taxid
WP_242226323.1	Bacillus cereus group sp. B...	firmicutes	2920320
EEL46991.1	Bacillus cereus Rock3-42	firmicutes	526985
KLV20568.1	Bacillus anthracis	firmicutes	1392
Cluster:	WP_076986735.1 hypothetical protein [Actinosynnema sp. ALI-1.44]		
Num Members:	1		
Num Taxa:	1		

Scientific Name: Actinosynnema sp. ALI-1.44
Common Name : high GC Gram+
Taxid: 1933779
Highest Bit Score: 128
Total Bit Score: 128
Percent Coverage: 85%
Evalue: 3e-34
Percent Identity: 47.77%
Accession Length: 156
1 cluster member(s):
Accession Scientific Common Taxid
WP_076986735.1 Actinosynnema sp. ALI-1.44 high GC Gram+ 1933779
Cluster: MCE7004706.1 hypothetical protein [Kibdelosporangium philippinense]
Num Members: 1
Num Taxa: 1
Scientific Name: Kibdelosporangium philippinense
Common Name : high GC Gram+
Taxid: 211113
Highest Bit Score: 127
Total Bit Score: 127
Percent Coverage: 77%
Evalue: 8e-34
Percent Identity: 47.89%
Accession Length: 190

1 cluster member(s):
Accession Scientific Common Taxid
MCE7004706.1 Kibdelosporangium philippin... high GC Gram+ 211113
Cluster: WP_138200933.1 coronafacic acid synthetase [unclassified Nonomuraea]
Num Members: 1
Num Taxa: 1
Scientific Name: unclassified Nonomuraea
Common Name : high GC Gram+
Taxid: 2593643
Highest Bit Score: 127
Total Bit Score: 127
Percent Coverage: 76%
Evalue: 2e-33
Percent Identity: 47.52%
Accession Length: 190
1 cluster member(s):
Accession Scientific Common Taxid
WP_138200933.1 unclassified Nonomuraea high GC Gram+ 2593643
Cluster: WP_053761438.1 hypothetical protein [Streptomyces]
Num Members: 1
Num Taxa: 1
Scientific Name: Streptomyces
Common Name : high G+C Gram-positive bacteria

Taxid: 1883
Highest Bit Score: 127
Total Bit Score: 127
Percent Coverage: 90%
Evalue: 2e-33
Percent Identity: 44.31%
Accession Length: 191
1 cluster member(s):
Accession Scientific Common Taxid
WP_053761438.1 Streptomyces high G+C Gram-positive bact... 1883
Cluster: WP_052422984.1 hypothetical protein [Nonomuraea candida]
Num Members: 1
Num Taxa: 1
Scientific Name: Nonomuraea candida
Common Name : high GC Gram+
Taxid: 359159
Highest Bit Score: 126
Total Bit Score: 126
Percent Coverage: 79%
Evalue: 3e-33
Percent Identity: 46.58%
Accession Length: 190
1 cluster member(s):
Accession Scientific Common Taxid

WP_052422984.1	Nonomuraea candida	high GC Gram+	359159
Cluster:	WP_058767951.1 hypothetical protein [Pseudomonas psychrotolerans]		
Num Members:	2		
Num Taxa:	1		
Scientific Name:	Pseudomonas psychrotolerans		
Common Name :	g-proteobacteria		
Taxid:	237610		
Highest Bit Score:	125		
Total Bit Score:	125		
Percent Coverage:	77%		
Evalue:	6e-33		
Percent Identity:	51.41%		
Accession Length:	177		
2 cluster member(s):			
Accession	Scientific	Common	Taxid
WP_058767951.1	Pseudomonas psychrotolerans	g-proteobacteria	237610
WP_058777210.1	Pseudomonas psychrotolerans	g-proteobacteria	237610
Cluster:	WP_069846947.1 hypothetical protein [Actinoalloteichus hymeniacidonis]		
Num Members:	1		
Num Taxa:	1		
Scientific Name:	Actinoalloteichus hymeniacidonis		
Common Name :	high GC Gram+		
Taxid:	340345		

Highest Bit Score: 124
Total Bit Score: 124
Percent Coverage: 79%
Evalue: 1e-32
Percent Identity: 45.89%
Accession Length: 190
1 cluster member(s):
Accession Scientific Common Taxid
WP_069846947.1 Actinoalloteichus hymeniaci... high GC Gram+ 340345
Cluster: WP_184622999.1 coronafacic acid synthetase [Xanthomonas arboricola]
Num Members: 2
Num Taxa: 2
Scientific Name: Xanthomonas
Common Name : g-proteobacteria
Taxid: 338
Highest Bit Score: 123
Total Bit Score: 123
Percent Coverage: 77%
Evalue: 4e-32
Percent Identity: 50.70%
Accession Length: 177
2 cluster member(s):
Accession Scientific Common Taxid
WP_184622999.1 Xanthomonas arboricola g-proteobacteria 56448

WP_184408479.1	Xanthomonas campestris	g-proteobacteria	339
Cluster:	WP_164386746.1 hypothetical protein [Streptomyces sp. OM5714]		
Num Members:	2		
Num Taxa:	1		
Scientific Name:	Streptomyces sp. OM5714		
Common Name :	high GC Gram+		
Taxid:	2602736		
Highest Bit Score:	123		
Total Bit Score:	123		
Percent Coverage:	77%		
Evalue:	5e-32		
Percent Identity:	48.32%		
Accession Length:	190		
2 cluster member(s):			
Accession	Scientific	Common	Taxid
WP_164386746.1	Streptomyces sp. OM5714	high GC Gram+	2602736
KAF2774996.1	Streptomyces sp. OM5714	high GC Gram+	2602736
Cluster:	WP_165956521.1 coronafacic acid synthetase component [Kribbella antibiotica]		
Num Members:	1		
Num Taxa:	1		
Scientific Name:	Kribbella antibiotica		
Common Name :	high G+C Gram-positive bacteria		
Taxid:	190195		

Highest Bit Score: 120
Total Bit Score: 120
Percent Coverage: 75%
Evalue: 2e-31
Percent Identity: 48.20%
Accession Length: 153
1 cluster member(s):
Accession Scientific Common Taxid
WP_165956521.1 Kribbella antibiotica high G+C Gram-positive bact... 190195
Cluster: RLV10181.1 polyketide synthase [Streptomyces griseocarneus]
Num Members: 1
Num Taxa: 1
Scientific Name: Streptomyces griseocarneus
Common Name : high GC Gram+
Taxid: 51201
Highest Bit Score: 120
Total Bit Score: 120
Percent Coverage: 78%
Evalue: 4e-31
Percent Identity: 47.92%
Accession Length: 171
1 cluster member(s):
Accession Scientific Common Taxid
RLV10181.1 Streptomyces griseocarneus high GC Gram+ 51201

Cluster: WP_171079069.1 polyketide synthase [Streptomyces]
Num Members: 1
Num Taxa: 1
Scientific Name: Streptomyces
Common Name : high G+C Gram-positive bacteria
Taxid: 1883
Highest Bit Score: 119
Total Bit Score: 119
Percent Coverage: 95%
Evalue: 8e-31
Percent Identity: 47.16%
Accession Length: 172
1 cluster member(s):
Accession Scientific Common Taxid
WP_171079069.1 Streptomyces morookaense high G+C Gram-positive bact... 1970
Cluster: KPB94666.1 Coronafacic acid synthetase component [Pseudomonas syringae pv. maculicola]
Num Members: 3
Num Taxa: 1
Scientific Name: Pseudomonas syringae pv. maculicola
Common Name : g-proteobacteria
Taxid: 59511
Highest Bit Score: 119
Total Bit Score: 119

Percent Coverage: 73%
Evalue: 1e-30
Percent Identity: 51.47%
Accession Length: 158
3 cluster member(s):
Accession Scientific Common Taxid
KPB94666.1 Pseudomonas syringae pv. ma... g-proteobacteria 59511
KPB71907.1 Pseudomonas syringae pv. ma... g-proteobacteria 59511
KPB77244.1 Pseudomonas syringae pv. ma... g-proteobacteria 59511
Cluster: WP_070024088.1 hypothetical protein [Streptomyces sp. F-1]
Num Members: 3
Num Taxa: 3
Scientific Name: Streptomyces
Common Name : high G+C Gram-positive bacteria
Taxid: 1883
Highest Bit Score: 120
Total Bit Score: 120
Percent Coverage: 86%
Evalue: 1e-30
Percent Identity: 47.88%
Accession Length: 201
3 cluster member(s):
Accession Scientific Common Taxid
WP_070024088.1 Streptomyces sp. F-1 high GC Gram+ 463642

WP_074992635.1	Streptomyces misionensis	high GC Gram+	67331
WP_180330491.1	Streptomyces sp. NEAU-sy36	high GC Gram+	2751189
Cluster:	WP_171675246.1 coronafacic acid synthetase component [Kribbella sandramycini]		
Num Members:	1		
Num Taxa:	1		
Scientific Name:	Kribbella sandramycini		
Common Name :	high G+C Gram-positive bacteria		
Taxid:	60450		
Highest Bit Score:	117		
Total Bit Score:	117		
Percent Coverage:	71%		
Evalue:	2e-30		
Percent Identity:	48.48%		
Accession Length:	137		
1 cluster member(s):			
Accession	Scientific	Common	Taxid
WP_171675246.1	Kribbella sandramycini	high G+C Gram-positive bact...	60450
Cluster:	WP_054990124.1 hypothetical protein [Pseudomonas syringae group]		
Num Members:	1		
Num Taxa:	1		
Scientific Name:	Pseudomonas syringae group		
Common Name :	g-proteobacteria		
Taxid:	136849		

Highest Bit Score: 118
Total Bit Score: 118
Percent Coverage: 73%
Evalue: 3e-30
Percent Identity: 51.47%
Accession Length: 177
1 cluster member(s):
Accession Scientific Common Taxid
WP_054990124.1 Pseudomonas syringae group g-proteobacteria 136849
Cluster: WP_177227589.1 hypothetical protein [Nonomuraea pusilla]
Num Members: 2
Num Taxa: 1
Scientific Name: Nonomuraea pusilla
Common Name : high GC Gram+
Taxid: 46177
Highest Bit Score: 125
Total Bit Score: 125
Percent Coverage: 85%
Evalue: 4e-30
Percent Identity: 49.38%
Accession Length: 580
2 cluster member(s):
Accession Scientific Common Taxid
WP_177227589.1 Nonomuraea pusilla high GC Gram+ 46177

WP_176573594.1	Nonomuraea pusilla	high GC Gram+	46177
Cluster:	WP_094109901.1 coronafacic acid synthetase [Lonsdalea iberica]		
Num Members:	2		
Num Taxa:	1		
Scientific Name:	Lonsdalea iberica		
Common Name :	enterobacteria		
Taxid:	1082703		
Highest Bit Score:	117		
Total Bit Score:	117		
Percent Coverage:	86%		
Evalue:	6e-30		
Percent Identity:	41.88%		
Accession Length:	178		
2 cluster member(s):			
Accession	Scientific	Common	Taxid
WP_094109901.1	Lonsdalea iberica	enterobacteria	1082703
WP_094101872.1	Lonsdalea iberica	enterobacteria	1082703
Cluster:	WP_191140370.1 coronafacic acid synthetase [unclassified Hazenella]		
Num Members:	1		
Num Taxa:	1		
Scientific Name:	unclassified Hazenella		
Common Name :	firmicutes		
Taxid:	2618610		

Highest Bit Score: 117
Total Bit Score: 117
Percent Coverage: 86%
Evalue: 9e-30
Percent Identity: 34.38%
Accession Length: 197
1 cluster member(s):
Accession Scientific Common Taxid
WP_191140370.1 unclassified Hazenella firmicutes 2618610
Cluster: WP_051616280.1 hypothetical protein [Lonsdalea quercina]
Num Members: 1
Num Taxa: 1
Scientific Name: Lonsdalea quercina
Common Name : enterobacteria
Taxid: 71657
Highest Bit Score: 117
Total Bit Score: 117
Percent Coverage: 84%
Evalue: 1e-29
Percent Identity: 42.31%
Accession Length: 186
1 cluster member(s):
Accession Scientific Common Taxid
WP_051616280.1 Lonsdalea quercina enterobacteria 71657

Cluster: WP_085653152.1 coronafacic acid synthetase [Lonsdalea britannica]
Num Members: 1
Num Taxa: 1
Scientific Name: Lonsdalea britannica
Common Name : enterobacteria
Taxid: 1082704
Highest Bit Score: 116
Total Bit Score: 116
Percent Coverage: 87%
Evalue: 2e-29
Percent Identity: 42.59%
Accession Length: 178
1 cluster member(s):
Accession Scientific Common Taxid
WP_085653152.1 Lonsdalea britannica enterobacteria 1082704
Cluster: WP_066982330.1 hypothetical protein [Streptomyces sp. NRRL F-4489]
Num Members: 1
Num Taxa: 1
Scientific Name: Streptomyces sp. NRRL F-4489
Common Name : high GC Gram+
Taxid: 1609095
Highest Bit Score: 116
Total Bit Score: 116

Percent Coverage: 94%
Evalue: 3e-29
Percent Identity: 40.80%
Accession Length: 200
1 cluster member(s):
Accession   Scientific   Common   Taxid
WP_066982330.1   Streptomyces sp. NRRL F-4489   high GC Gram+   1609095
Cluster: TMM37893.1 polyketide synthase [Actinobacteria bacterium]
Num Members: 1
Num Taxa: 1
Scientific Name: Actinobacteria
Common Name :   actinobacteria
Taxid: 201174
Highest Bit Score: 115
Total Bit Score: 115
Percent Coverage: 73%
Evalue: 4e-29
Percent Identity: 47.79%
Accession Length: 194
1 cluster member(s):
Accession   Scientific   Common   Taxid
TMM37893.1   Actinomycetia bacterium   high GC Gram+   2900548

Cluster: KPW22679.1 Coronafacic acid beta-ketoacyl synthetase component [Pseudomonas amygdali pv. aesculi]
Num Members: 1
Num Taxa: 1
Scientific Name: Pseudomonas amygdali pv. aesculi
Common Name : g-proteobacteria
Taxid: 251722
Highest Bit Score: 121
Total Bit Score: 121
Percent Coverage: 73%
Evalue: 2e-28
Percent Identity: 52.21%
Accession Length: 601
1 cluster member(s):
Accession Scientific Common Taxid
KPW22679.1 Pseudomonas amygdali pv. ae... g-proteobacteria 251722
Cluster: WP_136167060.1 coronafacic acid synthetase [Brenneria sp. CFCC 11842]
Num Members: 1
Num Taxa: 1
Scientific Name: Brenneria sp. CFCC 11842
Common Name : enterobacteria
Taxid: 2173106
Highest Bit Score: 113
Total Bit Score: 113
Percent Coverage: 88%
Evalue: 3e-28

Percent Identity: 40.24%
Accession Length: 178
1 cluster member(s):
Accession Scientific Common Taxid
WP_136167060.1 Brenneria sp. CFCC 11842 enterobacteria 2173106
Cluster: WP_228321346.1 hypothetical protein [Xanthomonas campestris]
Num Members: 1
Num Taxa: 1
Scientific Name: Xanthomonas campestris
Common Name : g-proteobacteria
Taxid: 339
Highest Bit Score: 112
Total Bit Score: 112
Percent Coverage: 87%
Evalue: 4e-28
Percent Identity: 46.30%
Accession Length: 177
1 cluster member(s):
Accession Scientific Common Taxid
WP_228321346.1 Xanthomonas campestris g-proteobacteria 339
Cluster: WP_132261363.1 hypothetical protein [Micromonospora sp. KC721]
Num Members: 2

Num Taxa: 2
Scientific Name: unclassified Micromonospora
Common Name : high GC Gram+
Taxid: 2617518
Highest Bit Score: 112
Total Bit Score: 112
Percent Coverage: 79%
Evalue: 5e-28
Percent Identity: 44.90%
Accession Length: 171
2 cluster member(s):

Accession	Scientific	Common	Taxid
WP_132261363.1	Micromonospora sp. KC721	high GC Gram+	2530380
WP_165944254.1	Micromonospora sp. KC213	high GC Gram+	2530378

Cluster: WP_131124666.1 polyketide synthase [Streptomyces kasugaensis]
Num Members: 5
Num Taxa: 3
Scientific Name: Streptomyces
Common Name : high G+C Gram-positive bacteria
Taxid: 1883
Highest Bit Score: 112
Total Bit Score: 112
Percent Coverage: 78%
Evalue: 5e-28
Percent Identity: 45.14%

Accession Length: 172
5 cluster member(s):
Accession Scientific Common Taxid
WP_131124666.1 Streptomyces kasugaensis high GC Gram+ 1946
MYU52879.1 Streptomyces sp. SID7805 high GC Gram+ 2690328
WP_094792103.1 Streptomyces kasugaensis high GC Gram+ 1946
WP_205379488.1 Streptomyces sp. SID7805 high GC Gram+ 2690328
WP_227046003.1 Streptomyces sp. SF28 high GC Gram+ 2884812
Cluster: WP_068798146.1 hypothetical protein [Pseudonocardia sp. HH130630-07]
Num Members: 1
Num Taxa: 1
Scientific Name: Pseudonocardia sp. HH130630-07
Common Name : high GC Gram+
Taxid: 1690815
Highest Bit Score: 111
Total Bit Score: 111
Percent Coverage: 75%
Evalue: 2e-27
Percent Identity: 50.72%
Accession Length: 182
1 cluster member(s):
Accession Scientific Common Taxid
WP_068798146.1 Pseudonocardia sp. HH130630-07 high GC Gram+ 1690815

Cluster: WP_172289556.1 coronafacic acid synthetase [Brenneria sp. hezel4-2-4]
Num Members: 2
Num Taxa: 2
Scientific Name: unclassified Brenneria
Common Name : enterobacteria
Taxid: 2634434
Highest Bit Score: 110
Total Bit Score: 110
Percent Coverage: 65%
Evalue: 4e-27
Percent Identity: 45.45%
Accession Length: 178
2 cluster member(s):
Accession Scientific Common Taxid
WP_172289556.1 Brenneria sp. hezel4-2-4 enterobacteria 2737661
WP_199377647.1 Brenneria sp. L3-3C-1 enterobacteria 2799634
Cluster: WP_053666612.1 hypothetical protein [Streptomyces sp. MMG1121]
Num Members: 1
Num Taxa: 1
Scientific Name: Streptomyces sp. MMG1121
Common Name : high GC Gram+
Taxid: 1415544
Highest Bit Score: 110
Total Bit Score: 110

Percent Coverage: 85%
Evalue: 5e-27
Percent Identity: 44.94%
Accession Length: 200
1 cluster member(s):
Accession Scientific Common Taxid
WP_053666612.1 Streptomyces sp. MMG1121 high GC Gram+ 1415544
Cluster: WP_100602528.1 polyketide synthase [Streptomyces sp. CB02959]
Num Members: 1
Num Taxa: 1
Scientific Name: Streptomyces sp. CB02959
Common Name : high GC Gram+
Taxid: 2020330
Highest Bit Score: 109
Total Bit Score: 109
Percent Coverage: 92%
Evalue: 1e-26
Percent Identity: 40.94%
Accession Length: 172
1 cluster member(s):
Accession Scientific Common Taxid
WP_100602528.1 Streptomyces sp. CB02959 high GC Gram+ 2020330

Cluster: WP_038524297.1 polyketide synthase [Streptomyces albulus]
Num Members: 4
Num Taxa: 2
Scientific Name: Streptomyces albulus
Common Name : high GC Gram+
Taxid: 68570
Highest Bit Score: 108
Total Bit Score: 108
Percent Coverage: 78%
Evalue: 1e-26
Percent Identity: 46.21%
Accession Length: 172
4 cluster member(s):

Accession	Scientific	Common	Taxid
WP_038524297.1	Streptomyces albulus	high GC Gram+	68570
AKA07723.1	Streptomyces albulus ZPM	high GC Gram+	1434306
WP_016578535.1	Streptomyces albulus	high GC Gram+	68570
WP_233660980.1	Streptomyces albulus	high GC Gram+	68570

Cluster: WP_018537882.1 hypothetical protein [unclassified Streptomyces]
Num Members: 1
Num Taxa: 1
Scientific Name: unclassified Streptomyces
Common Name : high G+C Gram-positive bacteria
Taxid: 2593676
Highest Bit Score: 108

Total Bit Score: 108
Percent Coverage: 94%
Evalue: 1e-26
Percent Identity: 40.46%
Accession Length: 172
1 cluster member(s):
Accession Scientific Common Taxid
WP_018537882.1 unclassified Streptomyces high G+C Gram-positive bact... 2593676
Cluster: WP_211763030.1 hypothetical protein [Kutzneria sp. CA-103260]
Num Members: 1
Num Taxa: 1
Scientific Name: Kutzneria sp. CA-103260
Common Name : high GC Gram+
Taxid: 2802641
Highest Bit Score: 108
Total Bit Score: 108
Percent Coverage: 74%
Evalue: 2e-26
Percent Identity: 47.45%
Accession Length: 162
1 cluster member(s):
Accession Scientific Common Taxid
WP_211763030.1 Kutzneria sp. CA-103260 high GC Gram+ 2802641

Cluster: WP_027704559.1 hypothetical protein [Zymobacter palmae]
Num Members: 1
Num Taxa: 1
Scientific Name: Zymobacter palmae
Common Name : g-proteobacteria
Taxid: 33074
Highest Bit Score: 108
Total Bit Score: 108
Percent Coverage: 89%
Evalue: 2e-26
Percent Identity: 40.96%
Accession Length: 178
1 cluster member(s):
Accession Scientific Common Taxid
WP_027704559.1 Zymobacter palmae g-proteobacteria 33074
Cluster: WP_039313139.1 hypothetical protein [Pectobacterium]
Num Members: 6
Num Taxa: 6
Scientific Name: Pectobacteriaceae
Common Name : enterobacteria
Taxid: 1903410
Highest Bit Score: 108
Total Bit Score: 108
Percent Coverage: 88%

Evalue: 2e-26
Percent Identity: 37.20%
Accession Length: 177
6 cluster member(s):
Accession Scientific Common Taxid
WP_039313139.1 Pectobacterium enterobacteria 122277
WP_011092224.1 Pectobacterium atrosepticum enterobacteria 29471
WP_039308623.1 Pectobacterium betavasculorum enterobacteria 55207
WP_039355766.1 Pectobacterium actinidiae enterobacteria 1507808
WP_071783818.1 Dickeya dadantii enterobacteria 204038
WP_205534958.1 Pectobacterium brasiliense enterobacteria 180957
Cluster: WP_052293700.1 hypothetical protein [Azospirillum sp. B510]
Num Members: 1
Num Taxa: 1
Scientific Name: Azospirillum sp. B510
Common Name : a-proteobacteria
Taxid: 137722
Highest Bit Score: 108
Total Bit Score: 108
Percent Coverage: 88%
Evalue: 3e-26
Percent Identity: 40.00%
Accession Length: 176
1 cluster member(s):

Accession	Scientific	Common	Taxid
WP_052293700.1	Azospirillum sp. B510	a-proteobacteria	137722
Cluster:	WP_214636464.1 hypothetical protein [Nonomuraea sp. NEAU-A123]		
Num Members:	1		
Num Taxa:	1		
Scientific Name:	Nonomuraea sp. NEAU-A123		
Common Name :	high GC Gram+		
Taxid:	2839649		
Highest Bit Score:	108		
Total Bit Score:	108		
Percent Coverage:	82%		
Evalue:	6e-26		
Percent Identity:	41.72%		
Accession Length:	210		
1 cluster member(s):			
Accession	Scientific	Common	Taxid
WP_214636464.1	Nonomuraea sp. NEAU-A123	high GC Gram+	2839649
Cluster:	WP_161293594.1 polyketide synthase [Streptomyces sp. SID161]		
Num Members:	3		
Num Taxa:	3		
Scientific Name:	unclassified Streptomyces		
Common Name :	high G+C Gram-positive bacteria		
Taxid:	2593676		

Highest Bit Score: 107
Total Bit Score: 107
Percent Coverage: 76%
Evalue: 9e-26
Percent Identity: 45.00%
Accession Length: 188
3 cluster member(s):
Accession Scientific Common Taxid
WP_161293594.1 Streptomyces sp. SID161 high GC Gram+ 2690251
MYW16501.1 Streptomyces sp. SID2955 high GC Gram+ 2690257
WP_161334759.1 Streptomyces sp. SID486 high GC Gram+ 2690264
Cluster: WP_121174718.1 hypothetical protein [Streptomyces sp. 1114.5]
Num Members: 2
Num Taxa: 2
Scientific Name: unclassified Streptomyces
Common Name : high G+C Gram-positive bacteria
Taxid: 2593676
Highest Bit Score: 107
Total Bit Score: 107
Percent Coverage: 73%
Evalue: 1e-25
Percent Identity: 48.91%
Accession Length: 192
2 cluster member(s):

Accession	Scientific	Common	Taxid
WP_121174718.1	Streptomyces sp. 1114.5	high GC Gram+	1938830
WP_097237192.1	Streptomyces sp. 1331.2	high GC Gram+	1938835
Cluster:	WP_103963945.1 polyketide synthase [Nonomuraea solani]		
Num Members:	1		
Num Taxa:	1		
Scientific Name:	Nonomuraea solani		
Common Name :	high G+C Gram-positive bacteria		
Taxid:	1144553		
Highest Bit Score:	106		
Total Bit Score:	106		
Percent Coverage:	86%		
Evalue:	1e-25		
Percent Identity:	38.12%		
Accession Length:	166		
1 cluster member(s):			
Accession	Scientific	Common	Taxid
WP_103963945.1	Nonomuraea solani	high G+C Gram-positive bact...	1144553
Cluster:	WP_225447214.1 hypothetical protein [Streptacidiphilus sp. PB12-B1b]		
Num Members:	2		
Num Taxa:	1		
Scientific Name:	Streptacidiphilus sp. PB12-B1b		
Common Name :	high GC Gram+		

Taxid: 2705012
Highest Bit Score: 112
Total Bit Score: 112
Percent Coverage: 87%
Evalue: 2e-25
Percent Identity: 41.98%
Accession Length: 592
2 cluster member(s):
Accession Scientific Common Taxid
WP_225447214.1 Streptacidiphilus sp. PB12-B1b high GC Gram+ 2705012
QMU78299.1 Streptacidiphilus sp. PB12-B1b high GC Gram+ 2705012
Cluster: BAI73721.1 coronafacic acid synthetase component [Azospirillum sp. B510]
Num Members: 1
Num Taxa: 1
Scientific Name: Azospirillum sp. B510
Common Name : a-proteobacteria
Taxid: 137722
Highest Bit Score: 104
Total Bit Score: 104
Percent Coverage: 78%
Evalue: 5e-25
Percent Identity: 42.36%
Accession Length: 149
1 cluster member(s):

Accession	Scientific	Common	Taxid
BAI73721.1	Azospirillum sp. B510	a-proteobacteria	137722
Cluster:	WP_066984015.1 polyketide synthase [Streptomyces sp. NRRL F-4489]		
Num Members:	1		
Num Taxa:	1		
Scientific Name:	Streptomyces sp. NRRL F-4489		
Common Name :	high GC Gram+		
Taxid:	1609095		
Highest Bit Score:	105		
Total Bit Score:	105		
Percent Coverage:	78%		
Evalue:	5e-25		
Percent Identity:	45.52%		
Accession Length:	173		
1 cluster member(s):			
Accession	Scientific	Common	Taxid
WP_066984015.1	Streptomyces sp. NRRL F-4489	high GC Gram+	1609095
Cluster:	WP_063351990.1 hypothetical protein [Streptomyces sp. MJM8645]		
Num Members:	1		
Num Taxa:	1		
Scientific Name:	Streptomyces sp. MJM8645		
Common Name :	high GC Gram+		
Taxid:	1120523		

Highest Bit Score: 104
Total Bit Score: 104
Percent Coverage: 74%
Evalue: 8e-25
Percent Identity: 44.53%
Accession Length: 172
1 cluster member(s):
Accession Scientific Common Taxid
WP_063351990.1 Streptomyces sp. MJM8645 high GC Gram+ 1120523
Cluster: WP_240630143.1 coronafacic acid synthetase [Brenneria alni]
Num Members: 2
Num Taxa: 1
Scientific Name: Brenneria alni
Common Name : enterobacteria
Taxid: 71656
Highest Bit Score: 103
Total Bit Score: 103
Percent Coverage: 88%
Evalue: 2e-24
Percent Identity: 35.98%
Accession Length: 178
2 cluster member(s):
Accession Scientific Common Taxid
WP_240630143.1 Brenneria alni enterobacteria 71656

RLM21878.1	Brenneria alni	enterobacteria	71656
Cluster:	WP_189205086.1 hypothetical protein [Couchioplanes caeruleus]		
Num Members:	1		
Num Taxa:	1		
Scientific Name:	Couchioplanes caeruleus		
Common Name :	high GC Gram+		
Taxid:	56438		
Highest Bit Score:	103		
Total Bit Score:	103		
Percent Coverage:	87%		
Evalue:	2e-24		
Percent Identity:	45.34%		
Accession Length:	167		
1 cluster member(s):			
Accession	Scientific	Common	Taxid
WP_189205086.1	Couchioplanes caeruleus	high GC Gram+	56438
Cluster:	WP_089956931.1 hypothetical protein [Lentzea xinjiangensis]		
Num Members:	1		
Num Taxa:	1		
Scientific Name:	Lentzea xinjiangensis		
Common Name :	high GC Gram+		
Taxid:	402600		
Highest Bit Score:	103		

Total Bit Score: 103
Percent Coverage: 75%
Evalue: 3e-24
Percent Identity: 44.20%
Accession Length: 190
1 cluster member(s):
Accession Scientific Common Taxid
WP_089956931.1 Lentzea xinjiangensis high GC Gram+ 402600
Cluster: WP_208298038.1 hypothetical protein [Actinophytocola oryzae]
Num Members: 1
Num Taxa: 1
Scientific Name: Actinophytocola oryzae
Common Name : high G+C Gram-positive bacteria
Taxid: 502181
Highest Bit Score: 103
Total Bit Score: 103
Percent Coverage: 79%
Evalue: 3e-24
Percent Identity: 45.70%
Accession Length: 189
1 cluster member(s):
Accession Scientific Common Taxid
WP_208298038.1 Actinophytocola oryzae high G+C Gram-positive bact... 502181

Cluster: WP_233580373.1 hypothetical protein [Streptomyces triticirhizae]
Num Members: 1
Num Taxa: 1
Scientific Name: Streptomyces triticirhizae
Common Name : high G+C Gram-positive bacteria
Taxid: 2483353
Highest Bit Score: 108
Total Bit Score: 108
Percent Coverage: 87%
Evalue: 3e-24
Percent Identity: 40.37%
Accession Length: 591
1 cluster member(s):
Accession Scientific Common Taxid
WP_233580373.1 Streptomyces triticirhizae high G+C Gram-positive bact... 2483353
Cluster: WP_148057932.1 hypothetical protein [Pseudomonas frederiksbergensis]
Num Members: 1
Num Taxa: 1
Scientific Name: Pseudomonas frederiksbergensis
Common Name : g-proteobacteria
Taxid: 104087
Highest Bit Score: 101
Total Bit Score: 101
Percent Coverage: 66%

Evalue: 1e-23
Percent Identity: 41.46%
Accession Length: 174
1 cluster member(s):
Accession Scientific Common Taxid
WP_148057932.1 Pseudomonas frederiksbergensis g-proteobacteria 104087
Cluster: WP_102922765.1 polyketide synthase [Streptomyces noursei]
Num Members: 8
Num Taxa: 6
Scientific Name: Streptomyces
Common Name : high G+C Gram-positive bacteria
Taxid: 1883
Highest Bit Score: 100
Total Bit Score: 100
Percent Coverage: 75%
Evalue: 2e-23
Percent Identity: 43.48%
Accession Length: 172
8 cluster member(s):
Accession Scientific Common Taxid
WP_102922765.1 Streptomyces noursei high GC Gram+ 1971
AJC61551.1 Streptomyces sp. 769 high GC Gram+ 1262452
ANZ21401.1 Streptomyces noursei ATCC 1... high GC Gram+ 316284
PNE42909.1 Streptomyces noursei high GC Gram+ 1971

SHL34958.1	Streptomyces yunnanensis	high GC Gram+	156453
WP_039639483.1	Streptomyces sp. 769	high GC Gram+	1262452
WP_189866450.1	Streptomyces albulus	high GC Gram+	68570
WP_205360545.1	Streptomyces	high G+C Gram-positive bact...	1883
Cluster:	WP_223094296.1 hypothetical protein [Dactylosporangium vinaceum]		
Num Members:	1		
Num Taxa:	1		
Scientific Name:	Dactylosporangium vinaceum		
Common Name :	high G+C Gram-positive bacteria		
Taxid:	53362		
Highest Bit Score:	100		
Total Bit Score:	100		
Percent Coverage:	81%		
Evalue:	3e-23		
Percent Identity:	44.24%		
Accession Length:	168		
1 cluster member(s):			
Accession	Scientific	Common	Taxid
WP_223094296.1	Dactylosporangium vinaceum	high G+C Gram-positive bact...	53362
Cluster:	WP_032645024.1 hypothetical protein [Pseudomonas syringae group genomosp. 3]		
Num Members:	1		
Num Taxa:	1		
Scientific Name:	Pseudomonas syringae group genomosp. 3		

Common Name : g-proteobacteria
Taxid: 251701
Highest Bit Score: 96.7
Total Bit Score: 96.7
Percent Coverage: 60%
Evalue: 2e-22
Percent Identity: 52.25%
Accession Length: 119
1 cluster member(s):
Accession Scientific Common Taxid
WP_032645024.1 Pseudomonas syringae group ... g-proteobacteria 251701
Cluster: WP_042868955.1 hypothetical protein [Dickeya poaceiphila]
Num Members: 2
Num Taxa: 2
Scientific Name: Dickeya
Common Name : enterobacteria
Taxid: 204037
Highest Bit Score: 97.1
Total Bit Score: 97.1
Percent Coverage: 88%
Evalue: 7e-22
Percent Identity: 35.98%
Accession Length: 177
2 cluster member(s):

Accession	Scientific	Common	Taxid
WP_042868955.1	Dickeya poaceiphila	enterobacteria	568768
WP_168640281.1	Dickeya sp. CFBP 2040	enterobacteria	2718531

Cluster: WP_009114554.1 hypothetical protein [Brenneria]

Num Members: 2

Num Taxa: 2

Scientific Name: Brenneria

Common Name : enterobacteria

Taxid: 71655

Highest Bit Score: 96.7

Total Bit Score: 96.7

Percent Coverage: 89%

Evalue: 8e-22

Percent Identity: 34.34%

Accession Length: 177

2 cluster member(s):

Accession	Scientific	Common	Taxid
WP_009114554.1	Brenneria	enterobacteria	71655
WP_113868958.1	Brenneria salicis	enterobacteria	55214

Cluster: WP_190135286.1 polyketide synthase [Streptomyces longispororuber]

Num Members: 1

Num Taxa: 1

Scientific Name: Streptomyces longispororuber

Common Name : high G+C Gram-positive bacteria
Taxid: 68230
Highest Bit Score: 97.4
Total Bit Score: 97.4
Percent Coverage: 82%
Evalue: 1e-21
Percent Identity: 41.72%
Accession Length: 208
1 cluster member(s):
Accession Scientific Common Taxid
WP_190135286.1 Streptomyces longispororuber high G+C Gram-positive bact... 68230
Cluster: WP_084903002.1 hypothetical protein [Streptomyces sp. CB03238]
Num Members: 1
Num Taxa: 1
Scientific Name: Streptomyces sp. CB03238
Common Name : high GC Gram+
Taxid: 1907777
Highest Bit Score: 96.7
Total Bit Score: 96.7
Percent Coverage: 79%
Evalue: 2e-21
Percent Identity: 38.78%
Accession Length: 210
1 cluster member(s):

Accession	Scientific	Common	Taxid
WP_084903002.1	Streptomyces sp. CB03238	high GC Gram+	1907777
Cluster:	WP_142264107.1 hypothetical protein [Streptomyces sp. SLBN-115]		
Num Members:	11		
Num Taxa:	10		
Scientific Name:	Actinomycetia		
Common Name :	high G+C Gram-positive bacteria		
Taxid:	1760		
Highest Bit Score:	94.7		
Total Bit Score:	94.7		
Percent Coverage:	77%		
Evalue:	5e-21		
Percent Identity:	38.62%		
Accession Length:	182		
11 cluster member(s):			
Accession	Scientific	Common	Taxid
WP_142264107.1	Streptomyces sp. SLBN-115	high GC Gram+	2768453
AHJ60971.1	Streptomyces sp. B9173	high GC Gram+	1462558
KPI32276.1	Actinobacteria bacterium OV320	high GC Gram+	1592329
WP_052183718.1	Streptomyces sp. 303MFCol5.2	high GC Gram+	1172181
WP_057599905.1	Streptomyces sp. Root1310	high GC Gram+	1736452
WP_062649311.1	Streptomyces sp. NBRC 110468	high GC Gram+	1679753
WP_171397055.1	Streptomyces asoensis	high G+C Gram-positive bact...	249586
WP_189917436.1	Streptomyces asoensis	high G+C Gram-positive bact...	249586
WP_200727350.1	Streptomyces sp. MBT49	high GC Gram+	1488380

WP_200744728.1	Streptomyces sp. MBT97	high GC Gram+	2800411
WP_230195930.1	Streptomyces sp. CMB-FB	high GC Gram+	2864460
Cluster:	WP_131740835.1 hypothetical protein [Actinomadura roseirufa]		
Num Members:	1		
Num Taxa:	1		
Scientific Name:	Actinomadura roseirufa		
Common Name :	high G+C Gram-positive bacteria		
Taxid:	2094049		
Highest Bit Score:	92.8		
Total Bit Score:	92.8		
Percent Coverage:	76%		
Evalue:	3e-20		
Percent Identity:	39.72%		
Accession Length:	179		
1 cluster member(s):			
Accession	Scientific	Common	Taxid
WP_131740835.1	Actinomadura roseirufa	high G+C Gram-positive bact...	2094049
Cluster:	WP_233865706.1 hypothetical protein [Streptomyces sp. ST2-7A]		
Num Members:	1		
Num Taxa:	1		
Scientific Name:	Streptomyces sp. ST2-7A		
Common Name :	high GC Gram+		
Taxid:	2907214		

Highest Bit Score: 97.8
Total Bit Score: 97.8
Percent Coverage: 79%
Evalue: 3e-20
Percent Identity: 42.86%
Accession Length: 588
1 cluster member(s):
Accession Scientific Common Taxid
WP_233865706.1 Streptomyces sp. ST2-7A high GC Gram+ 2907214
Cluster: MBI3688123.1 polyketide synthase [Actinomycetia bacterium]
Num Members: 1
Num Taxa: 1
Scientific Name: Actinomycetia bacterium
Common Name : high GC Gram+
Taxid: 1883427
Highest Bit Score: 93.2
Total Bit Score: 93.2
Percent Coverage: 80%
Evalue: 4e-20
Percent Identity: 43.05%
Accession Length: 203
1 cluster member(s):
Accession Scientific Common Taxid
MBI3688123.1 Actinomycetia bacterium high GC Gram+ 1883427

Cluster: WP_086771546.1 hypothetical protein [Streptomyces bobili]
Num Members: 2
Num Taxa: 2
Scientific Name: Streptomyces
Common Name : high G+C Gram-positive bacteria
Taxid: 1883
Highest Bit Score: 92.0
Total Bit Score: 92.0
Percent Coverage: 85%
Evalue: 7e-20
Percent Identity: 34.59%
Accession Length: 182
2 cluster member(s):
Accession Scientific Common Taxid
WP_086771546.1 Streptomyces bobili high GC Gram+ 67280
WP_150470837.1 Streptomyces galilaeus high GC Gram+ 33899
Cluster: WP_219528372.1 hypothetical protein [Nonomuraea guangzhouensis]
Num Members: 1
Num Taxa: 1
Scientific Name: Nonomuraea guangzhouensis
Common Name : high G+C Gram-positive bacteria
Taxid: 1291555
Highest Bit Score: 92.0

Total Bit Score: 92.0
Percent Coverage: 82%
Evalue: 2e-19
Percent Identity: 40.40%
Accession Length: 235
1 cluster member(s):
Accession Scientific Common Taxid
WP_219528372.1 Nonomuraea guangzhouensis high G+C Gram-positive bact... 1291555
Cluster: WP_052408053.1 hypothetical protein [Allokutzneria albata]
Num Members: 1
Num Taxa: 1
Scientific Name: Allokutzneria albata
Common Name : high GC Gram+
Taxid: 211114
Highest Bit Score: 89.7
Total Bit Score: 89.7
Percent Coverage: 75%
Evalue: 2e-19
Percent Identity: 37.41%
Accession Length: 156
1 cluster member(s):
Accession Scientific Common Taxid
WP_052408053.1 Allokutzneria albata high GC Gram+ 211114

Cluster: WP_218064164.1 coronafacic acid synthetase component [Nguyenibacter vanlangensis]
Num Members: 1
Num Taxa: 1
Scientific Name: Nguyenibacter vanlangensis
Common Name : a-proteobacteria
Taxid: 1216886
Highest Bit Score: 89.4
Total Bit Score: 89.4
Percent Coverage: 76%
Evalue: 5e-19
Percent Identity: 32.62%
Accession Length: 173
1 cluster member(s):
Accession Scientific Common Taxid
WP_218064164.1 Nguyenibacter vanlangensis a-proteobacteria 1216886
Cluster: OLT10694.1 hypothetical protein BJF79_25760 [Actinomadura sp. CNU-125]
Num Members: 1
Num Taxa: 1
Scientific Name: Actinomadura sp. CNU-125
Common Name : high GC Gram+
Taxid: 1904961
Highest Bit Score: 92.4
Total Bit Score: 92.4
Percent Coverage: 91%

Evalue: 2e-18
Percent Identity: 43.53%
Accession Length: 632
1 cluster member(s):
Accession Scientific Common Taxid
OLT10694.1 Actinomadura sp. CNU-125 high GC Gram+ 1904961
Cluster: WP_201843267.1 hypothetical protein [Streptomyces actinomycinicus]
Num Members: 1
Num Taxa: 1
Scientific Name: Streptomyces actinomycinicus
Common Name : high GC Gram+
Taxid: 1695166
Highest Bit Score: 87.4
Total Bit Score: 87.4
Percent Coverage: 80%
Evalue: 4e-18
Percent Identity: 40.13%
Accession Length: 182
1 cluster member(s):
Accession Scientific Common Taxid
WP_201843267.1 Streptomyces actinomycinicus high GC Gram+ 1695166
Cluster: WP_052209145.1 hypothetical protein [Acinetobacter sp. A47]

Num Members: 1
Num Taxa: 1
Scientific Name: Acinetobacter sp. A47
Common Name : g-proteobacteria
Taxid: 1561217
Highest Bit Score: 85.9
Total Bit Score: 85.9
Percent Coverage: 67%
Evalue: 1e-17
Percent Identity: 34.40%
Accession Length: 170
1 cluster member(s):
Accession Scientific Common Taxid
WP_052209145.1 Acinetobacter sp. A47 g-proteobacteria 1561217
Cluster: NVN10070.1 coronafacic acid synthetase component [Nguyenibacter vanlangensis]
Num Members: 1
Num Taxa: 1
Scientific Name: Nguyenibacter vanlangensis
Common Name : a-proteobacteria
Taxid: 1216886
Highest Bit Score: 85.1
Total Bit Score: 85.1
Percent Coverage: 73%
Evalue: 2e-17
Percent Identity: 31.11%

Accession Length: 154
1 cluster member(s):
Accession Scientific Common Taxid
NVN10070.1 Nguyenibacter vanlangensis a-proteobacteria 1216886
Cluster: WP_189938222.1 coronafacic acid synthetase [Streptomyces aurantiogriseus]
Num Members: 1
Num Taxa: 1
Scientific Name: Streptomyces aurantiogriseus
Common Name : high GC Gram+
Taxid: 66870
Highest Bit Score: 85.1
Total Bit Score: 85.1
Percent Coverage: 77%
Evalue: 3e-17
Percent Identity: 37.93%
Accession Length: 182
1 cluster member(s):
Accession Scientific Common Taxid
WP_189938222.1 Streptomyces aurantiogriseus high GC Gram+ 66870
Cluster: MCC3772573.1 polyketide synthase [Streptomyces sp. UNOC14_S4]
Num Members: 1
Num Taxa: 1

Scientific Name: Streptomyces sp. UNOC14_S4
Common Name : high GC Gram+
Taxid: 2872340
Highest Bit Score: 80.5
Total Bit Score: 80.5
Percent Coverage: 59%
Evalue: 3e-16
Percent Identity: 44.55%
Accession Length: 109
1 cluster member(s):
Accession Scientific Common Taxid
MCC3772573.1 Streptomyces sp. UNOC14_S4 high GC Gram+ 2872340
Cluster: WP_162296926.1 hypothetical protein [Burkholderia ubonensis]
Num Members: 1
Num Taxa: 1
Scientific Name: Burkholderia ubonensis
Common Name : b-proteobacteria
Taxid: 101571
Highest Bit Score: 80.1
Total Bit Score: 80.1
Percent Coverage: 78%
Evalue: 2e-15
Percent Identity: 31.72%
Accession Length: 175

1 cluster member(s):
Accession Scientific Common Taxid
WP_162296926.1 Burkholderia ubonensis subs... b-proteobacteria 265293
Cluster: WP_190125360.1 polyketide synthase [Streptomyces inusitatus]
Num Members: 1
Num Taxa: 1
Scientific Name: Streptomyces inusitatus
Common Name : high G+C Gram-positive bacteria
Taxid: 68221
Highest Bit Score: 80.1
Total Bit Score: 80.1
Percent Coverage: 75%
Evalue: 3e-15
Percent Identity: 37.76%
Accession Length: 184
1 cluster member(s):
Accession Scientific Common Taxid
WP_190125360.1 Streptomyces inusitatus high G+C Gram-positive bact... 68221
Cluster: WP_217809648.1 hypothetical protein [Lonsdalea britannica]
Num Members: 1
Num Taxa: 1
Scientific Name: Lonsdalea britannica
Common Name : enterobacteria

Taxid: 1082704
Highest Bit Score: 73.9
Total Bit Score: 73.9
Percent Coverage: 54%
Evalue: 1e-13
Percent Identity: 40.59%
Accession Length: 118
1 cluster member(s):
Accession Scientific Common Taxid
WP_217809648.1 Lonsdalea britannica enterobacteria 1082704
Cluster: WP_116025564.1 polyketide synthase [Thermomonospora umbrina]
Num Members: 1
Num Taxa: 1
Scientific Name: Thermomonospora umbrina
Common Name : high G+C Gram-positive bacteria
Taxid: 111806
Highest Bit Score: 74.7
Total Bit Score: 74.7
Percent Coverage: 72%
Evalue: 1e-13
Percent Identity: 35.34%
Accession Length: 155
1 cluster member(s):
Accession Scientific Common Taxid

WP_116025564.1 Thermomonospora umbrina high G+C Gram-positive bact... 111806
Cluster: MBS0266747.1 hypothetical protein [Planctomycetes bacterium]
Num Members: 1
Num Taxa: 1
Scientific Name: Planctomycetes bacterium
Common Name : bacteria
Taxid: 2026780
Highest Bit Score: 75.1
Total Bit Score: 75.1
Percent Coverage: 86%
Evalue: 2e-13
Percent Identity: 31.88%
Accession Length: 196
1 cluster member(s):
Accession Scientific Common Taxid
MBS0266747.1 Planctomycetes bacterium bacteria 2026780
Cluster: WP_143238584.1 hypothetical protein [Agrobacterium tumefaciens]
Num Members: 2
Num Taxa: 1
Scientific Name: Agrobacterium tumefaciens
Common Name : a-proteobacteria
Taxid: 358
Highest Bit Score: 74.3

Total Bit Score: 74.3
Percent Coverage: 78%
Evalue: 3e-13
Percent Identity: 33.33%
Accession Length: 167
2 cluster member(s):
Accession   Scientific   Common   Taxid
WP_143238584.1   Agrobacterium tumefaciens   a-proteobacteria   358
OMP71389.1   Agrobacterium tumefaciens   a-proteobacteria   358
Cluster: WP_096724586.1 coronafacic acid synthetase [Paraburkholderia acidicola]
Num Members: 1
Num Taxa: 1
Scientific Name: Paraburkholderia acidicola
Common Name :   b-proteobacteria
Taxid: 1912599
Highest Bit Score: 73.6
Total Bit Score: 73.6
Percent Coverage: 74%
Evalue: 8e-13
Percent Identity: 31.39%
Accession Length: 179
1 cluster member(s):
Accession   Scientific   Common   Taxid
WP_096724586.1   Paraburkholderia acidicola   b-proteobacteria   1912599

Alignments:
>AmcG [Streptomyces novoguineensis]
Sequence ID: QCX41961.1 Length: 184
Range 1: 1 to 184
Score:367 bits(941), Expect:3e-128,
Method:Compositional matrix adjust.,
Identities:184/184(100%), Positives:184/184(100%), Gaps:0/184(0%)
Query 1 MNVFPASEPYVIFPPDTVDVAVVGHGDAFREELTAVRSRVPSLYADPLAWLVADAAESAL 60
MNVFPASEPYVIFPPDTVDVAVVGHGDAFREELTAVRSRVPSLYADPLAWLVADAAESAL
Sbjct 1 MNVFPASEPYVIFPPDTVDVAVVGHGDAFREELTAVRSRVPSLYADPLAWLVADAAESAL 60
Query 61 TQCATEVLAAREDVAVILVSAHCTLHTMDAMARSLPAPRVSPLRFAGANPGSAGSLLCLL 120
TQCATEVLAAREDVAVILVSAHCTLHTMDAMARSLPAPRVSPLRFAGANPGSAGSLLCLL
Sbjct 61 TQCATEVLAAREDVAVILVSAHCTLHTMDAMARSLPAPRVSPLRFAGANPGSAGSLLCLL 120
Query 121 HGFKGSSLTLSMTPEDGLPAARTVARSWLRSGAASFVVLCTHRGHGEKGHSVRCAVLAPA 180
HGFKGSSLTLSMTPEDGLPAARTVARSWLRSGAASFVVLCTHRGHGEKGHSVRCAVLAPA
Sbjct 121 HGFKGSSLTLSMTPEDGLPAARTVARSWLRSGAASFVVLCTHRGHGEKGHSVRCAVLAPA 180
Query 181 AGRR 184
AGRR
Sbjct 181 AGRR 184

>coronafacic acid synthetase [Streptomyces pilosus]
Sequence ID: WP_189561794.1 Length: 197
Range 1: 1 to 182
Score:327 bits(837), Expect:4e-112,
Method:Compositional matrix adjust.,
Identities:162/182(89%), Positives:170/182(93%), Gaps:0/182(0%)
Query 1 MNVFPASEPYVIFPPDTVDVAVVGHGDAFREELTAVRSRVPSLYADPLAWLVADAAESAL 60
MN FPASEPYVI PPDT DVAVVGHGDA+REELTAVRSRVPSLYADPLAWLVADA E AL
Sbjct 1 MNTFPASEPYVICPPDTADVAVVGHGDAYREELTAVRSRVPSLYADPLAWLVADAVEPAL 60
Query 61 TQCATEVLAAREDVAVILVSAHCTLHTMDAMARSLPAPRVSPLRFAGANPGSAGSLLCLL 120
+CA +V AAREDVAVILVSAHCTLHTMDA+ARSLPAPRVSPLRFAGANPGSAGSLLCLL
Sbjct 61 AECAADVRAAREDVAVILVSAHCTLHTMDAVARSLPAPRVSPLRFAGANPGSAGSLLCLL 120
Query 121 HGFKGSSLTLSMTPEDGLPAARTVARSWLRSGAASFVVLCTHRGHGEKGHSVRCAVLAPA 180
HGF+GSSLT SMTPEDGLPAARTVARSWLRSG+AS+VVLC H+ HGEKGHSVRCAVLAPA
Sbjct 121 HGFRGSSLTFSMTPEDGLPAARTVARSWLRSGSASYVVLCAHQRHGEKGHSVRCAVLAPA 180
Query 181 AG 182
G
Sbjct 181 VG 182

>coronafacic acid synthetase [Streptomyces sp. GMR22]
Sequence ID: WP_182468098.1 Length: 197
Range 1: 1 to 182
Score:308 bits(790), Expect:5e-105,
Method:Compositional matrix adjust.,
Identities:154/182(85%), Positives:165/182(90%), Gaps:0/182(0%)
Query 1 MNVFPASEPYVIFPPDTVDVAVVGHGDAFREELTAVRSRVPSLYADPLAWLVADAAESAL 60
MN FPA PY I PPDT DVAVVGHGDA RE+LTAVRSR+PSLYADPLAWLVADA E+AL
Sbjct 1 MNAFPAFGPYAICPPDTADVAVVGHGDASREQLTAVRSRIPSLYADPLAWLVADAVENAL 60
Query 61 TQCATEVLAAREDVAVILVSAHCTLHTMDAMARSLPAPRVSPLRFAGANPGSAGSLLCLL 120
++CA +VLA REDVAVILVSAHCTLHTMDA+ARSLPAPRVSPLRFAGA+PGSAGSLLCLL
Sbjct 61 SECAADVLACREDVAVILVSAHCTLHTMDAVARSLPAPRVSPLRFAGASPGSAGSLLCLL 120
Query 121 HGFKGSSLTLSMTPEDGLPAARTVARSWLRSGAASFVVLCTHRGHGEKGHSVRCAVLAPA 180
HGFKGSSLT SMTPEDGLPAARTVARSWLRSGAA +VVLC H+ H +GHSVRCAVLAPA
Sbjct 121 HGFKGSSLTFSMTPEDGLPAARTVARSWLRSGAAPYVVLCAHQRHEGQGHSVRCAVLAPA 180
Query 181 AG 182
G
Sbjct 181 VG 182

>hypothetical protein [Streptomyces haliclonae]
Sequence ID: MBB4673076.1 Length: 202
Range 1: 1 to 180
Score:292 bits(747), Expect:3e-98,
Method:Compositional matrix adjust.,
Identities:148/180(82%), Positives:158/180(87%), Gaps:0/180(0%)
Query 1   MNVFPASEPYVIFPPDTVDVAVVGHGDAFREELTAVRSRVPSLYADPLAWLVADAAESAL 60
MN PAS PY + PPDT DVAVV G+AFRE+LTAVRSR+PSLYADPLAWLVADA +SAL
Sbjct 1   MNAIPASGPYAVCPPDTEDVAVVAQGNAFREQLTAVRSRIPSLYADPLAWLVADAVDSAL 60
Query 61   TQCATEVLAAREDVAVILVSAHCTLHTMDAMARSLPAPRVSPLRFAGANPGSAGSLLCLL 120
T+CA VLA REDVAVILVSAHCTLHTMDAMAR+LPAPRVSPLRFAGA+PGSAGSLLC L
Sbjct 61   TECADGVLAHREDVAVILVSAHCTLHTMDAMARTLPAPRVSPLRFAGASPGSAGSLLCQL 120
Query 121 HGFKGSSLTLSMTPEDGLPAARTVARSWLRSGAASFVVLCTHRGHGEKGHSVRCAVLAPA 180
HGFKG SLTLSMTP DGLPAARTVARSWL SGAAS VVLC H+   +GHSVRCAVLAPA
Sbjct 121 HGFKGPSLTLSMTPGDGLPAARTVARSWLLSGAASHVVLCAHQRQEGEGHSVRCAVLAPA 180
>coronafacic acid synthetase [Nonomuraea lactucae]
Sequence ID: WP_162795002.1 Length: 173
Range 1: 6 to 164

Score:192 bits(489), Expect:2e-59,
Method:Compositional matrix adjust.,
Identities:97/159(61%), Positives:118/159(74%), Gaps:0/159(0%)
Query 20 VAVVGHGDAFREELTAVRSRVPSLYADPLAWLVADAAESALTQCATEVLAAREDVAVILV 79
+A V G+A+R ELT VR +P+LYADP+AWLVADA ALT C +V AAR+DV ILV
Sbjct 6 LATVARGEAYRPELTTVRGSIPNLYADPVAWLVADAVRDALTACPGDVAAARDDVGTILV 65
Query 80 SAHCTLHTMDAMARSLPAPRVSPLRFAGANPGSAGSLLCLLHGFKGSSLTLSMTPEDGLP 139
SAHCT HTM A+A S RVSPLRFAGA+PG+AGSL C++HGF+G +LTLS DGLP
Sbjct 66 SAHCTRHTMRAVAGSAAGGRVSPLRFAGASPGTAGSLACIVHGFRGPALTLSTGARDGLP 125
Query 140 AARTVARSWLRSGAASFVVLCTHRGHGEKGHSVRCAVLA 178
AR +ARSWLRSGAA++V++ H + GH VR VLA
Sbjct 126 TARVLARSWLRSGAAAYVIVSVHGVDAQGGHRVRSVVLA 164
>coronafacic acid synthetase [Spongiactinospora gelatinilytica]
Sequence ID: WP_111167667.1 Length: 179
Range 1: 16 to 173
Score:176 bits(445), Expect:1e-52,
Method:Compositional matrix adjust.,
Identities:97/158(61%), Positives:117/158(74%), Gaps:0/158(0%)

Query 20 VAVVGHGDAFREELTAVRSRVPSLYADPLAWLVADAAESALTQCATEVLAAREDVAVILV 79
V V+ G+A R EL VRSR+PSLYADPLAWLV DA E AL+ C+ E+ AR++VAVIL+
Sbjct 16 VVVLATGEATRAELRPVRSRIPSLYADPLAWLVLDAVEDALSTCSDELYEARDEVAVILI 75
Query 80 SAHCTLHTMDAMARSLPAPRVSPLRFAGANPGSAGSLLCLLHGFKGSSLTLSMTPEDGLP 139
S CTL TM+A+ARS P RVSPLRFAGA+PG GSL C+ GF+G SLTL+ P +GLP
Sbjct 76 SPFCTLQTMEAVARSAPGGRVSPLRFAGASPGGGGSLTCVARGFRGPSLTLATRPGNGLP 135
Query 140 AARTVARSWLRSGAASFVVLCTHRGHGEKGHSVRCAVL 177
ART+ARSWLRSG A++ V+ H E GH VR AVL
Sbjct 136 VARTLARSWLRSGQAAYAVVSAHEAMAEGGHRVRTAVL 173
>coronafacic acid synthetase [Spongiactinospora rosea]
Sequence ID: WP_113985346.1 Length: 175
Range 1: 12 to 175
Score:175 bits(443), Expect:1e-52,
Method:Compositional matrix adjust.,
Identities:99/164(60%), Positives:118/164(71%), Gaps:0/164(0%)
Query 20 VAVVGHGDAFREELTAVRSRVPSLYADPLAWLVADAAESALTQCATEVLAAREDVAVILV 79
V V G+A EL VRSR+PSLYADPLAWLV DA E AL+ C+ E+ AR+DVAVIL+
Sbjct 12 VVVRATGEAACAELRPVRSRIPSLYADPLAWLVVDAVEDALSTCSDELYEARDDVAVILI 71
Query 80 SAHCTLHTMDAMARSLPAPRVSPLRFAGANPGSAGSLLCLLHGFKGSSLTLSMTPEDGLP 139

S CTL TM+A+AR+ P RVSPLRFAGA+PG GSL C+ GF+G SLTL+ P+ GLP
Sbjct 72   SPLCTLLTMEAVARAAPGGRVSPLRFAGASPGGGGSLTCVARGFRGPSLTLATHPDRGLP 131
Query 140 AARTVARSWLRSGAASFVVLCTHRGHGEKGHSVRCAVLAPAAGR 183
ART+ RSWLRSG A++ V+ H   E GH VRC VLAP GR
Sbjct 132 VARTLVRSWLRSGQAAYAVVSAHEATAEGGHRVRCGVLAPEPGR 175
>hypothetical protein [Kibdelosporangium banguiense]
Sequence ID: WP_209647138.1 Length: 173
Range 1: 13 to 169
Score:160 bits(404), Expect:1e-46,
Method:Compositional matrix adjust.,
Identities:82/159(52%), Positives:111/159(69%), Gaps:2/159(1%)
Query 19   DVAVVGHGDAFREELTAVRSRVPSLYADPLAWLVADAAESALTQCATEVLAAREDVAVIL 78
D+ V+GHG   R E   R R+PS+Y DP++WLV +A + AL C   V + ++VAV+L
Sbjct 13   DLVVLGHGGTDRAEPDG-RRRIPSMYVDPVSWLVLEAVDEALADCPDTVRQSPDEVAVML 71
Query 79   VSAHCTLHTMDAMARSLPAPRVSPLRFAGANPGSAGSLLCLLHGFKGSSLTLSMTPEDGL 138
VS H TLHTM +A+++P+ R+SPLRFAGA+PG A SL CL+H F+G SL ++ TP++G
Sbjct 72   VSTHATLHTMTEIAKAIPSGRLSPLRFAGASPGGAASLTCLVHKFRGPSLMMTTTPDEGW 131
Query 139 PAARTVARSWLRSGAASFVVLCTHRGHGEKGHSVRCAVL 177
PAA T+AR WLR+GAA+ VVL H   + GH VR VL

Sbjct 132 PAALTMARFWLRTGAATSVVLSAHTA-SDHGHRVRTVVL 169
>hypothetical protein [Kibdelosporangium sp. MJ126-NF4]
Sequence ID: WP_052479233.1 Length: 171
Range 1: 6 to 165
Score:153 bits(387), Expect:5e-44,
Method:Compositional matrix adjust.,
Identities:83/163(51%), Positives:106/163(65%), Gaps:3/163(1%)
Query 15 PDTVDVAVVGHGDAFREELTAVRSRVPSLYADPLAWLVADAAESALTQCATEVLAAREDV 74
PD D+ ++G G R E A R R+PS+YADP+AWLV +A + AL C V A +DV
Sbjct 6 PD--DLTILGEGRVERAE-PARRQRIPSMYADPVAWLVLEAIDQALADCMDTVRQAADDV 62
Query 75 AVILVSAHCTLHTMDAMARSLPAPRVSPLRFAGANPGSAGSLLCLLHGFKGSSLTLSMTP 134
AVILVS H T+ TM +AR+ R+SPLRFAGA+PG A SL C++H +G SL L+ P
Sbjct 63 AVILVSTHATVDTMADVARATETGRLSPLRFAGASPGGAASLACIVHSLRGPSLLLTTEP 122
Query 135 EDGLPAARTVARSWLRSGAASFVVLCTHRGHGEKGHSVRCAVL 177
G P A TVAR WLR+ AAS V+L H ++GH VR A+L
Sbjct 123 GTGWPTALTVARCWLRTAAASQVLLSAHTADAQQGHQVRTALL 165

>coronafacic acid synthetase [Streptomyces geranii]
Sequence ID: WP_105967719.1 Length: 179
Range 1: 7 to 179
Score:143 bits(360), Expect:6e-40,
Method:Compositional matrix adjust.,
Identities:84/174(48%), Positives:105/174(60%), Gaps:5/174(2%)
Query 15 PDTVDVAVVGHGDAFREELTAVRSRVPSLYADPLAWLVADAAESAL--TQCATEVLAARE 72
P T + VG G A +L+A R SLYADPL+WLV DA E AL + E +A+E
Sbjct 7 PHTYGMKAVGEGVAVAPDLSAAPRRRASLYADPLSWLVLDAVEQALGASDFTPEDASAKE 66
Query 73 DVAVILVSAHCTLHTMDAMARSLPAPRVSPLRFAGANPGSAGSLLCLLHGFKGSSLTLSM 132
V I +S CTLHTM +A ++PA R+SPLRF+GANPGS +L GF G ++TLSM
Sbjct 67 AVGHIAISDQCTLHTMRQLAEAIPAGRISPLRFSGANPGSICTLPSQFLGFSGPTMTLSM 126
Query 133 TPEDGLPAARTVARSWLRSGAASFVVLCTHRGHGEKGHSVRCAVLA--PAAGRR 184
PE GLP A VAR+WLR G A+ V++ HR GH V VL P AG +
Sbjct 127 PPEKGLPPALAVARAWLRQGCATHVIVTAHRADA-SGHRVTSTVLTADPTAGPK 179
>hypothetical protein [Kribbella italica]
Sequence ID: WP_184795263.1 Length: 166
Range 1: 8 to 162
Score:140 bits(354), Expect:3e-39,

Method:Compositional matrix adjust.,
Identities:78/159(49%), Positives:103/159(64%), Gaps:4/159(2%)
Query 19 DVAVVGHGDAFREELTAVRSRVPSLYADPLAWLVADAAESALTQCATEVLAAREDVAVIL 78
D+ V G G A + + R R+PSLY DP+AWL+ ++AE A+ +V R +VAV++
Sbjct 8 DLTVFGSGTAAQAVPNSTR-RIPSLYVDPVAWLLLESAERAIGNNLDDV---RGEVAVLV 63
Query 79 VSAHCTLHTMDAMARSLPAPRVSPLRFAGANPGSAGSLLCLLHGFKGSSLTLSMTPEDGL 138
VS + TL TM +A + + RVSPLRFAGA+PG A SL CL+HGF+G SL L+ PE
Sbjct 64 VSTYATLDTMAGIAGTATSGRVSPLRFAGASPGGAASLTCLVHGFRGPSLLLTSGPESSW 123
Query 139 PAARTVARSWLRSGAASFVVLCTHRGHGEKGHSVRCAVL 177
P A T+ARSWLR+GAA V+L H E GH VR V+
Sbjct 124 PVAVTMARSWLRTGAAVRVILSVHTVDAEAGHQVRSVVV 162
>coronafacic acid synthetase [Streptomycetaceae bacterium]
Sequence ID: MBV9022849.1 Length: 176
Range 1: 9 to 169
Score:140 bits(354), Expect:5e-39,
Method:Compositional matrix adjust.,
Identities:79/162(49%), Positives:102/162(62%), Gaps:2/162(1%)
Query 17 TVDVAVVGHGDAFREELTAVR-SRVPSLYADPLAWLVADAAESALTQCATEVLAAREDVA 75
T +A+VG G A +L+ +R R SLYADPL+WLV DA E A+ C E L+ RE V

Sbjct 9 TCGMAIVGQGVATAPDLSGLRPKRKASLYADPLSWLVLDAVEHAVDDCGEEFLSERESVG 68
Query 76 VILVSAHCTLHTMDAMARSLPAPRVSPLRFAGANPGSAGSLLCLLHGFKGSSLTLSMTPE 135
I+VS CT+HTM +A ++PA R+SPLRF+GANPGS SL L GF G S+TLSM E
Sbjct 69 HIVVSDQCTIHTMREIAAAIPAGRISPLRFSGANPGSVCSLPSQLLGFSGPSMTLSMPAE 128
Query 136 DGLPAARTVARSWLRSGAASFVVLCTHRGHGEKGHSVRCAVL 177
GLP A+ +A WL +A+ V+L +H G GH V +
Sbjct 129 KGLPFAKVLAGIWLGQHSAAHVLLTSHTA-GAAGHRVTSTIF 169
>coronafacic acid synthetase [Streptomyces sp. GMR22]
Sequence ID: WP_182471800.1 Length: 188
Range 1: 37 to 173
Score:140 bits(354), Expect:7e-39,
Method:Compositional matrix adjust.,
Identities:71/137(52%), Positives:91/137(66%), Gaps:0/137(0%)
Query 41 PSLYADPLAWLVADAAESALTQCATEVLAAREDVAVILVSAHCTLHTMDAMARSLPAPRV 100
PS YADP++WLVA + +AL C +++ ++VAVI VS HCTL TM +A +P RV
Sbjct 37 PSFYADPVSWLVAQSVRAALDACPEDLVGRADEVAVITVSDHCTLRTMREIAGQVPTGRV 96
Query 101 SPLRFAGANPGSAGSLLCLLHGFKGSSLTLSMTPEDGLPAARTVARSWLRSGAASFVVLC 160
SPLRF+GANPGS GSL +L GF+G SLTLSM P+ GLP AR +A WL AA++ V+
Sbjct 97 SPLRFSGANPGSVGSLPGILLGFQGPSLTLSMPPDTGLPVARAIAHGWLAERAAYAVVS 156

Query 161 THRGHGEKGHSVRCAVL 177
H E H VR +L
Sbjct 157 AHEVSREGRHEVRTTIL 173
>hypothetical protein [Nocardia sp. BMG51109]
Sequence ID: WP_024802760.1 Length: 183
Range 1: 16 to 176
Score:140 bits(352), Expect:1e-38,
Method:Compositional matrix adjust.,
Identities:77/161(48%), Positives:98/161(60%), Gaps:1/161(0%)
Query 20 VAVVGHGDAFREELTAV-RSRVPSLYADPLAWLVADAAESALTQCATEVLAAREDVAVIL 78
+AV G G A L AV R RVPS+YADPLAWLV DA E A+ CA + A ++ V I
Sbjct 16 LAVAGRGVAVAPGLDAVPRRRVPSVYADPLAWLVLDAVEQAIDDCADNLAAVKDSVGHIA 75
Query 79 VSAHCTLHTMDAMARSLPAPRVSPLRFAGANPGSAGSLLCLLHGFKGSSLTLSMTPEDGL 138
VS +CT HTM +A ++ +SPLRF+GANPG+ L C L G S TLSM P GL
Sbjct 76 VSDYCTTHTMLGIAATVADGHISPLRFSGANPGAVAGLSCQLLALCGPSTTLSMPPYAGL 135
Query 139 PAARTVARSWLRSGAASFVVLCTHRGHGEKGHSVRCAVLAP 179
P A +AR WLR GAA ++++ TH + H++ VL P
Sbjct 136 PTAVALARMWLRQGAAEYMIISTHHTGDQGHHTISSTVLGP 176

>hypothetical protein [Streptomyces griseoruber]
Sequence ID: WP_055634613.1 Length: 182
Range 1: 7 to 173
Score:137 bits(345), Expect:1e-37,
Method:Compositional matrix adjust.,
Identities:80/168(48%), Positives:101/168(60%), Gaps:6/168(3%)
Query 15 PDTVDVAVVGHGDAFREELTAVR-SRVPSLYADPLAWLVADAAESALTQCATEVLAA--- 70
P T + VVG G A +L+A R SLYADPL+WLV DA E AL ++ +A
Sbjct 7 PHTYGMKVVGEGVATAPDLSAAPPKRKASLYADPLSWLVLDAVEQALGAHGGDLTSADAS 66
Query 71 -REDVAVILVSAHCTLHTMDAMARSLPAPRVSPLRFAGANPGSAGSLLCLLHGFKGSSLT 129
+E V I VS CT+HTM +A ++P R+SPLRF+GANP S SL L GF G ++T
Sbjct 67 VKEAVGHIAVSDQCTMHTMRQLAEAIPTARISPLRFSGANPASICSLPSQLLGFSGPTMT 126
Query 130 LSMTPEDGLPAARTVARSWLRSGAASFVVLCTHRGHGEKGHSVRCAVL 177
LSM PE GLP A VAR+WLR G A+ V++ HR GH V +L
Sbjct 127 LSMAPEKGLPPAMAVARAWLRQGTATHVIVTAHRADAS-GHRVTSTIL 173
>hypothetical protein [Pseudonocardia xinjiangensis]
Sequence ID: WP_169393912.1 Length: 174

Range 1: 31 to 174
Score:136 bits(342), Expect:3e-37,
Method:Compositional matrix adjust.,
Identities:70/144(49%), Positives:93/144(64%), Gaps:0/144(0%)
Query 37 RSRVPSLYADPLAWLVADAAESALTQCATEVLAAREDVAVILVSAHCTLHTMDAMARSLP 96
R R+PSLYADP +W V + + C V AA E A++LVS + T TM A++R++P
Sbjct 31 RRRIPSLYADPASWAVLETVDEVWADCPDAVRAAPEATAMVLVSTYGTTDTMTAISRAVP 90
Query 97 APRVSPLRFAGANPGSAGSLLCLLHGFKGSSLTLSMTPEDGLPAARTVARSWLRSGAASF 156
R+SPLRFAGA+ G SL+CL+HGF+G +L ++ P DG P A T+AR WLR+GA S
Sbjct 91 GGRLSPLRFAGASAGGPISLVCLVHGFRGPTLMITTDPVDGCPVALTMARHWLRTGAGSH 150
Query 157 VVLCTHRGHGEKGHSVRCAVLAPA 180
VV+ H +GH VRC VL A
Sbjct 151 VVVGVHDHDPLRGHQVRCVVLGAA 174
>MULTISPECIES: coronafacic acid synthetase [Streptomyces]
Sequence ID: WP_086756671.1 Length: 182
Range 1: 1 to 180
Score:135 bits(341), Expect:5e-37,
Method:Compositional matrix adjust.,
Identities:89/190(47%), Positives:109/190(57%), Gaps:18/190(9%)

Query 1 MNVFPASEPYVIFPPDTVDVAVVGHGDAFREELTAVR-SRVPSLYADPLAWLVADAAESA 59
MN PA   P T + VG G A   +L+A   R SLYADPL+WLV DA E A
Sbjct 1 MNRLPA--------PHTHALKAVGEGVAVARDLSATPPRRKASLYADPLSWLVLDAVEQA 52
Query 60 LTQCA-----TEVLAAREDVAVILVSAHCTLHTMDAMARSLPAPRVSPLRFAGANPGSAG 114
L C   T+V +AR V I VS CT HTM +A ++P+ R+SPLRF+GANPGS
Sbjct 53 LGACGGVLTPTDV-SARRSVGHIAVSDQCTTHTMRQLAEAIPSGRISPLRFSGANPGSIC 111
Query 115 SLLCLLHGFKGSSLTLSMTPEDGLPAARTVARSWLRSGAASFVVLCTHRGHGEKGHSVRC 174
SL   L G G ++TLSM P+ GLP A VAR+WLR +AS V++ THR   GH V
Sbjct 112 SLPSQLLGLSGPTMTLSMPPDKGLPPALLVARAWLRQDSASHVIVTTHRADAS-GHHVTS 170
Query 175 AVLA--PAAG 182
+L   P AG
Sbjct 171 TILTSDPTAG 180
>hypothetical protein [Streptomyces scabiei]
Sequence ID: WP_059080167.1 Length: 175
Range 1: 9 to 169
Score:133 bits(335), Expect:4e-36,
Method:Compositional matrix adjust.,
Identities:76/162(47%), Positives:103/162(63%), Gaps:2/162(1%)

Query 17 TVDVAVVGHGDAFREELTAVRSRV-PSLYADPLAWLVADAAESALTQCATEVLAAREDVA 75
T + V+G G +L+ VR R SLYADPL+WLV +A + A+ ++ +ARE V
Sbjct 9 TYGMTVLGEGVTEVTDLSGVRPRQRASLYADPLSWLVFEAVQHAIDAHREDIDSAREGVG 68
Query 76 VILVSAHCTLHTMDAMARSLPAPRVSPLRFAGANPGSAGSLLCLLHGFKGSSLTLSMTPE 135
I+VS CT +TM A+A ++ R+SPLRF+GANPGS SL GF G S+TLSM P+
Sbjct 69 HIVVSDDCTTYTMRAIAATIAVGRISPLRFSGANPGSVCSLPSQFLGFSGPSMTLSMPPD 128
Query 136 DGLPAARTVARSWLRSGAASFVVLCTHRGHGEKGHSVRCAVL 177
GLP A VAR+WLR G+A+ V++ +HR G GH V +L
Sbjct 129 KGLPPAAAVARAWLRQGSATHVLVTSHRA-GPSGHRVTSTLL 169
>coronafacic acid synthetase [Bacillus cereus]
Sequence ID: WP_098276132.1 Length: 180
Range 1: 26 to 169
Score:133 bits(335), Expect:5e-36,
Method:Compositional matrix adjust.,
Identities:60/144(42%), Positives:85/144(59%), Gaps:0/144(0%)
Query 27 DAFREELTAVRSRVPSLYADPLAWLVADAAESALTQCATEVLAAREDVAVILVSAHCTLH 86
++ +L ++ VPS+YADP +WLV DA E A+ C ++ R +V VI +SA CT H
Sbjct 26 ESISTDLNTIKKSVPSIYADPASWLVLDALEKAIKTCNIDIEKDRIEVGVIAISAQCTKH 85
Query 87 TMDAMARSLPAPRVSPLRFAGANPGSAGSLLCLLHGFKGSSLTLSMTPEDGLPAARTVAR 146

TM +A + R+SP+RFAGANPGS L C+++GFKG SLT SM P D L T++
Sbjct 86 TMHRIANQVKGGRISPIRFAGANPGSLAGLPCIIYGFKGPSLTFSMLPNDCLETVNTLSI 145
Query 147 SWLRSGAASFVVLCTHRGHGEKGH 170
SW R F+++ + H
Sbjct 146 SWFRQKIIRFLIVNCYEIDDCNNH 169
>coronafacic acid synthetase [Bacillus cereus group sp. BfR-BA-01358]
Sequence ID: WP_242226323.1 Length: 149
Range 1: 1 to 127
Score:130 bits(326), Expect:4e-35,
Method:Compositional matrix adjust.,
Identities:57/127(45%), Positives:79/127(62%), Gaps:0/127(0%)
Query 33 LTAVRSRVPSLYADPLAWLVADAAESALTQCATEVLAAREDVAVILVSAHCTLHTMDAMA 92
+ ++ VPS+YADP +WLV DA E A+ C ++ R +V VI +SA CT HTM +
Sbjct 1 MNTIKKSVPSIYADPASWLVLDALEKAIKTCNIDIEKDRIEVGVIAISAQCTKHTMHRIT 60
Query 93 RSLPAPRVSPLRFAGANPGSAGSLLCLLHGFKGSSLTLSMTPEDGLPAARTVARSWLRSG 152
+ R+SP+RFAGANPGS L C+++GFKG SLT SM P D L T++ SW R
Sbjct 61 NQVKGGRISPIRFAGANPGSLAGLPCIIYGFKGPSLTFSMLPNDCLETVNTLSISWFRQK 120
Query 153 AASFVVL 159
F+++

Sbjct 121 IIRFLIV 127
>hypothetical protein [Actinosynnema sp. ALI-1.44]
Sequence ID: WP_076986735.1 Length: 156
Range 1: 4 to 153
Score:128 bits(321), Expect:3e-34,
Method:Compositional matrix adjust.,
Identities:75/157(48%), Positives:98/157(62%), Gaps:7/157(4%)
Query 19 DVAVVGHGDAFREELTAVRSRVPSLYADPLAWLVADAAESALTQCATEVLAAREDVAVIL 78
++AV+G G A + R+PS+Y DP+AWLV A ++AL +C V + +DVAVIL
Sbjct 4 ELAVLGEGHA---DGVGTERRIPSMYVDPVAWLVLAAIDAALAECPDSVQKSPDDVAVIL 60
Query 79 VSAHCTLHTMDAMARSLPAPRVSPLRFAGANPGSAGSLLCLLHGFKGSSLTLSMTPEDGL 138
+S H T TM +A R+SPLRFAGA+PG A SL C +HGF+G SL L+ P+ G
Sbjct 61 ISTHATRDTMAEIA----GGRLSPLRFAGASPGGAASLACRVHGFRGPSLLLTTGPQVGR 116
Query 139 PAARTVARSWLRSGAASFVVLCTHRGHGEKGHSVRCA 175
A TVA+SWLR+GAA+ VVL H HSV A
Sbjct 117 QTALTVAQSWLRTGAAAHVVLSAHTTDPHHVHSVVLA 153

>hypothetical protein [Kibdelosporangium philippinense]
Sequence ID: MCE7004706.1 Length: 190
Range 1: 40 to 180
Score:127 bits(320), Expect:8e-34,
Method:Compositional matrix adjust.,
Identities:68/142(48%), Positives:93/142(65%), Gaps:1/142(0%)
Query 39 RVPSLYADPLAWLVADAAESALTQCATEVLAAREDVAVILVSAHCTLHTMDAMARSLPAP 98
R+PSLYADP +W V +A + C EV A A++LVS + TL TM A+AR++P
Sbjct 40 RIPSLYADPASWAVLEAVDELWAGCPDEVRAP-GTTAMVLVSTYSTLDTMSAIARAVPGG 98
Query 99 RVSPLRFAGANPGSAGSLLCLLHGFKGSSLTLSMTPEDGLPAARTVARSWLRSGAASFVV 158
R+SPLRFAGA+ G SL+CL+HG +G +LT++ P GLPAA T+ R WLR+GAAS V
Sbjct 99 RLSPLRFAGASAGGPISLVCLVHGLRGPTLTITTDPAGGLPAALTMTRHWLRTGAASHGV 158
Query 159 LCTHRGHGEKGHSVRCAVLAPA 180
+ H +GH V+C ++ A
Sbjct 159 VAVHHHDPLRGHEVQCVLVGAA 180
>MULTISPECIES: coronafacic acid synthetase [unclassified Nonomuraea]
Sequence ID: WP_138200933.1 Length: 190
Range 1: 41 to 180
Score:127 bits(318), Expect:2e-33,

Method:Compositional matrix adjust.,
Identities:67/141(48%), Positives:92/141(65%), Gaps:1/141(0%)
Query 40 VPSLYADPLAWLVADAAESALTQCATEVLAAREDVAVILVSAHCTLHTMDAMARSLPAPR 99
+PSLYADP +W V +A + E + A E A++LVS H T+ TM +AR+ R
Sbjct 41 IPSLYADPASWAVLEAVDELWAGFPDE-MQAPETTAMVLVSTHGTVDTMAEIARAAARGR 99
Query 100 VSPLRFAGANPGSAGSLLCLLHGFKGSSLTLSMTPEDGLPAARTVARSWLRSGAASFVVL 159
+SP+RFAGA+ G SL+C++HG +G +L ++ P +GLPAART+AR WLR+GAAS VV+
Sbjct 100 LSPMRFAGASAGGPISLVCMVHGLRGPTLVITTDPANGLPAARTMARHWLRTGAASHVVV 159
Query 160 CTHRGHGEKGHSVRCAVLAPA 180
H +GH VRC V PA
Sbjct 160 AAHDRDPLRGHEVRCVVAGPA 180
>MULTISPECIES: hypothetical protein [Streptomyces]
Sequence ID: WP_053761438.1 Length: 191
Range 1: 16 to 181
Score:127 bits(318), Expect:2e-33,
Method:Compositional matrix adjust.,
Identities:74/167(44%), Positives:96/167(57%), Gaps:2/167(1%)
Query 15 PDTVDVAVVGHGDAFREELTAVRSRVPSLYADPLAWLVADAAESALTQCATEVLAAREDV 74
P+ +AV+G R R+PS YADP +W V +A ++ C EV AA E

Sbjct 16   PENSGLAVLGTARVSRSADAPPTRRIPSFYADPASWAVLEAVDALRAGCP-EVTAAPETT 74
Query 75   AVILVSAHCTLHTMDAMARSLPAPRVSPLRFAGANPGSAGSLLCLLHGFKGSSLTLSMTP 134
A ++V+ H TL TM +A LP RVSPLRFAGAN G   SL+C+ HG +G +LTL+
Sbjct 75   ATVMVTTHSTLDTMRVIAGGLPGGRVSPLRFAGANAGGPVSLVCMTHGLRGPTLTLTSDG 134
Query 135 EDGLPAARTVARSWLRSGAASFVVLCTHRGHGEKG-HSVRCAVLAPA 180
+G P A T+AR WLR+GAA VV+ H   G H VRCA + A
Sbjct 135 TEGAPTALTMARHWLRTGAADRVVIVAHHHDAALGRHEVRCAAVGAA 181
>hypothetical protein [Nonomuraea candida]
Sequence ID: WP_052422984.1 Length: 190
Range 1: 40 to 184
Score:126 bits(317), Expect:3e-33,
Method:Compositional matrix adjust.,
Identities:68/146(47%), Positives:96/146(65%), Gaps:1/146(0%)
Query 39   RVPSLYADPLAWLVADAAESALTQCATEVLAAREDVAVILVSAHCTLHTMDAMARSLPAP 98
R+PSLYADP +W + +A +   C   AA E A++LVSA+ T TM +AR++P
Sbjct 40   RIPSLYADPASWALLEAVDELWAGCPEAAQAA-ETTAMVLVSAYGTAETMSGIARAVPGG 98
Query 99   RVSPLRFAGANPGSAGSLLCLLHGFKGSSLTLSMTPEDGLPAARTVARSWLRSGAASFVV 158
R+SP+RFAGA+PG   SL+C++HG +G +L ++ P +G+PAART+AR WLR+GAAS VV
Sbjct 99   RLSPMRFAGASPGGPISLVCMVHGLRGPTLVITTDPAEGMPAARTMARHWLRTGAASQVV 158

Query 159 LCTHRGHGEKGHSVRCAVLAPAAGRR 184
+ H +GH V C + PA R
Sbjct 159 VAVHHHDPLRGHEVWCVMAGPAPALR 184
>hypothetical protein [Pseudomonas psychrotolerans]
Sequence ID: WP_058767951.1 Length: 177
Range 1: 34 to 174
Score:125 bits(314), Expect:6e-33,
Method:Compositional matrix adjust.,
Identities:73/142(51%), Positives:88/142(61%), Gaps:1/142(0%)
Query 42 SLYADPLAWLVADAAESALTQCATEVLAAREDVAVILVSAHCTLHTMDAMARSLPAPRVS 101
S YADPL+WLV +A E AL C VL A + V I+VS CTLHTM +AR L R+S
Sbjct 34 SAYADPLSWLVFEAVERALDSCRDAVLTAGQTVGHIVVSDVCTLHTMQHIARDLGRNRLS 93
Query 102 PLRFAGANPGSAGSLLCLLHGFKGSSLTLSMTPEDGLPAARTVARSWLRSGAASFVVLCT 161
PLRF+GA PG SL L GF G SL LSM P+DGL A +A+ WL SGAAS V++
Sbjct 94 PLRFSGACPGLVCSLPGQLLGFSGPSLVLSMPPQDGLVPAALLAQDWLDSGAASHVLVSA 153
Query 162 HRGHGEKGHSVRCAVLAPAAGR 183
H G + H +RC +L P R
Sbjct 154 HELAGTQ-HRIRCTLLQPQTER 174

>hypothetical protein [Actinoalloteichus hymeniacidonis]
Sequence ID: WP_069846947.1 Length: 190
Range 1: 40 to 184
Score:124 bits(312), Expect:1e-32,
Method:Compositional matrix adjust.,
Identities:67/146(46%), Positives:94/146(64%), Gaps:1/146(0%)
Query 39 RVPSLYADPLAWLVADAAESALTQCATEVLAAREDVAVILVSAHCTLHTMDAMARSLPAP 98
R+PS YADP +W V AA L + +VL A A+IL+S + T+ TM A+A ++P+
Sbjct 40 RIPSFYADPASWTVL-AAVDRLIEGRPDVLQAARTTALILISTYSTVSTMSAIAAAVPSG 98
Query 99 RVSPLRFAGANPGSAGSLLCLLHGFKGSSLTLSMTPEDGLPAARTVARSWLRSGAASFVV 158
RVSPLRFAGAN G SL+C++HG +G +L L+ P +G+PAA T+AR WLR+ + S VV
Sbjct 99 RVSPLRFAGANAGGPLSLVCMVHGLRGPTLVLTTEPAEGVPAAVTMARHWLRTASVSHVV 158
Query 159 LCTHRGHGEKGHSVRCAVLAPAAGRR 184
L H ++GH V ++ A G R
Sbjct 159 LAVHDHDPQRGHHVHGVLIEAARGLR 184
>coronafacic acid synthetase [Xanthomonas arboricola]
Sequence ID: WP_184622999.1 Length: 177

Range 1: 34 to 174
Score:123 bits(308), Expect:4e-32,
Method:Compositional matrix adjust.,
Identities:72/142(51%), Positives:88/142(61%), Gaps:1/142(0%)
Query 42 SLYADPLAWLVADAAESALTQCATEVLAAREDVAVILVSAHCTLHTMDAMARSLPAPRVS 101
S YADPL+WLV +A E AL C VL A + V I+VS CTLHTM +AR L R+S
Sbjct 34 SAYADPLSWLVFEAVERALDNCRDAVLTACQTVGHIVVSDICTLHTMQHIARDLGRNRLS 93
Query 102 PLRFAGANPGSAGSLLCLLHGFKGSSLTLSMTPEDGLPAARTVARSWLRSGAASFVVLCT 161
PLRF+GA PG SL L F G S+ LSM P+DGL A +A+ WL SGAA +V++
Sbjct 94 PLRFSGACPGLVCSLPGQLLRFSGPSMVLSMPPQDGLVPAALLAQDWLDSGAARYVLVSA 153
Query 162 HRGHGEKGHSVRCAVLAPAAGR 183
H G + H VRC +L P A R
Sbjct 154 HELAGTQ-HRVRCTLLKPQAER 174
>hypothetical protein [Streptomyces sp. OM5714]
Sequence ID: WP_164386746.1 Length: 190
Range 1: 40 to 180
Score:123 bits(308), Expect:5e-32,
Method:Compositional matrix adjust.,
Identities:72/149(48%), Positives:89/149(59%), Gaps:15/149(10%)

Query 39 RVPSLYADPLAWLVADAAESALTQCATEVLAARED-------VAVILVSAHCTLHTMDAM 91
R+PSLYADP AW AL + E+ A R D A ILVS H TL T+ +
Sbjct 40 RIPSLYADPAAW--------ALLEAVDELWAGRPDEPREPETTATILVSTHGTLDTVAGI 91
Query 92 ARSLPAPRVSPLRFAGANPGSAGSLLCLLHGFKGSSLTLSMTPEDGLPAARTVARSWLRS 151
AR++P R+S LRFAGA G+ SL+C++HG +G SLTL+ P GLPAA T AR WLR+
Sbjct 92 ARAVPGGRLSALRFAGAGAGAPVSLVCMVHGLRGPSLTLTTDPARGLPAALTTARHWLRT 151
Query 152 GAASFVVLCTHRGHGEKGHSVRCAVLAPA 180
GAA VV+ H GH VRC V+ A
Sbjct 152 GAAGHVVVAVHEHDPLHGHDVRCVVVGAA 180
>coronafacic acid synthetase component [Kribbella antibiotica]
Sequence ID: WP_165956521.1 Length: 153
Range 1: 19 to 149
Score:120 bits(302), Expect:2e-31,
Method:Compositional matrix adjust.,
Identities:67/139(48%), Positives:90/139(64%), Gaps:8/139(5%)
Query 39 RVPSLYADPLAWLVADAAESALTQCATEVLAAREDVAVILVSAHCTLHTMDAMARSLPAP 98
R+PS+Y DP+AWLV ++ E A++ +DV V+++S + TL TM ++ + +
Sbjct 19 RIPSMYVDPVAWLVLESIEQAISH------PLPDDVGVLVISTYATLATMTELSGTAASG 72

Query 99 RVSPLRFAGANPGSAGSLLCLLHGFKGSSLTLSMTPEDGLPAARTVARSWLRSGAASFVV 158
RVSPLRFAGA+PG A SL CL+HGF+G SL L+ P   P A TVA+SWL SGAAS VV
Sbjct 73 RVSPLRFAGASPGGAASLACLVHGFRGPSLLLTSDPARSRPVALTVAQSWLSSGAASCVV 132
Query 159 LCTHRGHGEKGHSVRCAVL 177
L H   + GH+V   VL
Sbjct 133 LSVHT--VDSGHAVHTVVL 149
>polyketide synthase [Streptomyces griseocarneus]
Sequence ID: RLV10181.1 Length: 171
Range 1: 29 to 171
Score:120 bits(301), Expect:4e-31,
Method:Compositional matrix adjust.,
Identities:69/144(48%), Positives:84/144(58%), Gaps:1/144(0%)
Query 41 PSLYADPLAWLVADAAESALTQCATEVLAAREDVAVILVSAHCTLHTMDAMARSLPAPRV 100
PS YADP AWLVA+ + AL CA VL A +D ++++SA + TM A+ARS+P RV
Sbjct 29 PSFYADPAAWLVAETVDRALADCAEPVLDAADDTGIVVMSATGSERTMRAIARSVPRSRV 88
Query 101 SPLRFAGANPGSAGSLLCLLHGFKGSSLTLSMTPEDGLPAARTVARSWLRSGAASFVVLC 160
SPLRFAGANPG   L L HG +G SL L+M P+   P A TV   WL G A V+L
Sbjct 89 SPLRFAGANPGVLAGLPALRHGLRGPSLLLAMHPDAATPVALTVIDGWLADGQARHVLLA 148
Query 161 THRGHGEKGHSVRCAVLAPAAGRR 184

R + GH C VL A R
Sbjct 149 GLRSTAD-GHECHCQVLTGAGADR 171
>MULTISPECIES: polyketide synthase [Streptomyces]
Sequence ID: WP_171079069.1 Length: 172
Range 1: 1 to 167
Score:119 bits(299), Expect:8e-31,
Method:Compositional matrix adjust.,
Identities:83/176(47%), Positives:98/176(55%), Gaps:9/176(5%)
Query 1 MNVFPASEPYVIFPPDTVDVAVVGHGDAFREELTAVRSRVPSLYADPLAWLVADAAESAL 60
MN PA+ P+V P + A V D EE + PS YADP+AWL +AA AL
Sbjct 1 MN--PAAVPFVAPPESVLARAEVTSTDP--EEFS---RNAPSTYADPVAWLAVEAARRAL 53
Query 61 TQCATEVLAAREDVAVILVSAHCTLHTMDAMARSLPAPRVSPLRFAGANPGSAGSLLCLL 120
EVLAARED AV+ VSAH T+ TM + R + A RVSPL FAGANPG +C
Sbjct 54 ADV-PEVLAAREDTAVLAVSAHATIGTMHGIVRRIRAGRVSPLHFAGANPGLLAGAVCRE 112
Query 121 HGFKGSSLTLSMTPEDGLPAARTVARSWLRSGAASFVVLCTHRGHGEKGHSVRCAV 176
KG SLTL+M P G AA TVAR L A +V+L TH E GH+ C V
Sbjct 113 WQLKGPSLTLTMPPAHGTDAALTVARGLLGRALAPYVLLLTHEAD-EAGHTAVCTV 167

>Coronafacic acid synthetase component [Pseudomonas syringae pv. maculicola]
Sequence ID: KPB94666.1 Length: 158
Range 1: 15 to 149
Score:119 bits(297), Expect:1e-30,
Method:Compositional matrix adjust.,
Identities:70/136(51%), Positives:85/136(62%), Gaps:1/136(0%)
Query 42 SLYADPLAWLVADAAESALTQCATEVLAAREDVAVILVSAHCTLHTMDAMARSLPAPRVS 101
S YADPL+WLV +A + L C VLAA + V I+VS CTLHTM +AR L R+S
Sbjct 15 SAYADPLSWLVFEAVDYVLDNCRDAVLAAGQTVGHIVVSDVCTLHTMQHIARDLGRNRLS 74
Query 102 PLRFAGANPGSAGSLLCLLHGFKGSSLTLSMTPEDGLPAARTVARSWLRSGAASFVVLCT 161
PLRF+GA PG SL L F G SL LSM P+ GL A +AR WL SGAAS+V++
Sbjct 75 PLRFSGACPGLVCSLPGQLLRFSGPSLVLSMPPQGGLRPAALLARDWLDSGAASYVLVSA 134
Query 162 HRGHGEKGHSVRCAVL 177
H G + H VRC +L
Sbjct 135 HDTDGAQ-HRVRCTLL 149
>hypothetical protein [Streptomyces sp. F-1]
Sequence ID: WP_070024088.1 Length: 201
Range 1: 9 to 172

Score:120 bits(300), Expect:1e-30,
Method:Compositional matrix adjust.,
Identities:79/165(48%), Positives:102/165(61%), Gaps:7/165(4%)
Query 19 DVAVVGHGDAFREELTAVRSRVPSLYADPLAWLVADAAESALTQCA---TEV--LAARED 73
D+A VG+ + + SL+ADPLAWL+A+AAE+A C TE+ L+ D
Sbjct 9 DLASVGYAEIVTDAPERHSRNGSSLFADPLAWLMAEAAEAAARDCRPGLTEIGDLSEIGD 68
Query 74 -VAVILVSAHCTLHTMDAMARSLPAPRVSPLRFAGANPGSAGSLLCLLHGFKGSSLTLSM 132
V VI +S CTL TM +AR+ P R+SPL+FAGANPGS L C+ GF+G +LTLSM
Sbjct 69 LVGVIAISEVCTLDTMRQIARATPRGRLSPLKFAGANPGSLAGLACIRQGFRGPTLTLSM 128
Query 133 TPEDGLPAARTVARSWLRSGAASFVVLCTHRGHGEKGHSVRCAVL 177
P LP A VA W G+A +V+L THR G+ H+VRCAVL
Sbjct 129 PPVAALPTALDVAAGWFGRGSARYVLLGTHRQDGDT-HAVRCAVL 172
>coronafacic acid synthetase component [Kribbella sandramycini]
Sequence ID: WP_171675246.1 Length: 137
Range 1: 1 to 125
Score:117 bits(294), Expect:2e-30,
Method:Compositional matrix adjust.,
Identities:64/132(48%), Positives:84/132(63%), Gaps:7/132(5%)
Query 33 LTAVRSRVPSLYADPLAWLVADAAESALTQCATEVLAAREDVAVILVSAHCTLHTMDAMA 92

+T+ R R+PS+Y D +AWLV ++ E +T+ E D V+++S H T TM+ +A
Sbjct 1 MTSTR-RIPSMYVDQVAWLVLESIERVITEPLPE------DTGVLVLSTHATRDTMEQLA 53
Query 93 RSLPAPRVSPLRFAGANPGSAGSLLCLLHGFKGSSLTLSMTPEDGLPAARTVARSWLRSG 152
++SPLRFAGANPG+ L CL G +G SL L+ PE LP A+ VARSWLRSG
Sbjct 54 GMAQNGQISPLRFAGANPGAIAGLPCLRLGLRGPSLLLTSDPERSLPVAQVVARSWLRSG 113
Query 153 AASFVVLCTHRG 164
AAS VVL HRG
Sbjct 114 AASRVVLSVHRG 125
>MULTISPECIES: hypothetical protein [Pseudomonas syringae group]
Sequence ID: WP_054990124.1 Length: 177
Range 1: 34 to 168
Score:118 bits(296), Expect:3e-30,
Method:Compositional matrix adjust.,
Identities:70/136(51%), Positives:85/136(62%), Gaps:1/136(0%)
Query 42 SLYADPLAWLVADAAESALTQCATEVLAAREDVAVILVSAHCTLHTMDAMARSLPAPRVS 101
S YADPL+WLV +A + L C VLAA + V I+VS CTLHTM +AR L R+S
Sbjct 34 SAYADPLSWLVFEAVDYVLDNCRDAVLAAGQTVGHIVVSDVCTLHTMQHIARDLGRNRLS 93
Query 102 PLRFAGANPGSAGSLLCLLHGFKGSSLTLSMTPEDGLPAARTVARSWLRSGAASFVVLCT 161
PLRF+GA PG SL L F G SL LSM P DGL A +A+ WL SGAAS+V++

Sbjct 94 PLRFSGACPGLVCSLPGQLLRFSGPSLVLSMPPLDGLRPAALLAQDWLDSGAASYVLVSA 153
Query 162 HRGHGEKGHSVRCAVL 177
H G + H VRC +L
Sbjct 154 HDTDGAQ-HRVRCTLL 168
>hypothetical protein [Nonomuraea pusilla]
Sequence ID: WP_177227589.1 Length: 580
Range 1: 393 to 551
Score:125 bits(314), Expect:4e-30,
Method:Compositional matrix adjust.,
Identities:79/160(49%), Positives:100/160(62%), Gaps:4/160(2%)
Query 21 AVVGHGDAFREELTAVRSRVPSLYADPLAWLVADAAESALTQ--CATEVLAAREDVAVIL 78
AVVGHG+ E+ A + P+LYADP+AWLVA A E+ L + C +VLAAR DVAVI+
Sbjct 393 AVVGHGELLSEDPAARSADRPALYADPVAWLVAGAVEAVLDESGCGPDVLAARNDVAVIV 452
Query 79 VSAHCTLHTMDAMARSLPAP-RVSPLRFAGANPGSAGSLLCLLHGFKGSSLTLSMTPEDG 137
++ L T A+A + A RVSP RFAGANPG L C+ G +G SL L +
Sbjct 453 LTGTQPLPTSSALAAQVAAQGRVSPRRFAGANPGILAGLTCIRWGLRGPSLVLEAADDGA 512
Query 138 LPAARTVARSWLRSGAASFVVLCTHRGHGEKGHSVRCAVL 177
+ AA TVA SWL SG A +V+ H+ H GH+VRCAVL
Sbjct 513 VDAALTVAESWLGSGQARYVIAVRHQTH-SGGHTVRCAVL 551

>coronafacic acid synthetase [Lonsdalea iberica]
Sequence ID: WP_094109901.1 Length: 178
Range 1: 11 to 169
Score:117 bits(294), Expect:6e-30,
Method:Compositional matrix adjust.,
Identities:67/160(42%), Positives:95/160(59%), Gaps:2/160(1%)
Query 19   DVAVVGHGDAFREELTAVRS-RVPSLYADPLAWLVADAAESALTQCATEVLAAREDVAVI 77
D+ +G+G A   +L A+++ R SLYADPLAW+V DA E AL +   + AA+ V I
Sbjct 11   DLPAIGYGHAVAADLAAMKAGRKASLYADPLAWMVYDAVELALEKDREAICAAKRTVGHI 70
Query 78   LVSAHCTLHTMDAMARSLPAPRVSPLRFAGANPGSAGSLLCLLHGFKGSSLTLSMTPEDG 137
++S CT HT+ + ++ + R+SPLRF+GA PG   +L   GF G S+ LSM E G
Sbjct 71   VISDQCTAHTLREIGAAISSGRISPLRFSGACPGLVCALPGQFLGFNGPSMVLSMPAEQG 130
Query 138 LPAARTVARSWLRSGAASFVVLCTHRGHGEKGHSVRCAVL 177
LPAA +AR+WL   AAS V++ H   GH+V   +
Sbjct 131 LPAAAAIARNWLSEHAASHVIVTCHEA-DPAGHTVTSVIF 169
>MULTISPECIES: coronafacic acid synthetase [unclassified Hazenella]

Sequence ID: WP_191140370.1 Length: 197
Range 1: 18 to 177
Score:117 bits(294), Expect:9e-30,
Method:Compositional matrix adjust.,
Identities:55/160(34%), Positives:93/160(58%), Gaps:0/160(0%)
Query 19 DVAVVGHGDAFREELTAVRSRVPSLYADPLAWLVADAAESALTQCATEVLAAREDVAVIL 78
+ + G + ++LT + +V S YADP AWLV DA + AL + ++ +E + VI
Sbjct 18 QIKICSFGMSHTDKLTHAQRKVASFYADPAAWLVVDAVKDALQRFNRDLTPIKEKIGVIT 77
Query 79 VSAHCTLHTMDAMARSLPAPRVSPLRFAGANPGSAGSLLCLLHGFKGSSLTLSMTPEDGL 138
+S CTL+T++ + + RVSP+RFAGANPGS L C++HG++G +L + + +
Sbjct 78 ISDICTLNTINMIRNQVSKGRVSPIRFAGANPGSMAGLPCIIHGYRGPTLVFTSPLAETI 137
Query 139 PAARTVARSWLRSGAASFVVLCTHRGHGEKGHSVRCAVLA 178
A SWLR AS+V + T++ + ++VR +++
Sbjct 138 DAVVATVLSWLRKQQASYVFINTYQQQSTECYTVRSILVS 177
>hypothetical protein [Lonsdalea quercina]
Sequence ID: WP_051616280.1 Length: 186
Range 1: 15 to 169
Score:117 bits(292), Expect:1e-29,
Method:Compositional matrix adjust.,

Identities:66/156(42%), Positives:92/156(58%), Gaps:2/156(1%)
Query 23 VGHGDAFREELTAVRS-RVPSLYADPLAWLVADAAESALTQCATEVLAAREDVAVILVSA 81
+G G A +L A+++ R SLYADPLAWLV DA E AL + + AA+ V I +S
Sbjct 15 IGFGRATATDLAAMKAGRKASLYADPLAWLVYDAVELALEESREAICAAKRTVGHIAISD 74
Query 82 HCTLHTMDAMARSLPAPRVSPLRFAGANPGSAGSLLCLLHGFKGSSLTLSMTPEDGLPAA 141
CT HT+ + ++ + R+SPLRF+GA PGS +L GF G S+ LSM E GLP A
Sbjct 75 QCTAHTLREIGTTISSGRISPLRFSGACPGSVCALPGQFLGFNGPSMVLSMPAEQGLPVA 134
Query 142 RTVARSWLRSGAASFVVLCTHRGHGEKGHSVRCAVL 177
+A+ WLR AAS +++ H + GH+V +
Sbjct 135 VAIAKIWLREQAASHMIVTCHEANA-AGHTVTSVIF 169
>coronafacic acid synthetase [Lonsdalea britannica]
Sequence ID: WP_085653152.1 Length: 178
Range 1: 9 to 169
Score:116 bits(290), Expect:2e-29,
Method:Compositional matrix adjust.,
Identities:69/162(43%), Positives:93/162(57%), Gaps:2/162(1%)
Query 17 TVDVAVVGHGDAFREELTAVRS-RVPSLYADPLAWLVADAAESALTQCATEVLAAREDVA 75
T + VG G A E+L A+++ R SLYADPLAWLV DA E AL + + AA+ V
Sbjct 9 TCGLPAVGFGRAVAEDLAAMKTGRKASLYADPLAWLVYDAVELALEEDREAICAAKRTVG 68

Query 76 VILVSAHCTLHTMDAMARSLPAPRVSPLRFAGANPGSAGSLLCLLHGFKGSSLTLSMTPE 135
I +S CT HT+ + + + R+SPLRF+GA PG +L GF G S+ LSM E
Sbjct 69 HIAISDQCTAHTLREIGAVIASGRISPLRFSGACPGLVCALPGQFLGFNGPSMVLSMPAE 128
Query 136 DGLPAARTVARSWLRSGAASFVVLCTHRGHGEKGHSVRCAVL 177
GLPAA +A++WL AAS V++ H GH+V +
Sbjct 129 QGLPAAAAIAKTWLSEHAASHVIVTCHEADA-AGHTVTSVIF 169
>hypothetical protein [Streptomyces sp. NRRL F-4489]
Sequence ID: WP_066982330.1 Length: 200
Range 1: 2 to 174
Score:116 bits(291), Expect:3e-29,
Method:Compositional matrix adjust.,
Identities:71/174(41%), Positives:102/174(58%), Gaps:1/174(0%)
Query 7 SEPYVIFPPDTVDVAVVGHGDAFREELTAVRSRVPSLYADPLAWLVADAAESALTQCATE 66
S+P P + G+G+ + A SL+ADP+AWL+ +AA A C T+
Sbjct 2 SDPRPTAPATRRPLITTGYGEVVTTDPAAHSRNGSSLFADPVAWLMTEAAARAAADCDTD 61
Query 67 VLAAREDVAVILVSAHCTLHTMDAMARSLPAPRVSPLRFAGANPGSAGSLLCLLHGFKGS 126
+ AA + V +I +S CT+ TM +AR+ P R+SPL+FAGANPGS L C+ +GF+G
Sbjct 62 LTAAGDQVGMIAISEICTMDTMRTIARATPRGRLSPLKFAGANPGSVAGLPCIRNGFRGP 121

Query 127 SLTLSMTPEDGLPAARTVARSWLRSGAASFVVLCTHRGHGEKGHSVRCAVLAPA 180
+L LSM P   LPAA +A W+ G+A +V++ HR G+ H+ RC VL A
Sbjct 122 TLALSMPPGPALPAALAMAEGWIAQGSARYVLIGAHRRDGD-AHAARCHVLQSA 174
>polyketide synthase [Actinobacteria bacterium]
Sequence ID: TMM37893.1 Length: 194
Range 1: 31 to 165
Score:115 bits(289), Expect:4e-29,
Method:Compositional matrix adjust.,
Identities:65/136(48%), Positives:82/136(60%), Gaps:1/136(0%)
Query 41   PSLYADPLAWLVADAAESALTQCATEVLAAREDVAVILVSAHCTLHTMDAMARSLPAPRV 100
PS YADP WLVA A + A   CA E L+ ++V V+ VSA CT HTM A+A S   V
Sbjct 31   PSFYADPAGWLVAAAVQEAFDGCAGEPLSVPDEVGVVAVSAACTAHTMGAIATSARDGLV 90
Query 101 SPLRFAGANPGSAGSLLCLLHGFKGSSLTLSMTPEDGLPAARTVARSWLRSGAASFVVLC 160
SPLRFAGA+PG   L C+   +G +LTL+M P GL A TVA WLR G A +V++
Sbjct 91   SPLRFAGASPGILAGLACIRWKLRGPTLTLAMDPVAGLDVAATVAGGWLRDGQARYVLVA 150
Query 161 THRGHGEKGHSVRCAV 176
+   ++ H RCAV
Sbjct 151 AYTVEDDQ-HVARCAV 165

>Coronafacic acid beta-ketoacyl synthetase component [Pseudomonas amygdali pv. aesculi]
Sequence ID: KPW22679.1 Length: 601
Range 1: 458 to 592
Score:121 bits(303), Expect:2e-28,
Method:Compositional matrix adjust.,
Identities:71/136(52%), Positives:86/136(63%), Gaps:1/136(0%)
Query 42   SLYADPLAWLVADAAESALTQCATEVLAAREDVAVILVSAHCTLHTMDAMARSLPAPRVS 101
S YADPL+WLV +A E L C   VLAA + V I+VS CTLHTM +AR L   R+S
Sbjct 458 SAYADPLSWLVFEAVECVLDSCRDAVLAAGQTVGHIVVSDVCTLHTMQHIARDLGRNRLS 517
Query 102 PLRFAGANPGSAGSLLCLLHGFKGSSLTLSMTPEDGLPAARTVARSWLRSGAASFVVLCT 161
PLRF+GA PG   SL   L F G S+ LSM P+DGL A +A+ WL SGAAS V++ T
Sbjct 518 PLRFSGACPGLVCSLAGQLLHFSGPSMVLSMPPQDGLVPAALLAQDWLDSGAASHVLVST 577
Query 162 HRGHGEKGHSVRCAVL 177
H   G + H VRC +L
Sbjct 578 HDTDGTQ-HRVRCTLL 592
>coronafacic acid synthetase [Brenneria sp. CFCC 11842]
Sequence ID: WP_136167060.1 Length: 178
Range 1: 8 to 170

Score:113 bits(283), Expect:3e-28,
Method:Compositional matrix adjust.,
Identities:66/164(40%), Positives:96/164(58%), Gaps:2/164(1%)
Query 16 DTVDVAVVGHGDAFREELTAVRSRVP-SLYADPLAWLVADAAESALTQCATEVLAAREDV 74
+ +A++G G A +L++++ + SLYADPLAWLV +A E AL + ++ + ++ V
Sbjct 8 NACGLAILGRGVATAPDLSSLKPKQKASLYADPLAWLVLEAVEQALKENSSALASSGLAV 67
Query 75 AVILVSAHCTLHTMDAMARSLPAPRVSPLRFAGANPGSAGSLLCLLHGFKGSSLTLSMTP 134
I VS CT HT+ + ++P+ R+SPLRF+GA PG L L GF G SL LSM P
Sbjct 68 GHIAVSDQCTAHTLHGIGTTIPSGRISPLRFSGACPGMICCLPSLFLGFSGPSLVLSMPP 127
Query 135 EDGLPAARTVARSWLRSGAASFVVLCTHRGHGEKGHSVRCAVLA 178
GLP A +A WL +A+ V++ HR GH V +LA
Sbjct 128 ASGLPPALAIASIWLHECSATHVIVTYHR-KDTSGHGVTSLILA 170
>hypothetical protein [Xanthomonas campestris]
Sequence ID: WP_228321346.1 Length: 177
Range 1: 11 to 171
Score:112 bits(281), Expect:4e-28,
Method:Compositional matrix adjust.,
Identities:75/162(46%), Positives:90/162(55%), Gaps:2/162(1%)

Query 20 VAVVGHGDAFRE-ELTAVRSRVPSLYADPLAWLVADAAESALTQCATEVLAAREDVAVIL 78
V V+G G A E L +++ S YADPLAWL+ DA E L C VLAA V I+
Sbjct 11 VRVLGQGSAETEASLPPRQAQKASAYADPLAWLLLDAVERTLAPCRDAVLAAHATVGQIV 70
Query 79 VSAHCTLHTMDAMARSLPAPRVSPLRFAGANPGSAGSLLCLLHGFKGSSLTLSMTPEDGL 138
VS CTL TM +AR L R+SPLRF+GA PG S+ F G SL LSM PE G+
Sbjct 71 VSDLCTLPTMRHIARDLARNRLSPLRFSGACPGLICSVPAQQFRFNGPSLVLSMPPESGM 130
Query 139 PAARTVARSWLRSGAASFVVLCTHRGHGEKGHSVRCAVLAPA 180
A +AR WL S AAS V+L H GH + C +L A
Sbjct 131 TYAALLARDWLHSAAASHVLLSAHHADA-AGHRMVCTLLQAA 171
>hypothetical protein [Micromonospora sp. KC721]
Sequence ID: WP_132261363.1 Length: 171
Range 1: 25 to 170
Score:112 bits(281), Expect:5e-28,
Method:Compositional matrix adjust.,
Identities:66/147(45%), Positives:78/147(53%), Gaps:1/147(0%)
Query 37 RSRVPSLYADPLAWLVADAAESALTQCATEVLAAREDVAVILVSAHCTLHTMDAMARSLP 96
R R S YADP+AWLVADA + DV V+ VS + T HTM +
Sbjct 25 RHRGASFYADPVAWLVADAVGAVFDAAGQSGKGTGTDVGVLAVSEYGTSHTMREVTAMRD 84
Query 97 APRVSPLRFAGANPGSAGSLLCLLHGFKGSSLTLSMTPEDGLPAARTVARSWLRSGAASF 156

RVSPLRFAGANPGS L C++HG +G SL LSM P P +AR+WL G
Sbjct 85 RGRVSPLRFAGANPGSVAGLACIVHGLRGPSLMLSMPPPAARPTVALLARAWLTGGVCRR 144
Query 157 VVLCTHRGHGEKGHSVRCAVLAPAAGR 183
VV+ H G GH+VR VL A R
Sbjct 145 VVVSEHE-RGPAGHTVRTVVLTSAEAR 170
>polyketide synthase [Streptomyces kasugaensis]
Sequence ID: WP_131124666.1 Length: 172
Range 1: 29 to 172
Score:112 bits(280), Expect:5e-28,
Method:Compositional matrix adjust.,
Identities:65/144(45%), Positives:80/144(55%), Gaps:0/144(0%)
Query 41 PSLYADPLAWLVADAAESALTQCATEVLAAREDVAVILVSAHCTLHTMDAMARSLPAPRV 100
PS YADP AWLVA+ + AL CA VL A +D ++++SA + TM +A S+P RV
Sbjct 29 PSFYADPAAWLVAETVDRALADCAEPVLDAADDTGIVVMSATGSERTMRRIAASVPRSRV 88
Query 101 SPLRFAGANPGSAGSLLCLLHGFKGSSLTLSMTPEDGLPAARTVARSWLRSGAASFVVLC 160
SPLRFAGANPG L L HG +G SL L+ P++ P A TV WL G A V+L
Sbjct 89 SPLRFAGANPGVLAGLPALRHGLRGPSLLLAAHPDEAAPVAFTVIGEWLADGHARHVLLA 148
Query 161 THRGHGEKGHSVRCAVLAPAAGRR 184
G RC VL A R

Sbjct 149 CLYATAGDGQMCRCLVLTGAGADR 172
>hypothetical protein [Pseudonocardia sp. HH130630-07]
Sequence ID: WP_068798146.1 Length: 182
Range 1: 22 to 158
Score:111 bits(278), Expect:2e-27,
Method:Compositional matrix adjust.,
Identities:70/138(51%), Positives:85/138(61%), Gaps:1/138(0%)
Query 41 PSLYADPLAWLVADAAESALTQCATEVLAAREDVAVILVSAHCTLHTMDAMARSLPAPRV 100
PSLYADP+AWL+ + AL C +VL ++V + VS H TL TM A+ARS RV
Sbjct 22 PSLYADPVAWLLCEVTGQALAGCPDDVLHHTDEVGHLAVSDHGTLDTMRALARSGRRGRV 81
Query 101 SPLRFAGANPGSAGSLLCLLHGFKGSSLTLSMTPEDGLPAARTVARSWLRSGAASFVVLC 160
SPLRFAGANPGS L CL G +G ++ L+M P PAA VA+ WL +G A VVL
Sbjct 82 SPLRFAGANPGSLAGLACLRWGLRGPTMMLAMPPSPAGPAAVAVAQRWLDTGQARHVVLA 141
Query 161 THRGHGEKGHSVRCAVLA 178
TH + H RCAVLA
Sbjct 142 THTVR-DGVHEARCAVLA 158

>coronafacic acid synthetase [Brenneria sp. hezel4-2-4]
Sequence ID: WP_172289556.1 Length: 178
Range 1: 35 to 155
Score:110 bits(275), Expect:4e-27,
Method:Compositional matrix adjust.,
Identities:55/121(45%), Positives:76/121(62%), Gaps:0/121(0%)
Query 42 SLYADPLAWLVADAAESALTQCATEVLAAREDVAVILVSAHCTLHTMDAMARSLPAPRVS 101
SLYADPL+WL+ +A E A+ + +L+A V I +S HCTLHTM A+A+++P+ +S
Sbjct 35 SLYADPLSWLILEAVEQAIGEHLDAILSASPSVGHIAISDHCTLHTMRAIAKTIPSGHIS 94
Query 102 PLRFAGANPGSAGSLLCLLHGFKGSSLTLSMTPEDGLPAARTVARSWLRSGAASFVVLCT 161
PLRF+GA PG SL F G S+ SM PE LP A +AR+WL A+ V++
Sbjct 95 PLRFSGACPGMICSLPSQFLKFSGPSIVFSMPPESALPYAAVLARAWLHEHLATHVIITV 154
Query 162 H 162
H
Sbjct 155 H 155
>hypothetical protein [Streptomyces sp. MMG1121]
Sequence ID: WP_053666612.1 Length: 200
Range 1: 15 to 171
Score:110 bits(276), Expect:5e-27,

Method:Compositional matrix adjust.,
Identities:71/158(45%), Positives:97/158(61%), Gaps:1/158(0%)
Query 20 VAVVGHGDAFREELTAVRSRVPSLYADPLAWLVADAAESALTQCATEVLAAREDVAVILV 79
+A G+G+ L SL+ADP+AWL+A+A A+ C ++ AA + VA+I +
Sbjct 15 LAATGYGEVLTTTLAEHTHNGSSLFADPVAWLMAEAVARAVEDCPADLAAAGDHVAMIAI 74
Query 80 SAHCTLHTMDAMARSLPAPRVSPLRFAGANPGSAGSLLCLLHGFKGSSLTLSMTPEDGLP 139
S CTL TM +AR+ P R+SPL+FAGANPGS L C+ GF+G +LTLSM PE LP
Sbjct 75 SDVCTLDTMRTIARATPRGRLSPLKFAGANPGSVAGLPCIRGGFRGPTLTLSMPPEAALP 134
Query 140 AARTVARSWLRSGAASFVVLCTHRGHGEKGHSVRCAVL 177
AA +A W+ G+A FV++ HR G+ H R VL
Sbjct 135 AALAMAEGWIARGSARFVLVGAHRVDGD-AHGARACVL 171
>polyketide synthase [Streptomyces sp. CB02959]
Sequence ID: WP_100602528.1 Length: 172
Range 1: 5 to 172
Score:109 bits(272), Expect:1e-26,
Method:Compositional matrix adjust.,
Identities:70/171(41%), Positives:87/171(50%), Gaps:3/171(1%)
Query 14 PPDTVDVAVVGHGDAFREELTAVRSRVPSLYADPLAWLVADAAESALTQCATEVLAARED 73
PP T V+G E+ PS YADP AWLVA+ + ALT CA VL A +D

Sbjct 5 PPGT---RVLGSATVVAEDPAEYAINKPSFYADPAAWLVAETVDRALTDCAERVLDAADD 61
Query 74 VAVILVSAHCTLHTMDAMARSLPAPRVSPLRFAGANPGSAGSLLCLLHGFKGSSLTLSMT 133
A++++SA + T+ +A S+P RVSPLRFAGANPG L L HG +G SL L+
Sbjct 62 TAILVMSATGSERTIRRIAASVPRSRVSPLRFAGANPGVLAGLPALRHGLRGPSLLLAAH 121
Query 134 PEDGLPAARTVARSWLRSGAASFVVLCTHRGHGEKGHSVRCAVLAPAAGRR 184
P+ P A TV WL G A V+L C VL A R
Sbjct 122 PDQAAPVAFTVIDGWLADGHARHVILVGLESAAGDRERCCCQVLTSAGEDR 172
>polyketide synthase [Streptomyces albulus]
Sequence ID: WP_038524297.1 Length: 172
Range 1: 29 to 172
Score:108 bits(271), Expect:1e-26,
Method:Compositional matrix adjust.,
Identities:67/145(46%), Positives:83/145(57%), Gaps:2/145(1%)
Query 41 PSLYADPLAWLVADAAESALTQCATEVLAAREDVAVILVSAHCTLHTMDAMARSLPAPRV 100
PS YADP+AWLVA+ + AL CA V +D A+++VSA + TM +A S+P RV
Sbjct 29 PSFYADPVAWLVAETVDRALADCAEHVRDDADDTAILVVSATGSERTMRRIADSVPRSRV 88
Query 101 SPLRFAGANPGSAGSLLCLLHGFKGSSLTLSMTPEDGLPAARTVARSWLRSGAASFVVLC 160
SPLRFAGANPG L L HG +G SL L+ P+ P A TV WLR G A V+L
Sbjct 89 SPLRFAGANPGVLAGLPALRHGLRGPSLLLAGHPDAAAPVAGTVIAGWLRDGHARHVLLV 148

Query 161 -THRGHGEKGHSVRCAVLAPAAGRR 184
H GE+ + C VL A R
Sbjct 149 GLHATEGER-ETCCCLVLTGAGADR 172
>MULTISPECIES: hypothetical protein [unclassified Streptomyces]
Sequence ID: WP_018537882.1 Length: 172
Range 1: 3 to 172
Score:108 bits(271), Expect:1e-26,
Method:Compositional matrix adjust.,
Identities:70/173(40%), Positives:88/173(50%), Gaps:3/173(1%)
Query 12 IFPPDTVDVAVVGHGDAFREELTAVRSRVPSLYADPLAWLVADAAESALTQCATEVLAAR 71
+ PP T V+G E+ A PS YADP AWLVA + ALT CA VL A
Sbjct 3 LVPPGT---RVLGAATLVAEDPAAYAGNKPSFYADPAAWLVAATVDRALTDCAAPVLDAA 59
Query 72 EDVAVILVSAHCTLHTMDAMARSLPAPRVSPLRFAGANPGSAGSLLCLLHGFKGSSLTLS 131
+D A++++SA + TM +A S+P R+SPLRFAGANPG L L HG +G SL L+
Sbjct 60 DDTAILVMSATGSARTMRRIAGSVPRSRISPLRFAGANPGVLAGLPALRHGLRGPSLLLA 119
Query 132 MTPEDGLPAARTVARSWLRSGAASFVVLCTHRGHGEKGHSVRCAVLAPAAGRR 184
P+ P A T+ W G A V+L E R VL A R
Sbjct 120 AHPDTAAPVAFTIIDGWFADGHARHVILVGLHTTAEDRELCRSLVLTRAGEDR 172

>hypothetical protein [Kutzneria sp. CA-103260]
Sequence ID: WP_211763030.1 Length: 162
Range 1: 18 to 148
Score:108 bits(269), Expect:2e-26,
Method:Compositional matrix adjust.,
Identities:65/137(47%), Positives:75/137(54%), Gaps:6/137(4%)
Query 41 PSLYADPLAWLVADAAESALTQCATEVLAAREDVAVILVSAHCTLHTMDAMARSLPAPRV 100
PS YADP+AWLVA A E LT+ R+ VAV+++SA T TM +A RV
Sbjct 18 PSFYADPVAWLVARAVEQTLTEPVE-----RDQVAVLVMSATTTRPTMAGIADKAARGRV 72
Query 101 SPLRFAGANPGSAGSLLCLLHGFKGSSLTLSMTPEDGLPAARTVARSWLRSGAASFVVLC 160
SPLRFAGANPG L C+ GF G SL L+ P D L A V SWL SG A VV
Sbjct 73 SPLRFAGANPGILAGLTCIQQGFTGPSLVLANEPADALDTAAAVVDSWLDSGQARHVVCV 132
Query 161 THRGHGEKGHSVRCAVL 177
HR GH R V+
Sbjct 133 AHRAD-PNGHQARAVVV 148
>hypothetical protein [Zymobacter palmae]
Sequence ID: WP_027704559.1 Length: 178

Range 1: 7 to 169
Score:108 bits(270), Expect:2e-26,
Method:Compositional matrix adjust.,
Identities:68/166(41%), Positives:97/166(58%), Gaps:4/166(2%)
Query 13   FPPDTVDVAVVGHGDAFREELTAVR-SRVPSLYADPLAWLVADAAESALTQCATEVLAAR 71
F P T+   +G G A   +L +++ ++ SLYADPL+WLV A E+A+   + A+R
Sbjct 7   FNPHTL--LAIGEGTASAPDLASMKPTQKASLYADPLSWLVLSAVENAVASTREALDASR 64
Query 72   EDVAVILVSAHCTLHTMDAMARSLPAPRVSPLRFAGANPGSAGSLLCLLHGFKGSSLTLS 131
V I++S CTLHT+ A+AR++P+ VSPL+F+GA PG   L   L GF G S+ LS
Sbjct 65   RTVGHIVISDTCTLHTLHAIARTVPSGHVSPLKFSGACPGLICCLPSQLLGFSGPSIALS 124
Query 132 MTPEDGLPAARTVARSWLRSGAASFVVLCTHRGHGEKGHSVRCAVL 177
M   G P A T+A++WL+ AAS V++ H   GH V   V
Sbjct 125 MPGLLGQPHAMTLAQTWLKDQAASHVIVTVHHADAS-GHRVTSTVF 169
>MULTISPECIES: hypothetical protein [Pectobacterium]
Sequence ID: WP_039313139.1 Length: 177
Range 1: 14 to 176
Score:108 bits(270), Expect:2e-26,
Method:Compositional matrix adjust.,
Identities:61/164(37%), Positives:93/164(56%), Gaps:2/164(1%)

Query 22 VVGHGDAFREELTAVR-SRVPSLYADPLAWLVADAAESALTQCATEVLAAREDVAVILVS 80
+VGHG A +L + ++ SLYADPL+WLV +A E A+ + + +A V I +S
Sbjct 14 IVGHGMASTPDLYTYKPAQKASLYADPLSWLVLEAVEQAINEHRDVITSACSSVGHIAIS 73
Query 81 AHCTLHTMDAMARSLPAPRVSPLRFAGANPGSAGSLLCLLHGFKGSSLTLSMTPEDGLPA 140
CTL T+ ++++LP+ +SPLRF+GA PG L F G S+ SM P++ P
Sbjct 74 DQCTLKTIHDISQTLPSGHLSPLRFSGACPGMIIGLPSQFLRFSGPSIVWSMPPDNMQPY 133
Query 141 ARTVARSWLRSGAASFVVLCTHRGHGEKGHSVRCAVLAPAAGRR 184
A +A+ WL G+A+ V++ HR E GH +R V+ + G
Sbjct 134 ATALAQLWLDEGSATHVIITEHRV-DESGHHIRSVVVTQSGGEH 176
>hypothetical protein [Azospirillum sp. B510]
Sequence ID: WP_052293700.1 Length: 176
Range 1: 12 to 172
Score:108 bits(269), Expect:3e-26,
Method:Compositional matrix adjust.,
Identities:66/165(40%), Positives:88/165(53%), Gaps:6/165(3%)
Query 20 VAVVGHGDAFREELTAVR--SRVPSLYADPLAWLVADAAESALTQCATEVLAAREDVAVI 77
+ ++G G A L V+ R+PSLYADPL+WLV +A A+T D+ I
Sbjct 12 MTILGEGTARASSLQVVKPSRRIPSLYADPLSWLVLEAVTDAVTAAGP---CLDRDLGHI 68

Query 78 LVSAHCTLHTMDAMARSLPAPRVSPLRFAGANPGSAGSLLCLLHGFKGSSLTLSMTPEDG 137
+VS CTLHTM +A ++P R+SPLRF+GANPG L +G SL LSMTP G
Sbjct 69 VVSDVCTLHTMRGLAGAIPNGRMSPLRFSGANPGQICGFAMQLLQLRGPSLVLSMTPSQG 128
Query 138 LPAARTVARSWLRSGAASFVVLCTHRGHGEKGHSVRCAVLAPAAG 182
A V R+WLR A+ V+L H ++ H V + G
Sbjct 129 RHPAMIVLRNWLRERTAAAVILSLHEWDSDE-HRVTSTIFGTTDG 172
>hypothetical protein [Nonomuraea sp. NEAU-A123]
Sequence ID: WP_214636464.1 Length: 210
Range 1: 20 to 169
Score:108 bits(269), Expect:6e-26,
Method:Compositional matrix adjust.,
Identities:63/151(42%), Positives:83/151(54%), Gaps:1/151(0%)
Query 32 ELTAVRSRVPSLYADPLAWLVADAAESALTQCATEVLAAREDVAVILVSAHCTLHTMDAM 91
+L A R S YADP AWLV A E+AL Q V ++ + VS H + HTM ++
Sbjct 20 DLVAEGRRTASFYADPAAWLVVSAVEAALAQAPEAVRREPAEIGALAVSDHGSRHTMRSV 79
Query 92 ARSLPAPRVSPLRFAGANPGSAGSLLCLLHGFKGSSLTLSMTPEDGLPAARTVARSWLRS 151
A S RVSPLRFAGANPGS LLC+ GF+G SL + M P+ P +A WL +
Sbjct 80 ADSAARGRVSPLRFAGANPGSLAGLLCITLGFRGPSLLVCMPPDRARPVVALIAAGWLAT 139
Query 152 GAASFVVLCTHRGHGEKGHSVRCAVLAPAAG 182

G +V + H +G H+V +L P+ G
Sbjct 140 GQCRYVAVAEHTMNGAH-HTVLGRILGPSPG 169
>polyketide synthase [Streptomyces sp. SID161]
Sequence ID: WP_161293594.1 Length: 188
Range 1: 25 to 163
Score:107 bits(267), Expect:9e-26,
Method:Compositional matrix adjust.,
Identities:63/140(45%), Positives:83/140(59%), Gaps:1/140(0%)
Query 41 PSLYADPLAWLVADAAESALTQCATEVLAAREDVAVILVSAHCTLHTMDAMARSLPAPRV 100
PS +ADP+AWLV A + AL A ++ AA + V VI +S C+ TM A+A S R+
Sbjct 25 PSFFADPVAWLVTAAVDRALRASADDLTAAPDRVGVITLSGTCSALTMTALADSARRGRI 84
Query 101 SPLRFAGANPGSAGSLLCLLHGFKGSSLTLSMTPEDGLPAARTVARSWLRSGAASFVVLC 160
SPLRFAGA+PG L C+ F+G +L L+M P GL A V +WL G AS V+L
Sbjct 85 SPLRFAGAHPGVLAGLTCIRRKFRGPTLALAMDPVHGLDPAAAVTAAWLGGGQASHVLLA 144
Query 161 THRGHGEKGHSVRCAVLAPA 180
H + H+ RCAV+ PA
Sbjct 145 VHLTR-DGIHTARCAVVRPA 163

>hypothetical protein [Streptomyces sp. 1114.5]
Sequence ID: WP_121174718.1 Length: 192
Range 1: 30 to 164
Score:107 bits(267), Expect:1e-25,
Method:Compositional matrix adjust.,
Identities:67/137(49%), Positives:83/137(60%), Gaps:3/137(2%)
Query 41   PSLYADPLAWLVADAAESALTQCATEVLAAREDVAVILVSAHCTLHTMDAMARSLPAPRV 100
PS +ADP+AWLVA A E AL   ++ A + V V+ +S C+ TM A+A S   V
Sbjct 30   PSFFADPVAWLVAAAVERALEASPDDLTAQPDHVGVVALSGDCSAITMAAVAASASRGMV 89
Query 101 SPLRFAGANPGSAGSLLCLLHGFKGSSLTLSMTPEDGLPAARTVARSWLRSGAASFVVLC 160
SPLRFAG+NPG   L C+   F+G +LTLSM PE GLPAA   A +WLR G A V++
Sbjct 90   SPLRFAGSNPGVLAGLPCIRWKFRGPTLTLSMPPEHGLPAAAVAASAWLRGGQARHVLIA 149
Query 161 THRGHGEKG-HSVRCAV 176
TH   E G H RCAV
Sbjct 150 THL--VEDGVHVARCAV 164
>polyketide synthase [Nonomuraea solani]
Sequence ID: WP_103963945.1 Length: 166
Range 1: 3 to 161

Score:106 bits(265), Expect:1e-25,
Method:Compositional matrix adjust.,
Identities:61/160(38%), Positives:88/160(55%), Gaps:1/160(0%)
Query 18 VDVAVVGHGDAFREELTAVRSRVPSLYADPLAWLVADAAESALTQCATEVLAAREDVAVI 77
+++ + G + ++LT+ R S YADP AWLV A E AL + +V E + +
Sbjct 3 IELVTLARGRSESDDLTSAGRRTTSFYADPAAWLVVAAVEDALARAPEKVRGEPERIGAL 62
Query 78 LVSAHCTLHTMDAMARSLPAPRVSPLRFAGANPGSAGSLLCLLHGFKGSSLTLSMTPEDG 137
VS H T HTM ++A + RVSPLRFAGA+PGS LLC+ GF+G SL +SM P+
Sbjct 63 AVSDHGTRHTMSSVAATAGRGRVSPLRFAGASPGSLAGLLCIALGFRGPSLLVSMRPDRA 122
Query 138 LPAARTVARSWLRSGAASFVVLCTHRGHGEKGHSVRCAVL 177
+A WL +G +V + H G+ H+V +L
Sbjct 123 HAVMELIASDWLATGQCRYVAVAEHTVTGDA-HTVSGRIL 161
>hypothetical protein [Streptacidiphilus sp. PB12-B1b]
Sequence ID: WP_225447214.1 Length: 592
Range 1: 404 to 565
Score:112 bits(281), Expect:2e-25,
Method:Compositional matrix adjust.,
Identities:68/162(42%), Positives:91/162(56%), Gaps:1/162(0%)
Query 22 VVGHGDAFREELTAVRSRVPSLYADPLAWLVADAAESALTQCATEVLAAREDVAVILVSA 81

V+GHG +L+ + PS YADPLAWLV A E AL C +LA R+ V V++++
Sbjct 404 VLGHGTRTVADLSTCSANRPSFYADPLAWLVNAAVEDALEDCRDTLLADRDQVGVLVLTG 463
Query 82 HCTLHTMDAMARSLPAPRVSPLRFAGANPGSAGSLLCLLHGFKGSSLTLSMTPEDGLPAA 141
L T +A ++ RVSPLRFAG NPG L C+ GF+G SL LS + + A
Sbjct 464 DRPLPTAGLIAATVARGRVSPLRFAGGNPGILAGLSCIERGFRGPSLVLSAAEDATVATA 523
Query 142 RTVARSWLRSGAASFVVLCTHRGHGE-KGHSVRCAVLAPAAG 182
+A +WL+ G A +VV HR E + +V C VL AAG
Sbjct 524 LALAEAWLQDGRARYVVCARHREEAEGEAQTVTCVVLRQAAG 565
>coronafacic acid synthetase component [Azospirillum sp. B510]
Sequence ID: BAI73721.1 Length: 149
Range 1: 6 to 145
Score:104 bits(259), Expect:5e-25,
Method:Compositional matrix adjust.,
Identities:61/144(42%), Positives:79/144(54%), Gaps:4/144(2%)
Query 39 RVPSLYADPLAWLVADAAESALTQCATEVLAAREDVAVILVSAHCTLHTMDAMARSLPAP 98
R+PSLYADPL+WLV +A A+T D+ I+VS CTLHTM +A ++P
Sbjct 6 RIPSLYADPLSWLVLEAVTDAVTAAGP---CLDRDLGHIVVSDVCTLHTMRGLAGAIPNG 62
Query 99 RVSPLRFAGANPGSAGSLLCLLHGFKGSSLTLSMTPEDGLPAARTVARSWLRSGAASFVV 158
R+SPLRF+GANPG L +G SL LSMTP G A V R+WLR A+ V+

Sbjct 63 RMSPLRFSGANPGQICGFAMQLLQLRGPSLVLSMTPSQGRHPAMIVLRNWLRERTAAAVI 122
Query 159 LCTHRGHGEKGHSVRCAVLAPAAG 182
L H ++ H V + G
Sbjct 123 LSLHEWDSDE-HRVTSTIFGTTDG 145
>polyketide synthase [Streptomyces sp. NRRL F-4489]
Sequence ID: WP_066984015.1 Length: 173
Range 1: 29 to 173
Score:105 bits(261), Expect:5e-25,
Method:Compositional matrix adjust.,
Identities:66/145(46%), Positives:78/145(53%), Gaps:1/145(0%)
Query 41 PSLYADPLAWLVADAAESALTQCATE-VLAAREDVAVILVSAHCTLHTMDAMARSLPAPR 99
PS YADP+AWLVA+ + AL CA V A ED ++++SA T TM +A S+P R
Sbjct 29 PSFYADPVAWLVAETVDRALADCAAAPVPDAAEDTGILVMSATGTRRTMRRIAGSVPRSR 88
Query 100 VSPLRFAGANPGSAGSLLCLLHGFKGSSLTLSMTPEDGLPAARTVARSWLRSGAASFVVL 159
VSPLRFAGANPG L L HG +G SL L+ P+D PAA TV W G A V L
Sbjct 89 VSPLRFAGANPGVLAGLPALRHGLRGPSLLLAAHPDDATPAAFTVIGGWFADGHARHVFL 148
Query 160 CTHRGHGEKGHSVRCAVLAPAAGRR 184
G C VL A R
Sbjct 149 VGLCGSAAGRELCSCLVLTAAGADR 173

>hypothetical protein [Streptomyces sp. MJM8645]
Sequence ID: WP_063351990.1 Length: 172
Range 1: 29 to 165
Score:104 bits(259), Expect:8e-25,
Method:Compositional matrix adjust.,
Identities:61/137(45%), Positives:76/137(55%), Gaps:0/137(0%)
Query 41 PSLYADPLAWLVADAAESALTQCATEVLAAREDVAVILVSAHCTLHTMDAMARSLPAPRV 100
PS YADP AWLVA+ + AL CA VL D ++++SA + TM +A S+P RV
Sbjct 29 PSFYADPAAWLVAETVDRALADCAEPVLDDAADTGILVMSATGSERTMRRIAASVPKSRV 88
Query 101 SPLRFAGANPGSAGSLLCLLHGFKGSSLTLSMTPEDGLPAARTVARSWLRSGAASFVVLC 160
SPLRFAGANPG L L H +G SL L+ P+ P A TV WL G A V+L
Sbjct 89 SPLRFAGANPGVLAGLPALRHELRGPSLLLAAHPDRATPVAFTVIGGWLADGHARHVLLV 148
Query 161 THRGHGEKGHSVRCAVL 177
+ +G RC VL
Sbjct 149 GLQPAAGEGQLCRCLVL 165
>coronafacic acid synthetase [Brenneria alni]

Sequence ID: WP_240630143.1 Length: 178
Range 1: 15 to 177
Score:103 bits(257), Expect:2e-24,
Method:Compositional matrix adjust.,
Identities:59/164(36%), Positives:91/164(55%), Gaps:2/164(1%)
Query 22 VVGHGDAFREELTAVR-SRVPSLYADPLAWLVADAAESALTQCATEVLAAREDVAVILVS 80
+VG G+A +L+ + ++ SLYADPL+WLV +A E A+ + +++A V I +S
Sbjct 15 IVGQGEANTADLSTYKPAQKASLYADPLSWLVLEAVEQAIAEHRDAIVSASASVGHIAIS 74
Query 81 AHCTLHTMDAMARSLPAPRVSPLRFAGANPGSAGSLLCLLHGFKGSSLTLSMTPEDGLPA 140
HCTL T+ + +L + +SPLRF+GA PG SL F G S+ SM P++ P
Sbjct 75 DHCTLKTIHDITATLSSGHLSPLRFSGACPGMIISLPAQFLHFNGPSIVWSMPPDNVQPY 134
Query 141 ARTVARSWLRSGAASFVVLCTHRGHGEKGHSVRCAVLAPAAGRR 184
A +AR WL G+ + V++ H GH + V+ G+R
Sbjct 135 AAALARIWLDEGSTTHVIITLHHM-DASGHHISSVVVTNNRGQR 177
>hypothetical protein [Couchioplanes caeruleus]
Sequence ID: WP_189205086.1 Length: 167
Range 1: 9 to 166
Score:103 bits(256), Expect:2e-24,
Method:Compositional matrix adjust.,

Identities:73/161(45%), Positives:94/161(58%), Gaps:3/161(1%)
Query 22 VVGHGDAFREELTAVRSRVPSLYADPLAWLVADAAESALTQCATEVLAAREDVAVILVSA 81
++G G+ + A R PS YADP AWL +A +AL + V A EDV V+ +SA
Sbjct 9 LLGRGEVALTDDIAARHSRPSFYADPAAWLAVEAVAAALGEAGAAVAQAGEDVGVLAISA 68
Query 82 HCTLHTMDAMARSLPAPRVSPLRFAGANPGSAGSLLCLLHGFKGSSLTLSMTPEDGLPAA 141
T TM A+ R+ A RVSPLRFAGANPGS L+C+ +GF+G SLTLSM PE P
Sbjct 69 TATTRTMRAIRRTAAAGRVSPLRFAGANPGSLAGLVCIEYGFRGPSLTLSMPPEQAWPVP 128
Query 142 RTVARSWLRSGAASFVVLCTHRGHGEKGHSVRCAVLAPAAG 182
+ + WL G +VV THRG G +G V AV+ +G
Sbjct 129 -VIRQDWL-DGPCRYVVTVTHRG-GPEGPEVAEAVVHGGSG 166
>hypothetical protein [Lentzea xinjiangensis]
Sequence ID: WP_089956931.1 Length: 190
Range 1: 23 to 160
Score:103 bits(257), Expect:3e-24,
Method:Compositional matrix adjust.,
Identities:61/138(44%), Positives:77/138(55%), Gaps:0/138(0%)
Query 41 PSLYADPLAWLVADAAESALTQCATEVLAAREDVAVILVSAHCTLHTMDAMARSLPAPRV 100
PS YAD +AWLVA+A + AL Q +V ++V V+++S T TM A+ RV
Sbjct 23 PSFYADSVAWLVAEAVDRALGQAGDQVGEVSDEVGVVVISEVATTGTMHAIGLGAARGRV 82

Query 101 SPLRFAGANPGSAGSLLCLLHGFKGSSLTLSMTPEDGLPAARTVARSWLRSGAASFVVLC 160
SPLRFAGANPG L C+ F+G SL LS +P L A V R WL S AA +VVL
Sbjct 83 SPLRFAGANPGVLAGLTCIRRRFRGPSLVLSTSPAAALGAGVAVVRGWLDSAAARYVVLA 142
Query 161 THRGHGEKGHSVRCAVLA 178
H H V CA++
Sbjct 143 AHFVTEAGEHVVNCAIVG 160
>hypothetical protein [Actinophytocola oryzae]
Sequence ID: WP_208298038.1 Length: 189
Range 1: 16 to 165
Score:103 bits(256), Expect:3e-24,
Method:Compositional matrix adjust.,
Identities:69/151(46%), Positives:88/151(58%), Gaps:6/151(3%)
Query 31 EELTAVRSRVPSLYADPLAWLVADAAESALTQCATEV--LAAREDVAVILVSAHCTLHTM 88
E T R+R SLYADP+AWLV A AL C + A +DV ++ +S CT T+
Sbjct 16 EPDTYSRNR-SSLYADPVAWLVDAAVRRALDSCGESLGHDGAADDVGILALSETCTARTI 74
Query 89 DAMARSLPAPRVSPLRFAGANPGSAGSLLCLLHGFKGSSLTLSMTPEDGLPAARTVARSW 148
++A RVSPLRFAGANPGS L CL F+G S+TL+M+ G+ A VA W
Sbjct 75 RSIAGMTARGRVSPLRFAGANPGSLAGLSCLTGRFRGPSVTLAMSVPAGVGPAVLVAAGW 134

Query 149 LRSGAASFVVLCTH--RGHGE-KGHSVRCAV 176
LR+G A+ VVL H R G+ + H RCAV
Sbjct 135 LRAGQATHVVLAVHDRRTDGDGQVHHARCAV 165
>hypothetical protein [Streptomyces triticirhizae]
Sequence ID: WP_233580373.1 Length: 591
Range 1: 403 to 562
Score:108 bits(271), Expect:3e-24,
Method:Compositional matrix adjust.,
Identities:65/161(40%), Positives:83/161(51%), Gaps:1/161(0%)
Query 20 VAVVGHGDAFREELTAVRSRVPSLYADPLAWLVADAAESALTQCATEVLAAREDVAVILV 79
+AV GH + + PS YADP+AWL+ A E AL C E LA ++VAV++
Sbjct 403 LAVAGHATLRVTDPASHTRDKPSFYADPVAWLIVSAVEEALAPCREEALAEPDEVAVLVT 462
Query 80 SAHCTLHTMDAMARSLPAPRVSPLRFAGANPGSAGSLLCLLHGFKGSSLTLSMTPEDGLP 139
L T +AR RVSPLRFAG+NPG L CL G +G SL L P D
Sbjct 463 HDGQALPTHRRIARDAARGRVSPLRFAGSNPGILAGLTCLTLGLRGPSLVLEADPADAGN 522
Query 140 AARTVARSWLRSGAASFVVLCTHRGHGEKGHSVRCAVLAPA 180
A + WLR+G A +V+ HR G+ H+ C VL P
Sbjct 523 AGTALVGDWLRTGRARYVLWAAHRADGDA-HTATCVVLRPG 562

>hypothetical protein [Pseudomonas frederiksbergensis]
Sequence ID: WP_148057932.1 Length: 174
Range 1: 17 to 136
Score:101 bits(252), Expect:1e-23,
Method:Compositional matrix adjust.,
Identities:51/123(41%), Positives:75/123(60%), Gaps:3/123(2%)
Query 41 PSLYADPLAWLVADAAESALTQCATEVLAAREDVAVILVSAHCTLHTMDAMARSLPAPRV 100
PSLYADPLAW V D + S L + E L E+V +I+VS +C+L TM A++ + ++
Sbjct 17 PSLYADPLAWAVVDFSASLLNKVPEESL---ENVGIIVVSDYCSLATMRALSATSKDGKI 73
Query 101 SPLRFAGANPGSAGSLLCLLHGFKGSSLTLSMTPEDGLPAARTVARSWLRSGAASFVVLC 160
SPL+FAGANPG L + + +G S+TL+M P + A +VAR W + V++
Sbjct 74 SPLKFAGANPGVVTGLTAIQYKLRGPSVTLTMNPANASKAVTSVARYWFKHSGVHSVLVI 133
Query 161 THR 163
TH+
Sbjct 134 THK 136
>polyketide synthase [Streptomyces noursei]
Sequence ID: WP_102922765.1 Length: 172
Range 1: 10 to 147

Score:100 bits(250), Expect:2e-23,
Method:Compositional matrix adjust.,
Identities:60/138(43%), Positives:77/138(55%), Gaps:0/138(0%)
Query 22 VVGHGDAFREELTAVRSRVPSLYADPLAWLVADAAESALTQCATEVLAAREDVAVILVSA 81
V+G E+ A PS YADP AWLVA+ + AL CA V A +D A++++SA
Sbjct 10 VLGSATVVAEDPAAYTRNKPSFYADPAAWLVAETVDRALAGCAELVGDATDDTAILVMSA 69
Query 82 HCTLHTMDAMARSLPAPRVSPLRFAGANPGSAGSLLCLLHGFKGSSLTLSMTPEDGLPAA 141
+ T+ +A S+P RVSPLRFAGANPG L L H +G SL L+ P+ P A
Sbjct 70 TGSERTIRRIADSVPRSRVSPLRFAGANPGVLAGLPALRHRLRGPSLLLAAHPDTATPVA 129
Query 142 RTVARSWLRSGAASFVVL 159
TV WL G A V+L
Sbjct 130 FTVIDRWLADGHARHVIL 147
>hypothetical protein [Dactylosporangium vinaceum]
Sequence ID: WP_223094296.1 Length: 168
Range 1: 5 to 167
Score:100 bits(248), Expect:3e-23,
Method:Compositional matrix adjust.,
Identities:73/165(44%), Positives:85/165(51%), Gaps:17/165(10%)

Query 28 AFREELTAVRSRVPSLYADPLAWLVADAAESAL-------------TQCATEVLAARED- 73
A+ E TA PS Y DP WLV A AL T A A D
Sbjct 5 AYGEADTADARNAPSFYVDPAGWLVNTAVSGALQTSGALQTSGALRTSGALRTTAIEPDE 64
Query 74 VAVILVSAHCTLHTMDAMARSLPAPRVSPLRFAGANPGSAGSLLCLLHGFKGSSLTLSMT 133
VAVI+VS C+ TM ++A S VSPLRFAGANPG L C+ +G SLTL+M
Sbjct 65 VAVIVVSDICSATTMASIAASAERGVVSPLRFAGANPGVLAGLPCIRGKLRGPSLTLTMP 124
Query 134 PEDGLPAARTVARSWLRSGAASFVVLCTHRGHGEKGHSV-RCAVL 177
P++GLP A VA WLR G A VVL T+ E G V RCAV+
Sbjct 125 PDEGLPVAAVVAAGWLRDGHARHVVLATY--AIEAGRPVARCAVV 167
>hypothetical protein, partial [Pseudomonas syringae group genomosp. 3]
Sequence ID: WP_032645024.1 Length: 119
Range 1: 1 to 110
Score:96.7 bits(239), Expect:2e-22,
Method:Compositional matrix adjust.,
Identities:58/111(52%), Positives:71/111(63%), Gaps:1/111(0%)
Query 67 VLAAREDVAVILVSAHCTLHTMDAMARSLPAPRVSPLRFAGANPGSAGSLLCLLHGFKGS 126
VLAA + V I+VS CTLHTM +AR L R+SPLRF+GA PG SL L F G
Sbjct 1 VLAAGQTVGHIVVSDVCTLHTMQHIARDLGRNRLSPLRFSGACPGLVCSLAGQLLHFSGP 60
Query 127 SLTLSMTPEDGLPAARTVARSWLRSGAASFVVLCTHRGHGEKGHSVRCAVL 177

S+ LSM P+DGL A +A+ WL SGAAS V++ TH   G + H VRC +L
Sbjct 61   SMVLSMPPQDGLVPAALLAQDWLDSGAASHVLVSTHDTDGTQ-HRVRCTLL 110
>hypothetical protein [Dickeya poaceiphila]
Sequence ID: WP_042868955.1 Length: 177
Range 1: 14 to 176
Score:97.1 bits(240), Expect:7e-22,
Method:Compositional matrix adjust.,
Identities:59/164(36%), Positives:88/164(53%), Gaps:2/164(1%)
Query 22   VVGHGDAFREELTAVRS-RVPSLYADPLAWLVADAAESALTQCATEVLAAREDVAVILVS 80
+ G G A   +L+ +S + SLYADPL+WL+ + E A+   + +A   V I +S
Sbjct 14   IAGQGMASTADLSTYKSAQKASLYADPLSWLILETVEQAIAGHRDAITSACASVGHIAIS 73
Query 81   AHCTLHTMDAMARSLPAPRVSPLRFAGANPGSAGSLLCLLHGFKGSSLTLSMTPEDGLPA 140
CTL T+ +A +L + +SPLRF+GA PG+ SL   F G S+ SM P++
Sbjct 74   DQCTLKTIHDIAATLSSGHLSPLRFSGACPGTVISLPSQFRRFSGPSMVWSMPPDNTQHY 133
Query 141 ARTVARSWLRSGAASFVVLCTHRGHGEKGHSVRCAVLAPAAGRR 184
A +AR WL GAA+ V++ TH   GH +   V+ + G R
Sbjct 134 AAVLARVWLDEGAATHVIITTHHV-DASGHHIHSIVVTHSRGER 176

>MULTISPECIES: hypothetical protein [Brenneria]
Sequence ID: WP_009114554.1 Length: 177
Range 1: 12 to 176
Score:96.7 bits(239), Expect:8e-22,
Method:Compositional matrix adjust.,
Identities:57/166(34%), Positives:90/166(54%), Gaps:2/166(1%)
Query 20   VAVVGHGDAFREELTAVR-SRVPSLYADPLAWLVADAAESALTQCATEVLAAREDVAVIL 78
+ +VG G A   +L+ + ++ SLYADPL+WLV +A E A+ +   + +A   V I
Sbjct 12   LKIVGQGMASAADLSTYKPAQKASLYADPLSWLVLEAVEQAIIEHRDVITSACSSVGHIA 71
Query 79   VSAHCTLHTMDAMARSLPAPRVSPLRFAGANPGSAGSLLCLLHGFKGSSLTLSMTPEDGL 138
+S HCTL T+ ++ +LP+ +SPLRF+GA PG   L   F G S+ SM ++
Sbjct 72   ISDHCTLKTIHDISATLPSGHLSPLRFSGACPGMVIGLPSQFLRFSGPSIVWSMPSDNMQ 131
Query 139 PAARTVARSWLRSGAASFVVLCTHRGHGEKGHSVRCAVLAPAAGRR 184
A +A+ WL G+A+ V++ H   E GH +   V+ + G
Sbjct 132 IYATALAQIWLDEGSATHVIITDHHV-DESGHHIHSVVVTQSGGEH 176
>polyketide synthase [Streptomyces longispororuber]
Sequence ID: WP_190135286.1 Length: 208
Range 1: 20 to 168

Score:97.4 bits(241), Expect:1e-21,
Method:Compositional matrix adjust.,
Identities:63/151(42%), Positives:77/151(50%), Gaps:2/151(1%)
Query 27   DAFREELTAVRSRVPSLYADPLAWLVADAAESALTQCATEVLAAREDVAVILVSAHCTLH 86
D   E+L A   PS YADP+AWL   A   AL +C   DV V+++SA CT
Sbjct 20   DVVTEDLGAGADNRPSFYADPVAWLTTAAVTRALARC-DRGTGPCPDVGVVVMSATCTAP 78
Query 87   TMDAMARSLPAPRVSPLRFAGANPGSAGSLLCLLHGFKGSSLTLSMTPEDGLPAARTVAR 146
TM +AR+   RVSPL+FAGANPG   L C+   F G SL LS P   + A VA
Sbjct 79   TMAVIARTASRSRVSPLKFAGANPGILAGLPCIRGQFHGPSLVLSTEPAVSVDTALCVAV 138
Query 147 SWLRSGAASFVVLCTHRGHGEKGHSVRCAVL 177
WL +   + +   H   G GH VRC VL
Sbjct 139 HWLWTRQSRYAACVAHTRPG-GGHRVRCVVL 168
>hypothetical protein [Streptomyces sp. CB03238]
Sequence ID: WP_084903002.1 Length: 210
Range 1: 23 to 168
Score:96.7 bits(239), Expect:2e-21,
Method:Compositional matrix adjust.,
Identities:57/147(39%), Positives:76/147(51%), Gaps:1/147(0%)
Query 31   EELTAVRSRVPSLYADPLAWLVADAAESALTQCATEVLAAREDVAVILVSAHCTLHTMDA 90

E+ A + PS YADP+AWL AL +L ++V VI +S CT TM+
Sbjct 23 EDPRAGSANRPSFYADPVAWLTTSTVARALRAEGAGILHTPDEVGVIAMSETCTRPTMET 82
Query 91 MARSLPAPRVSPLRFAGANPGSAGSLLCLLHGFKGSSLTLSMTPEDGLPAARTVARSWLR 150
+A + RVSPL+FAGANPG L C+ F+G SL +M P+ A +VA WL
Sbjct 83 IAGAAGRSRVSPLKFAGANPGLLAGLPCIQWTFRGPSLVFAMAPDTAADTALSVAEHWLS 142
Query 151 SGAASFVVLCTHRGHGEKGHSVRCAVL 177
SG A + V C + H RC +L
Sbjct 143 SGQARYAV-CVAHAVRDGAHHTRCVIL 168
>hypothetical protein [Streptomyces sp. SLBN-115]
Sequence ID: WP_142264107.1 Length: 182
Range 1: 27 to 171
Score:94.7 bits(234), Expect:5e-21,
Method:Compositional matrix adjust.,
Identities:56/145(39%), Positives:78/145(53%), Gaps:2/145(1%)
Query 36 VRSRVPSLYADPLAWLVADAAESALTQCATEVLAAREDVAVILVSAHCTLHTMDAMARSL 95
V RVP +YADP+AWL+A+ +AL C + E+V VI VS T HT+ A+A S+
Sbjct 27 VGRRVPGMYADPVAWLMAETVAAALENCGDAAPSRPEEVGVIGVSERATRHTLRALAESV 86
Query 96 PAPRVSPLRFAGANPGSAGSLLCLLHGFKGSSLTLSMTPEDGLPAARTVARSWLRSG--A 153
P R+SP+RFAGA GS ++C GF G L M +P R + WL +

Sbjct 87   PRGRISPMRFAGAGAGSLVGVVCSAFGFHGPVSMLPMPLTPAVPLTRALCGDWLLGDPPS 146
Query 154 ASFVVLCTHRGHGEKGHSVRCAVLA 178
A+ V++ TH   E H   C V++
Sbjct 147 AAHVLVVTHDVDDEGKHRAHCLVVS 171
>hypothetical protein [Actinomadura roseirufa]
Sequence ID: WP_131740835.1 Length: 179
Range 1: 12 to 152
Score:92.8 bits(229), Expect:3e-20,
Method:Compositional matrix adjust.,
Identities:56/141(40%), Positives:73/141(51%), Gaps:0/141(0%)
Query 22   VVGHGDAFREELTAVRSRVPSLYADPLAWLVADAAESALTQCATEVLAAREDVAVILVSA 81
V G G A   +L +   S YADP+AWLV DA E AL +   A   V+ VS
Sbjct 12   VAGDGLARTADLGSAGRTRASFYADPVAWLVVDALELALERAGPHAREALARTGVLAVSE 71
Query 82   HCTLHTMDAMARSLPAPRVSPLRFAGANPGSAGSLLCLLHGFKGSSLTLSMTPEDGLPAA 141
+ T HTM ++ R   RVSPLRFAGA+PGS   L C++ GF+G +L L M P   AA
Sbjct 72   YATRHTMGSIGRQAARGRVSPLRFAGASPGSLAGLACVVLGFRGPTLLLGMPPATARAAA 131
Query 142 RTVARSWLRSGAASFVVLCTH 162
+   W +G   +V + H
Sbjct 132 LAIVSHWTATGQCRYVAVAEH 152

>hypothetical protein [Streptomyces sp. ST2-7A]
Sequence ID: WP_233865706.1 Length: 588
Range 1: 403 to 549
Score:97.8 bits(242), Expect:3e-20,
Method:Compositional matrix adjust.,
Identities:63/147(43%), Positives:81/147(55%), Gaps:0/147(0%)
Query 20   VAVVGHGDAFREELTAVRSRVPSLYADPLAWLVADAAESALTQCATEVLAAREDVAVILV 79
+AV GH   + +   PS YADP+AWL+ AAE AL C EVLA ++VAV+L
Sbjct 403 LAVAGHATLSVADPASCTRNKPSFYADPIAWLIVSAAEEALVPCREEVLADPDEVAVLLT 462
Query 80   SAHCTLHTMDAMARSLPAPRVSPLRFAGANPGSAGSLLCLLHGFKGSSLTLSMTPEDGLP 139
+   L T   +AR   RVSPLRFAG+NPG   L CL G +G SL L   P+D
Sbjct 463 NDGRALPTHLRIARDAARGRVSPLRFAGSNPGILAGLTCLTLGLRGPSLVLEADPDDAAD 522
Query 140 AARTVARSWLRSGAASFVVLCTHRGHG 166
A +A WL +G A +V+   +R HG
Sbjct 523 VATALAGHWLHAGRARYVLWAAYRQHG 549
>polyketide synthase [Actinomycetia bacterium]

Sequence ID: MBI3688123.1 Length: 203
Range 1: 18 to 167
Score:93.2 bits(230), Expect:4e-20,
Method:Compositional matrix adjust.,
Identities:65/151(43%), Positives:85/151(56%), Gaps:4/151(2%)
Query 30 REELT---AVRSRVPSLYADPLAWLVADAAESALTQCATEVLAAREDVAVILVSAHCTLH 86
R E+T A+ P+ YADP AWLVA A + AL + +DVA+++ + T
Sbjct 18 RREVTDPGALSRNAPAFYADPAAWLVAAALDGALESGQVTLPDPADDVAMLVAGSVGTTR 77
Query 87 TMDAMARSLPAPRVSPLRFAGANPGSAGSLLCLLHGFKGSSLTLSMTPEDGLPAARTVAR 146
T+DA+ + +SPLRFAG NP L CL +G SL L+M P D +P A TVA
Sbjct 78 TIDALRQGAARGVISPLRFAGGNPAVLAGLSCLTRRLRGPSLLLAMDPWDAVPVAATVAV 137
Query 147 SWLRSGAASFVVLCTHRGHGEKGHSVRCAVL 177
SWL +G AS VV+ TH G + SVRC VL
Sbjct 138 SWLVAGHASHVVVATHHVIGGR-ESVRCVVL 167
>hypothetical protein [Streptomyces bobili]
Sequence ID: WP_086771546.1 Length: 182
Range 1: 13 to 171
Score:92.0 bits(227), Expect:7e-20,
Method:Compositional matrix adjust.,

Identities:55/159(35%), Positives:82/159(51%), Gaps:2/159(1%)
Query 22 VVGHGDAFREELTAVRSRVPSLYADPLAWLVADAAESALTQCATEVLAAREDVAVILVSA 81
V G ++ V RV +YADP+AWL+ +A +AL C E+V V+ V+
Sbjct 13 VTGSATVETDDPGTVPRRVSGVYADPVAWLMTEAVAAALKACGDAAPENPEEVGVVAVAE 72
Query 82 HCTLHTMDAMARSLPAPRVSPLRFAGANPGSAGSLLCLLHGFKGSSLTLSMTPEDGLPAA 141
T+ T+ A+A ++P R+SP+RFAGA PGS ++C GF G + L M +P A
Sbjct 73 RATVRTLRALADAVPHGRISPMRFAGAGPGSVVGVVCSAFGFHGPASMLPMPLAPAVPLA 132
Query 142 RTVARSWLRSG--AASFVVLCTHRGHGEKGHSVRCAVLA 178
R + WL A+ V++ TH E H C V++
Sbjct 133 RALCGDWLLGDPPGAAHVLVVTHGTTDEGKHQAHCLVVS 171
>hypothetical protein [Nonomuraea guangzhouensis]
Sequence ID: WP_219528372.1 Length: 235
Range 1: 20 to 169
Score:92.0 bits(227), Expect:2e-19,
Method:Compositional matrix adjust.,
Identities:61/151(40%), Positives:81/151(53%), Gaps:1/151(0%)
Query 32 ELTAVRSRVPSLYADPLAWLVADAAESALTQCATEVLAAREDVAVILVSAHCTLHTMDAM 91
+L A R S YADP AWLV A E+AL + V ++ + VS H T HTM ++
Sbjct 20 DLVAEGRRTASFYADPAAWLVVSAVEAALAEAPEAVRWETAEIGALAVSDHGTRHTMRSV 79

Query 92   ARSLPAPRVSPLRFAGANPGSAGSLLCLLHGFKGSSLTLSMTPEDGLPAARTVARSWLRS 151
A S   RVSPLRFAGANPGS   LLC+ GF+G SL + M P+   +A WL +
Sbjct 80   ADSAARGRVSPLRFAGANPGSLAGLLCITLGFRGPSLLVCMPPDRAARVVALIAAGWLAT 139
Query 152 GAASFVVLCTHRGHGEKGHSVRCAVLAPAAG 182
G   +V + H +G   H+   +L P+ G
Sbjct 140 GECRYVAVAEHTVNGTH-HTAVGRILGPSPG 169
>hypothetical protein [Allokutzneria albata]
Sequence ID: WP_052408053.1 Length: 156
Range 1: 15 to 149
Score:89.7 bits(221), Expect:2e-19,
Method:Compositional matrix adjust.,
Identities:52/139(37%), Positives:75/139(53%), Gaps:4/139(2%)
Query 39   RVPSLYADPLAWLVADAAESALTQCATEVLAAREDVAVILVSAHCTLHTMDAMARSLPAP 98
R+ +YADP+AWL+ DA   L   T +   V V++VS H T HT ++A ++
Sbjct 15   RISGIYADPVAWLITDAVAGVLN---TRAVLDPTSVGVLVVSEHSTEHTQRSVAEAVARG 71
Query 99   RVSPLRFAGANPGSAGSLLCLLHGFKGSSLTLSMTPEDGLPAARTVARSWLRSGAASFVV 158
R+SP+RFA A PGS   ++C   GF+G +L LS+ E   P   +   WL   +A VV
Sbjct 72   RISPMRFAAAGPGSLVGVVCAAFGFQGPTLLLSVPVEQARPVVDALLADWLHD-SAGHVV 130

Query 159 LCTHRGHGEKGHSVRCAVL 177
L H + HSV C+V+
Sbjct 131 LVMHEVAEDGRHSVTCSVV 149
>coronafacic acid synthetase component [Nguyenibacter vanlangensis]
Sequence ID: WP_218064164.1 Length: 173
Range 1: 15 to 151
Score:89.4 bits(220), Expect:5e-19,
Method:Compositional matrix adjust.,
Identities:46/141(33%), Positives:76/141(53%), Gaps:4/141(2%)
Query 37 RSRVPSLYADPLAWLVADAAESALTQCATEVLAAREDVAVILVSAHCTLHTMDAMARSLP 96
R+R PS+YADP+AW V D A + + + R +V ++ +S C+L TM ++ S
Sbjct 15 RNR-PSMYADPVAWAVLDFASTIIEELGD---VERANVGLLAISDVCSLSTMRLLSDSSK 70
Query 97 APRVSPLRFAGANPGSAGSLLCLLHGFKGSSLTLSMTPEDGLPAARTVARSWLRSGAASF 156
++SPLRFAGANPG L + + +G SL L+M+PE + V + W+ +
Sbjct 71 NGKISPLRFAGANPGIIAGLTAIEYKLRGPSLVLTMSPERAVAVVLPVLKYWIEQNGVAQ 130
Query 157 VVLCTHRGHGEKGHSVRCAVL 177
V+L H + + +R ++
Sbjct 131 VILVAHEKNRDLNDVLRGVII 151

>hypothetical protein BJF79_25760 [Actinomadura sp. CNU-125]
Sequence ID: OLT10694.1 Length: 632
Range 1: 446 to 614
Score:92.4 bits(228), Expect:2e-18,
Method:Compositional matrix adjust.,
Identities:74/170(44%), Positives:94/170(55%), Gaps:3/170(1%)
Query 14 PPDTVDVAVVGHGDAFREELTAVRSRVPSLYADPLAWLVADAAESALT--QCATEVLAAR 71
P T AVVGHG ++ A + PS YADP AWLVA A +A T +C T+VLAAR
Sbjct 446 PTGTGTAAVVGHGAVTVDDPAACSADRPSFYADPAAWLVAAAVAAASTTAECGTDVLAAR 505
Query 72 EDVAVILVSAHCTLHTMDAMARSLPAPRVSPLRFAGANPGSAGSLLCLLHGFKGSSLTLS 131
+D VI+++ T A+A R+SPLRFAGANPG L C+ G +G SL L+
Sbjct 506 DDAGVIVLTGPQPPPTCTALAAQAARGRISPLRFAGANPGILAGLACIRWGLRGPSLVLA 565
Query 132 MTPEDGLPAARTVARSWLRSGAASFVVLCTHRGHGEKGHSVRCAVLAPAA 181
+ + A TVA SWLRS A V+ H + H+ CAVL AA
Sbjct 566 AADDGTVGTALTVAGSWLRSRRARHVICVRHLARPGR-HTALCAVLRAAA 614
>hypothetical protein [Streptomyces actinomycinicus]
Sequence ID: WP_201843267.1 Length: 182
Range 1: 27 to 178

Score:87.4 bits(215), Expect:4e-18,
Method:Compositional matrix adjust.,
Identities:61/152(40%), Positives:83/152(54%), Gaps:3/152(1%)
Query 36 VRSRVPSLYADPLAWLVADAAESALTQCATEVLAAREDVAVILVSAHCTLHTMDAMARSL 95
VR RV +YADP+AWL+A+A +AL +C T E+V VI VS H T T+ A+A ++
Sbjct 27 VRRRVSGVYADPVAWLMAEAVAAALQECGTAAPDRPEEVGVIGVSEHATRITLRALADAV 86
Query 96 PAPRVSPLRFAGANPGSAGSLLCLLHGFKGSSLTLSMTPEDGLPAARTVARSWLRSG--A 153
P R+SP+RFAGA PG+ ++C GF G L M P AR + WL
Sbjct 87 PQGRLSPMRFAGAGPGALIGMVCSAFGFHGPVSMLPMPLAPAAPLARALCGDWLLGDPPG 146
Query 154 ASFVVLCTHRGHGEKGHSVRCAVLA-PAAGRR 184
A+ V++ TH E H C V++ P RR
Sbjct 147 AAHVLVVTHATTDEGKHQAHCLVVSGPTPTRR 178
>hypothetical protein [Acinetobacter sp. A47]
Sequence ID: WP_052209145.1 Length: 170
Range 1: 13 to 134
Score:85.9 bits(211), Expect:1e-17,
Method:Compositional matrix adjust.,
Identities:43/125(34%), Positives:67/125(53%), Gaps:3/125(2%)

Query 38 SRVPSLYADPLAWLVADAAESALTQCATEVLAAREDVAVILVSAHCTLHTMDAMARSLPA 97
S PSLYADPL W V D A V ++ +I+VS +CT TM + +
Sbjct 13 SNRPSLYADPLGWAVVDFLS---VNFAGIVEKNNDNTGIIVVSDYCTQLTMKNLYPGIKK 69
Query 98 PRVSPLRFAGANPGSAGSLLCLLHGFKGSSLTLSMTPEDGLPAARTVARSWLRSGAASFV 157
+SPL+FAGANPG L +++G +G+S+TLSM+P+ A ++ + ++ S +
Sbjct 70 GYISPLKFAGANPGVMAGLPAIIYGLRGASMTLSMSPKFSYKAIYSLVKFLFKNDQVSSI 129
Query 158 VLCTH 162
+ H
Sbjct 130 FIILH 134
>coronafacic acid synthetase component [Nguyenibacter vanlangensis]
Sequence ID: NVN10070.1 Length: 154
Range 1: 1 to 132
Score:85.1 bits(209), Expect:2e-17,
Method:Compositional matrix adjust.,
Identities:42/135(31%), Positives:71/135(52%), Gaps:3/135(2%)
Query 43 LYADPLAWLVADAAESALTQCATEVLAAREDVAVILVSAHCTLHTMDAMARSLPAPRVSP 102
+YADP+AW V D A + + + R +V ++ +S C+L TM ++ S ++SP
Sbjct 1 MYADPVAWAVLDFASTIIEELGD---VERANVGLLAISDVCSLSTMRLLSDSSKNGKISP 57
Query 103 LRFAGANPGSAGSLLCLLHGFKGSSLTLSMTPEDGLPAARTVARSWLRSGAASFVVLCTH 162

LRFAGANPG L + + +G SL L+M+PE + V + W+ + V+L H
Sbjct 58 LRFAGANPGIIAGLTAIEYKLRGPSLVLTMSPERAVAVVLPVLKYWIEQNGVAQVILVAH 117
Query 163 RGHGEKGHSVRCAVL 177
+ + +R ++
Sbjct 118 EKNRDLNDVLRGVII 132
>coronafacic acid synthetase [Streptomyces aurantiogriseus]
Sequence ID: WP_189938222.1 Length: 182
Range 1: 27 to 171
Score:85.1 bits(209), Expect:3e-17,
Method:Compositional matrix adjust.,
Identities:55/145(38%), Positives:78/145(53%), Gaps:2/145(1%)
Query 36 VRSRVPSLYADPLAWLVADAAESALTQCATEVLAAREDVAVILVSAHCTLHTMDAMARSL 95
V R+ +YADP+AWL+A+A +AL +C E+V V+ VS H T HT+ A+A ++
Sbjct 27 VGRRIAGVYADPVAWLMAEAVAAALEECGEAAPERPEEVGVVGVSEHATRHTLRALADAV 86
Query 96 PAPRVSPLRFAGANPGSAGSLLCLLHGFKGSSLTLSMTPEDGLPAARTVARSWLRSG--A 153
P R+SP+RFAGA PGS ++C GF G L M P R + WL
Sbjct 87 PRGRLSPMRFAGAGPGSLVGVVCSAFGFHGPVSMLPMPLAPAAPLTRALCGDWLLGDPPG 146
Query 154 ASFVVLCTHRGHGEKGHSVRCAVLA 178
A+ V++ TH E H C V++

Sbjct 147 AAHVLVVTHATTDEGKHQAHCLVVS 171
>polyketide synthase [Streptomyces sp. UNOC14_S4]
Sequence ID: MCC3772573.1 Length: 109
Range 1: 1 to 109
Score:80.5 bits(197), Expect:3e-16,
Method:Compositional matrix adjust.,
Identities:49/110(45%), Positives:61/110(55%), Gaps:1/110(0%)
Query 75 AVILVSAHCTLHTMDAMARSLPAPRVSPLRFAGANPGSAGSLLCLLHGFKGSSLTLSMTP 134
++++SA + TM +ARS+P RVSPLRFAGANPG L L HG +G SL L+M P
Sbjct 1 GIVVMSATGSEQTMRVIARSVPRSRVSPLRFAGANPGVLAGLSALRHGLRGPSLLLAMHP 60
Query 135 EDGLPAARTVARSWLRSGAASFVVLCTHRGHGEKGHSVRCAVLAPAAGRR 184
+ P A TV WL G A V+L + GH RC VL A R
Sbjct 61 DAATPVALTVIDGWLADGQARHVLLAGLEPTSD-GHLCRCQVLTGAGADR 109
>hypothetical protein [Burkholderia ubonensis]
Sequence ID: WP_162296926.1 Length: 175
Range 1: 9 to 150

Score:80.1 bits(196), Expect:2e-15,
Method:Compositional matrix adjust.,
Identities:46/145(32%), Positives:74/145(51%), Gaps:4/145(2%)
Query 34 TAVRSR-VPSLYADPLAWLVADAAESALTQCATEVLAAREDVAVILVSAHCTLHTMDAMA 92
+ +RSR PS YA P+AW D +S + + V +I+VS C+L T+ +A
Sbjct 9 SEIRSRNKPSFYAAPVAWATGDFVQSLIDESDAIV---PNHTGMIVVSDECSLDTIRELA 65
Query 93 RSLPAPRVSPLRFAGANPGSAGSLLCLLHGFKGSSLTLSMTPEDGLPAARTVARSWLRSG 152
+ +SPLRFAGA+P + L G +G +LTL+M+PE + R W+
Sbjct 66 GAAAQGGISPLRFAGASPSIVVGVPALQQGIRGPTLTLTMSPEHAADPIVAMIRYWIERN 125
Query 153 AASFVVLCTHRGHGEKGHSVRCAVL 177
V++ HR HG + H ++ ++
Sbjct 126 GVDAVIVVAHRRHGAQAHLLKGLIV 150
>polyketide synthase [Streptomyces inusitatus]
Sequence ID: WP_190125360.1 Length: 184
Range 1: 37 to 179
Score:80.1 bits(196), Expect:3e-15,
Method:Compositional matrix adjust.,
Identities:54/143(38%), Positives:67/143(46%), Gaps:4/143(2%)
Query 39 RVPSLYADPLAWLVADAAESAL----TQCATEVLAAREDVAVILVSAHCTLHTMDAMARS 94

R S YADP+A LV+D   L   V+ VS   TLHTM +A
Sbjct 37   RTVSSYADPVALLVSDTVAEVLALLPETDTEPGTGTGTSTGVLTVSETGTLHTMRTLAAG 96
Query 95   LPAPRVSPLRFAGANPGSAGSLLCLLHGFKGSSLTLSMTPEDGLPAARTVARSWLRSGAA 154
L   R+SPLRFAGA PGS   L C++HG +G SL L+M P   P   +   WL +G
Sbjct 97   LGRGRISPLRFAGAGPGSLAGLACIVHGLRGPSLVLTMPPTAAEPLLVPLVHGWLTTGGC 156
Query 155 SFVVLCTHRGHGEKGHSVRCAVL 177
VV+ H   H VR V+
Sbjct 157 RQVVVNEHLTDAAGTHFVRSRVV 179
>hypothetical protein [Lonsdalea britannica]
Sequence ID: WP_217809648.1 Length: 118
Range 1: 10 to 109
Score:73.9 bits(180), Expect:1e-13,
Method:Compositional matrix adjust.,
Identities:41/101(41%), Positives:57/101(56%), Gaps:1/101(0%)
Query 77   ILVSAHCTLHTMDAMARSLPAPRVSPLRFAGANPGSAGSLLCLLHGFKGSSLTLSMTPED 136
I +S CT HT+ +   + + R+SPLRF+GA PG   +L   GF G S+ LSM E
Sbjct 10   IAISDQCTAHTLREIGAVIASGRISPLRFSGACPGLVCALPGQFLGFNGPSMVLSMPAEQ 69
Query 137 GLPAARTVARSWLRSGAASFVVLCTHRGHGEKGHSVRCAVL 177
GLPAA +A++WL   AAS V++ H   GH+V   +

Sbjct 70   GLPAAAAIAKTWLSEHAASHVIVTCHEA-DAAGHTVTSVIF 109
>polyketide synthase [Thermomonospora umbrina]
Sequence ID: WP_116025564.1 Length: 155
Range 1: 19 to 148
Score:74.7 bits(182), Expect:1e-13,
Method:Compositional matrix adjust.,
Identities:47/133(35%), Positives:63/133(47%), Gaps:3/133(2%)
Query 42   SLYADPLAWLVADAAESALTQCATEVLAAREDVAVILVSAHCTLHTMDAMARSLPAPRVS 101
S YADP+AW++ A   L   A +   + A+I+ S   TL T   +A   R+
Sbjct 19   SFYADPIAWMILAAVTQTLADIAPD--PDPDTTALIVTSTATTLPTCQTIAVEARRGRIR 76
Query 102 PLRFAGANPGSAGSLLCLLHGFKGSSLTLSMTPEDGLPAARTVARSWLRSGAASFVVLCT 161
PLRFAGANPG   L C+ G +G S+ L   P   A+ +A W   G A V++
Sbjct 77   PLRFAGANPGILAGLSCIRLGLRGPSMVLLGDPTGTARTAQALASQWQAQGQARLVMVVD 136
Query 162 HRGHGEKGHSVRC 174
HR   GH V C
Sbjct 137 HR-QTSAGHRVTC 148

>hypothetical protein [Planctomycetes bacterium]
Sequence ID: MBS0266747.1 Length: 196
Range 1: 17 to 176
Score:75.1 bits(183), Expect:2e-13,
Method:Compositional matrix adjust.,
Identities:51/160(32%), Positives:73/160(45%), Gaps:1/160(0%)
Query 24 GHGDAFREELTAVRSRVPSLYADPLAWLVADAAESALTQCATEVLAAREDVAVILVSAHC 83
G D + +L + R YADP AW A +A AL + R+ V VI+V
Sbjct 17 GQADQHQPDLFDAKLRNVVRYADPAAWTCAVSAVHALASVPDRLSGDRDRVGVIVVCDDG 76
Query 84 TLHTMDAMARSLPAPRVSPLRFAGANPGSAGSLLCLLHGFKGSSLTLSMTPEDGLPAART 143
M A+ + SPL+F NPGS + C+L GF+G +L M P G+PA
Sbjct 77 PQEAMQALDEAAVKGFSSPLKFPAGNPGSLVGVTCILLGFRGPTLNFIMPPASGVPAGLV 136
Query 144 VARSWLRSGAASFVVLC-THRGHGEKGHSVRCAVLAPAAG 182
+A WL+ S V+L + R G ++ + AP G
Sbjct 137 MAAGWLQRNVCSHVLLTASSRAAGATPNARTLLLTAPQDG 176
>hypothetical protein [Agrobacterium tumefaciens]
Sequence ID: WP_143238584.1 Length: 167
Range 1: 2 to 146
Score:74.3 bits(181), Expect:3e-13,

Method:Compositional matrix adjust.,
Identities:49/147(33%), Positives:79/147(53%), Gaps:4/147(2%)
Query 33   LTAVRSR-VPSLYADPLAWLVADAAESALTQCATEVLAA-REDVAVILVSAHCTLHTMDA 90
LT + S+ PS++ADPLAW VAD   L + TE+ ++ R   +I+VS C+L T+ A
Sbjct 2   LTVLESKNAPSIFADPLAWAVADFVGGLLGE--TELASSDRIQTGLIVVSDLCSLSTIRA 59
Query 91   MARSLPAPRVSPLRFAGANPGSAGSLLCLLHGFKGSSLTLSMTPEDGLPAARTVARSWLR 150
++   +SPL+FAGA+P   L L   +G ++T +M P   A   + R W+
Sbjct 60   LSAMAKQDFLSPLKFAGASPSIVSGLAALREQIRGPTVTFTMDPRTSRAAITALIRLWMM 119
Query 151 SGAASFVVLCTHRGHGEKGHSVRCAVL 177
+ S V++ TH   + GH ++ +L
Sbjct 120 FNSVSPVIVVTHLEVPDVGHHLKGRLL 146
>coronafacic acid synthetase [Paraburkholderia acidicola]
Sequence ID: WP_096724586.1 Length: 179
Range 1: 17 to 150
Score:73.6 bits(179), Expect:8e-13,
Method:Compositional matrix adjust.,
Identities:43/137(31%), Positives:72/137(52%), Gaps:3/137(2%)
Query 41   PSLYADPLAWLVADAAESALTQCATEVLAAREDVAVILVSAHCTLHTMDAMARSLPAPRV 100
PSL+A PLAW +D ++ + + A E+ + + +I+VS C+L T+ +A +   ++

Sbjct 17 PSLFATPLAWATSDFVQTLIDE-AGEIQPS--ETGMIVVSDDCSLDTVRELAATAVQGKL 73
Query 101 SPLRFAGANPGSAGSLLCLLHGFKGSSLTLSMTPEDGLPAARTVARSWLRSGAASFVVLC 160
SPLRFAGA+P L L G +G +L L+M+PE + + WL ++
Sbjct 74 SPLRFAGASPSIVVGLPALQQGIRGPTLALTMSPEHAVAPVIALITYWLTHSGIDAAIVV 133
Query 161 THRGHGEKGHSVRCAVL 177
H HG + H + V+
Sbjct 134 AHHRHGVRSHLFKGLVV 150
